# Supramolecular polymers with reversed viscosity/temperature profile for application in motor oils

**DOI:** 10.3762/bjoc.17.11

**Published:** 2021-01-12

**Authors:** Jan-Erik Ostwaldt, Christoph Hirschhäuser, Stefan K Maier, Carsten Schmuck, Jochen Niemeyer

**Affiliations:** 1 Faculty of Chemistry (Organic Chemistry) and Centre for Nanointegration Duisburg- Essen (CENIDE), University of Duisburg-Essen, Universitätsstraße 7, 45141 Essen, Germany; 2 Evonik Operations GmbH, Kirschenallee, 64293 Darmstadt, Germany

**Keywords:** noncovalent interactions, polymers, ring-chain transformation, supramolecular chemistry, viscosity

## Abstract

We report novel supramolecular polymers, which possess a reversed viscosity/temperature profile. To this end, we developed a series of ditopic monomers featuring two self-complementary binding sites, either the guanidiniocarbonyl pyrrole carboxylic acid (GCP) or the aminopyridine carbonyl pyrrole carboxylic acid (ACP). At low temperatures, small cyclic structures are formed. However, at elevated temperatures, a ring–chain transformation leads to the formation of a supramolecular polymer. We demonstrate that this effect is dependent on the concentration of the solution and on the polarity of the solvent. This effect can counteract the loss of viscosity of the solvent at elevated temperatures, thus opening an application of our systems as viscosity index improvers (VIIs) in working fluids. This was tested for different motor oils and led to the identification of one compound as a promising VII.

## Introduction

Viscosity index improvers (VIIs) are used to counteract the loss of viscosity of working fluids (such as motor oils) at elevated temperatures, e.g., caused by engine heat. Common VIIs are based on poly(methyl acrylate) (PMA) [1]. These systems are able to increase the viscosity at elevated temperatures by increasing their hydrodynamic radius [2]. This is based on the fact that PMAs occur as coiled polymer chains with lower hydrodynamic radii at lower temperature, while elevating the temperature leads to uncoiling of the polymer chain [3]. This results in a larger hydrodynamic radii and thus in an increase of viscosity [4]. The quantitative description of the viscosity improvement was developed by Dean and Davis in 1929 and coined the “viscosity index”, based on the relative viscosities at 40 °C and 100 °C [5]. In motor oils, typical numbers for the viscosity index of PMA-based VIIs are in the range of 100–200 [6].

PMAs have been used as VIIs for decades and are still used today for motor oil viscosity improvement [7]. PMA-based VIIs are highly cost effective due to their simple chemical nature. However, this also prevents their further development on a molecular level. For this reason, we set out to develop novel VIIs based on the formation of supramolecular polymers from low-molecular weight building blocks. This would allow for an in-depth investigation of structure–property relationships based on a variation of the building bock [8]. Therefore, a deeper understanding on the influences of different molecular parts on VII performance would be possible and thus enable the design of taylor-made VIIs for different applications.

**Figure 1:**
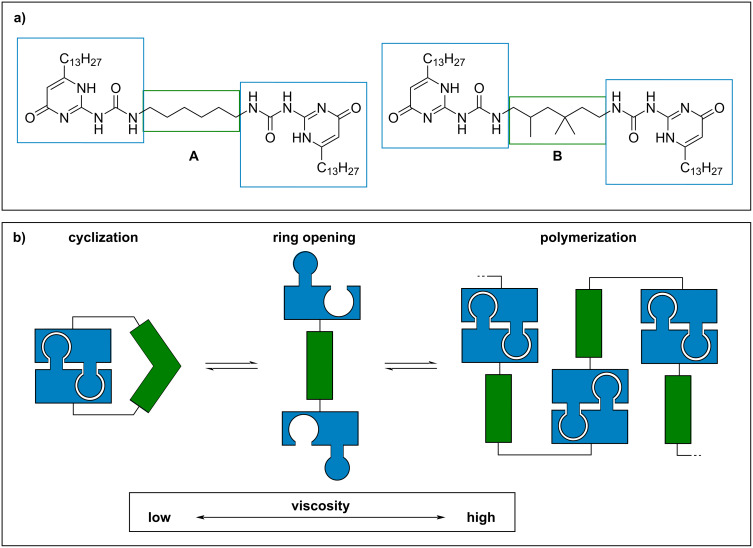
a) VII systems described by Sijbesma and Meijer, featuring two ureidopyrimidone BUs which are linked either via a linear hexylene linker (A) or via a branched 2,4,4-trimethylhexylene linker (B). b) Schematic mechanism of a supramolecular ring–chain polymerization.

To develop such novel VIIs, we envisaged the use of ditopic building blocks, which are able to reversibly form supramolecular polymers at higher temperatures. Supramolecular polymerization should increase the hydrodynamic radius and thus was expected to lead to increased viscosities [9]. There are different mechanisms for the formation of supramolecular polymers from molecular building blocks: The isodesmic, the cooperative and the ring–chain-mediated supramolecular polymerization [10]. For an application in viscosity improvement, a higher degree of polymerization is desired at elevated temperatures, which can be achieved by a ring–chain-based polymerization [11]. In this context, Sijbesma and Meijer reported an early example of a low-molecular weight VII using a ditopic monomer which undergoes supramolecular polymerization at elevated temperatures (Figure 1) [12–13]. The system consists of two self-complementary [14] binding units (BU, blue) and a connecting linker unit (LU, green) to connect the BUs. In this case, the BUs are ureidopyrimidone units, which dimerize due to a fourfold hydrogen bond, thus allowing for strong supramolecular interactions [15]. Due to the flexibility of the LUs, the BUs can interact with each other and form cyclic structures. At increased temperatures, a ring–chain polymerization takes place and the monomeric compounds undergo supramolecular polymerization, leading to a viscosity improvement in chloroform. In this study, it was also shown that LU needs to have a precoordinating effect to favor the cyclic form at lower temperatures. Thus, the alkylene-bridged monomer **A** shows no effect in terms of viscosity improvement. In contrast, the *gem*-dimethyl unit in monomer **B** leads to sufficient stabilization of the cyclic form at lower temperatures. This allows a ring–chain transformation at elevated temperatures, leading to the desired viscosity effect. The direct comparison of the linkers in **A** and **B** strongly suggests that the Thorpe–Ingold effect induced by the *gem*-dimethyl unit is crucial for the supramolecular polymerization, thus indicating that indeed a ring–chain transformation is occurring. Next to the system described by Sijbesma and Meijer, other compounds have been described that influence the viscosity by dynamic supramolecular interactions, also leading to a viscosity increase at elevated temperatures [16–18].

Inspired by these pioneering works of Sijbesma and Meijer, we now developed a series of novel ditopic monomers as potential VIIs. We conducted a systematic variation of both the linker unit and the binding unit in order to investigate structure–property relationships with regard to VII performance and solubility in nonpolar solvents (such as motor oils). This led to the identification of one final candidate that was successfully applied as a VII in two motor oils, which is unprecedented for a low-molecular weight VII.

## Results and Discussion

### Developing a VII system based on supramolecular interactions

**Figure 2:**
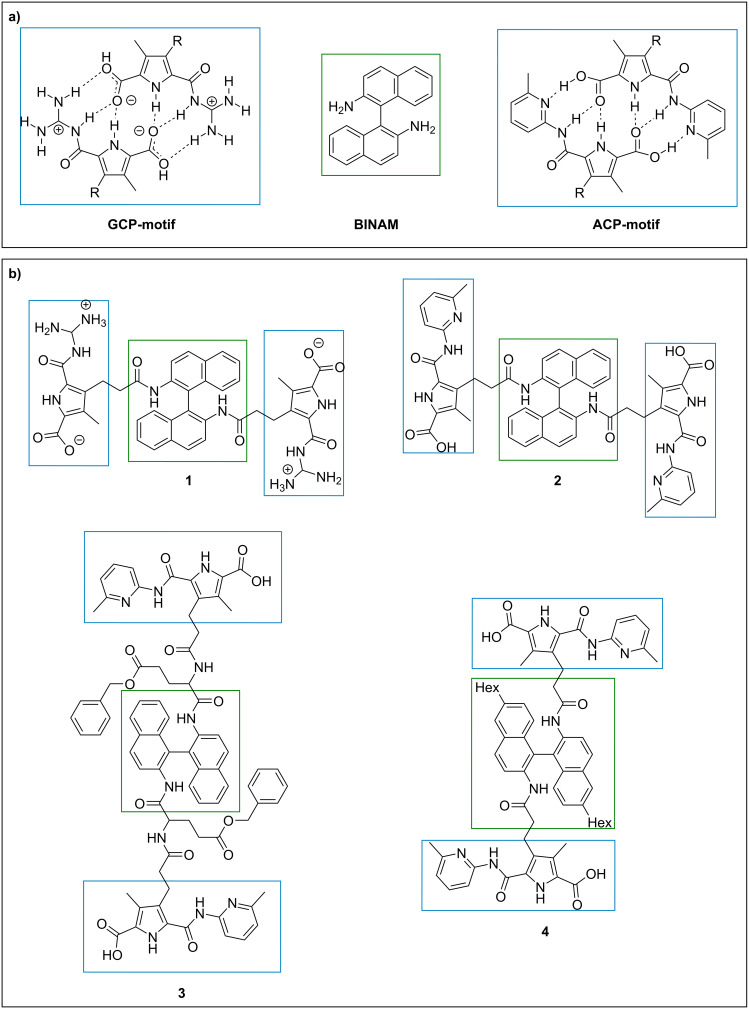
a) GCP and ACP motif, as charged and neutral BUs and BINAM as precoordinating LU. b) Compounds **1**, **2**, **3** and **4** are based on the combination of BINAM with the GCP or ACP motif, respectively.

Based on our working hypothesis, a supramolecular VII system requires a BU with strong dimerization tendency and a LU with precoordinating properties. For our purpose, we initially employed the guanidiniocarbonyl pyrrole (GCP) motif as a binding unit [19]. The GCP unit is a self-complementary zwitterion, which forms dimers with high association constants, based on a sixfold hydrogen-bonding interaction, strengthened by coulombic interactions (see Figure 2a) [20–21]. The GCP motif has successfully been applied for the formation of supramolecular polymers in earlier works [22–25].

The ionic nature of the GCP motif allows self-pairing even in polar solvents such as DMSO (*K*_a_ > 10^10^ M^−1^) and water (*K*_a_ > 10^2^ M^−1^) [26], but it also limits its solubility in nonpolar solvents. Therefore, another BU was also employed in this study, namely the less polar aminopyridine carbonyl pyrrole carboxylic acid (ACP). The ACP motif (Figure 2a) can be seen as a neutral analogue of the GCP motif. Due to the missing ionic interactions, the self-association is weaker, but still sufficiently strong, in aprotic solvents such as chloroform (*K*_a_ > 10^6^ M^−1^) [27]. As for the LU, we chose the 1,1’-binaphthyl-2,2’-diamine (BINAM) backbone. The attachment of the BUs via the amino group should result in the necessary concave prearrangement [28].

Force field calculations, carried out in order to determine the optimal distance between LU and BU (see Supporting Information File 1, chapter 8), show that a propionamide is the shortest linker that allows for cyclisation. Thus, all compounds feature a central BINAM unit, which is connected to the BUs (GCP or ACP) via a propionamide linker [29].

**Figure 3:**
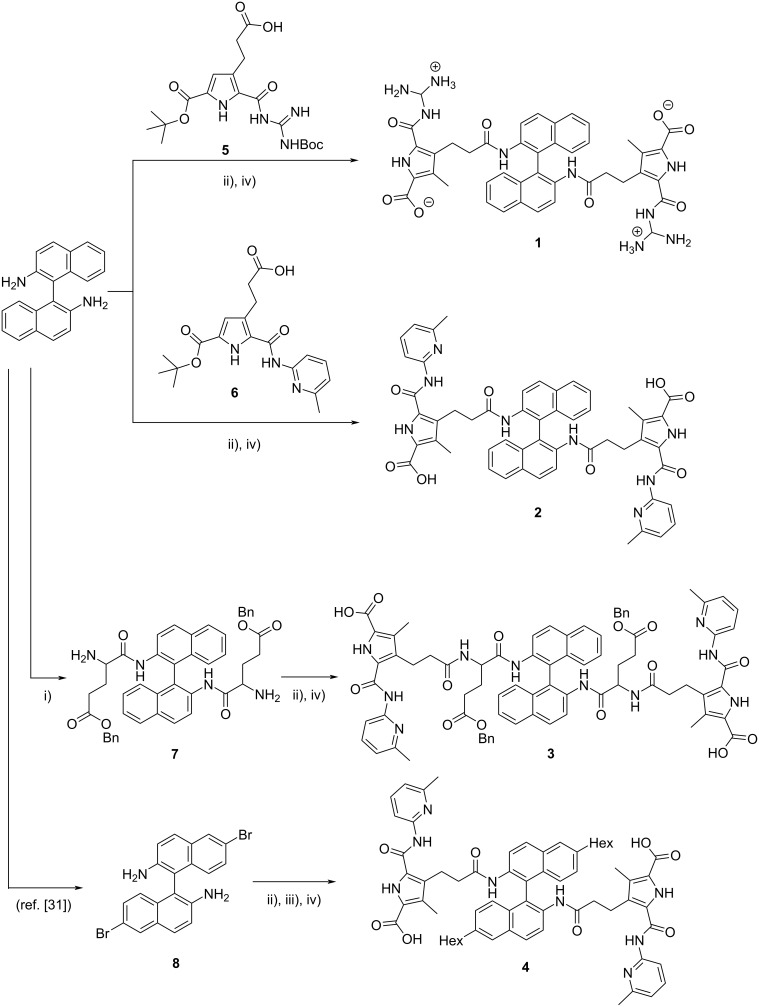
Synthesis of compounds **1** to **4**. Reagents and conditions: i) ʟ-Boc-glutamic acid benzyl ester, HCTU, DMAP, then TFA, 56% over two steps; ii) **6** (**5** for **1**), oxalyl chloride, then BINAM (for **1** and **2**) or **7** (for **3**) or **8** (for **4**), 50–81%; iii) Hex-MgBr, NiCl_2_, dppp, 78%; iv) TFA, 80–96%.

The synthesis of compound **1** was carried out starting from BINAM by coupling with GCP derivate **5** [12–13] after activation with thionyl chloride (see Figure 3). Deprotection with TFA yielded the bis-GCP derivate **1** in 40% yield over two steps. Accordingly, the ACP derivative **6** was coupled with BINAM, followed by deprotection to give bis-ACP derivative **2** (60% yield over two steps). For the extension of the linker unit, BINAM was first coupled with glutamic acid (*O*-benzyl and *N*-Boc protected). Removal of the Boc groups, coupling with the ACP precursor **6** and final deprotection gave the extended bis-ACP compound **3** (44% yield over 4 steps). Finally, BINAM was first brominated in the 6-position to give **8** [30], followed by coupling with the ACP precursor **6**. This allowed the subsequent introduction of hexyl groups by Kumada coupling. Deprotection then yielded the 6,6’-dihexyl-substituted bis-ACP derivative **4** (37% yield over 3 steps from **8**). All compounds were fully characterized by standard analytical methods (see Supporting Information File 1) [31].

After the synthesis, viscosity measurements were carried out. In the following the results are divided in two parts: the first part is dealing with the results of compounds **1** and **2**. Since compounds **3** and **4** are a further development of the first two compounds, a detailed study follows in the second part. The viscosity effects were studied via a falling sphere viscometer. Since the desired reversed viscosity/temperature effect (RVT effect) is only present in a certain concentration range, all viscosity measurements were done as a 2D-screening in dependence of both concentration and temperature. At first, these measurements were carried out in DMSO, because this was the only solvent where compounds **1** and **2** are equally soluble in. The RVT effect was barely observable in the kinematic viscosity, so that it was analyzed by studying the specific viscosities. The specific viscosity represents the viscosity in relation to the pure solvent and therefore describes the behavior of the additive.

**Figure 4:**
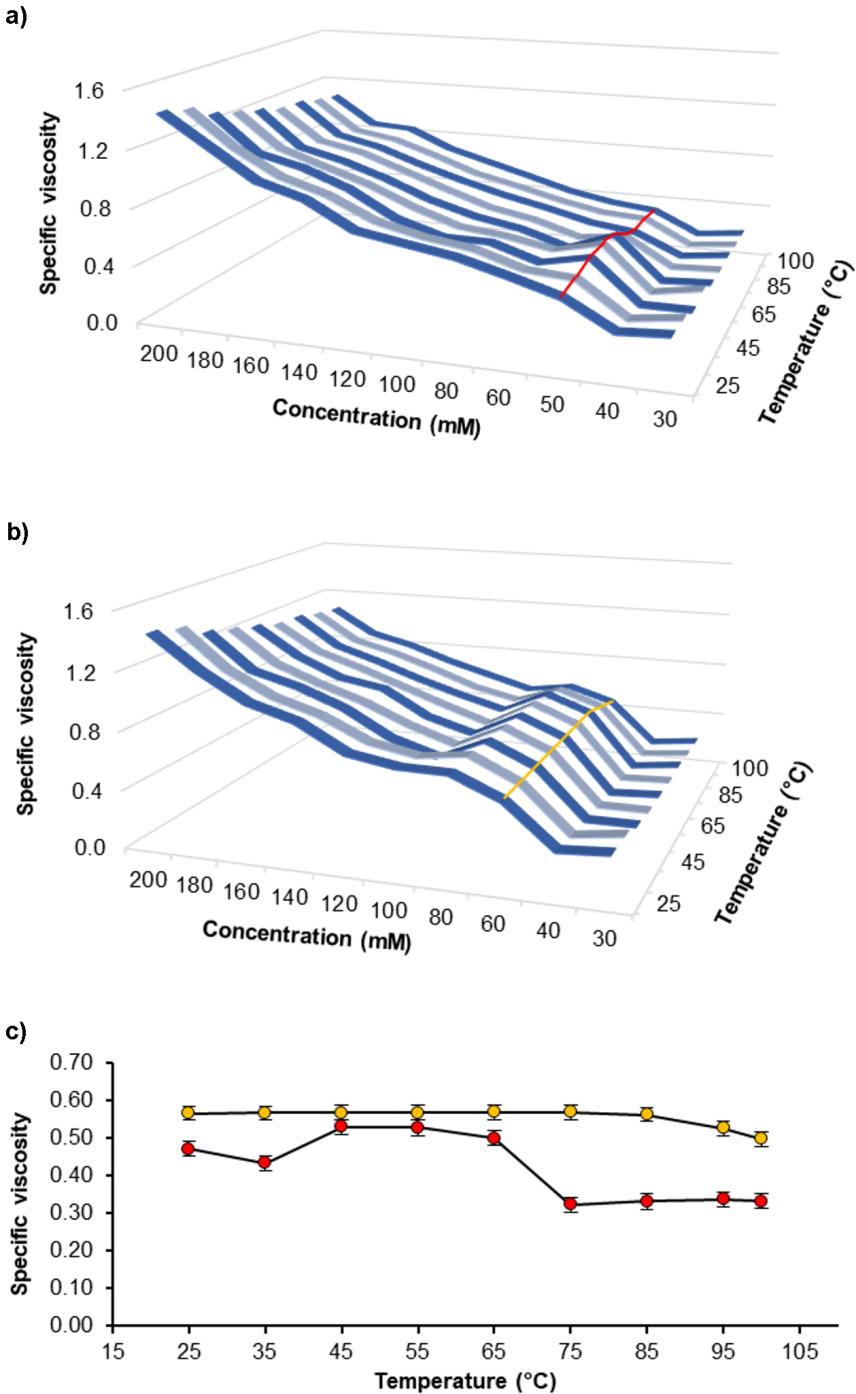
a) 2D-screening in DMSO of the GCP derivative **1**, specific viscosity vs concentration vs temperature. b) 2D-screening in DMSO of the ACP derivative **2**, specific viscosity vs concentration vs temperature. c) Comparison of the specific viscosities of the GCP derivative (red) at 50 mM and the ACP derivative (orange) at 60 mM in the concentrations where a RVT effect occurs in DMSO.

For both derivatives, a concentration/temperature area with an irregular viscosity behavior can be observed (see Figure 4). For the GCP derivative **1**, a concentration of 50 mM leads to an RVT effect at temperatures between 45–65 °C, but the specific viscosity decreases at lower or higher temperatures (Figure 4a,c). Surprisingly, the ACP derivative **2** does not show such a distinct maximum, but the specific viscosity remains almost constant in the temperature range of 25–85 °C. Only above 85 °C, a decrease in specific viscosity is observed (Figure 4b,c). The 2D-plots of viscosity vs concentration underline the very narrow concentration range where an unusually high viscosity is found, whereas otherwise a steady increase in viscosity with increasing concentrations is found (see Supporting Information File 1, Figures S18 and S32).

These investigations demonstrate that the desired RVT effect is only present in a narrow concentration regime, which has to be determined individually for each compound. This was rationalized as follows: If the concentration is too low, cyclic structures are predominant over the entire temperature range. In contrast to that, if the concentration is too high, supramolecular polymers are even formed at lower temperatures. Thus, the desired ring–chain transformation upon increasing the temperature is only effective in a specific concentration range.

**Figure 5:**
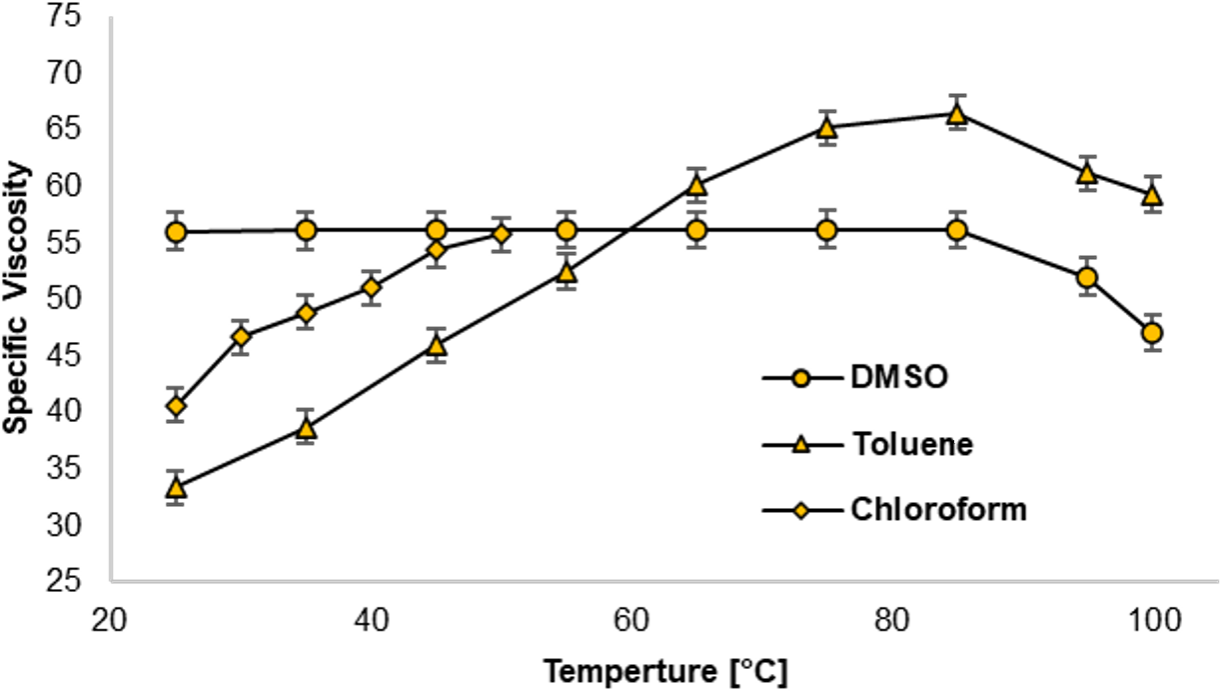
Comparison of the specific viscosities in dependence of the temperature of the ACP derivative (orange) in three different solvents at a concentration of 60 mM.

Based on these initial promising results, we conducted a more detailed investigation of compounds **1** and **2**. For the desired application, an RVT effect in nonpolar solvents (like motor oils) is desired, which might lead to a different behavior than that observed in DMSO. Unfortunately, the GCP derivative **1** is not soluble in nonpolar solvents, such as chloroform, toluene or motor oils. Yet, we were able to investigate the behavior of the ACP derivative **2** in chloroform and toluene (see Figure 5). For both solvents, screening for the optimum concentration range was repeated (see Supporting Information File 1, chapter 6.2). In both chloroform and toluene solution, an RVT effect occurred at a concentration of 60 mM, as found in DMSO.

However, the RVT effect is significantly more pronounced in less polar solvents (Figure 5). Over the whole temperature range in chloroform (25–50 °C), we observed an increase of the specific viscosity. In toluene, a broader temperature range (up to 100 °C) could be investigated due to its higher boiling point. Here, a steady increase in specific viscosity can be observed between 25–85 °C, and only above 85 °C the specific viscosity decreases slightly.

**Figure 6:**
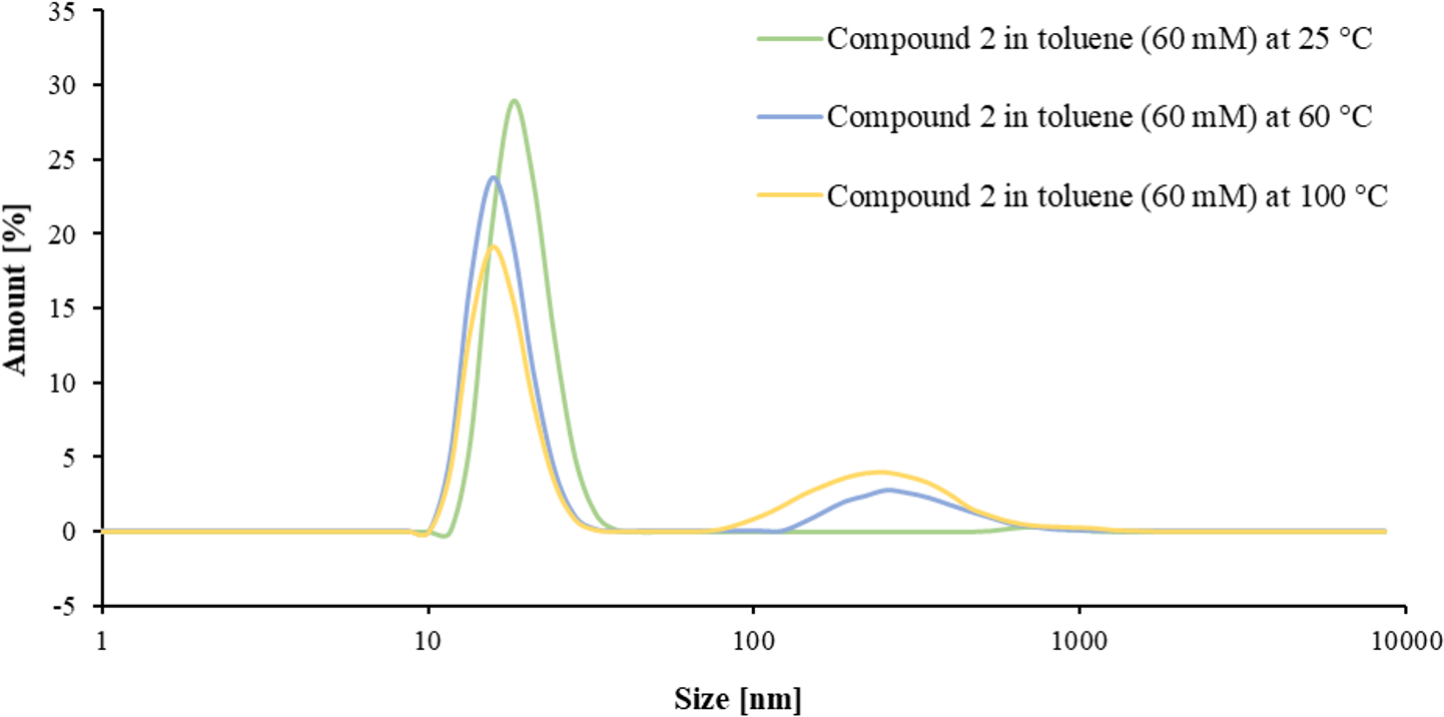
DLS measurement of compound **2** in toluene at 25 °C, 60 °C and 100 °C.

The formation of larger structures for **1** and **2** was also confirmed by DLS (see Figure 6 for compound **2** in toluene, see Supporting Information File 1, chapter 7 for compound **1** and other solvents). Here, an effect of the temperature on the hydrodynamic radius can be observed. We find two different peak areas, one with sizes of 10–40 nm and one with 100–700 nm. At 25 °C, the peak area at smaller sizes amounts to 99%, showing that almost no larger structures are present. Elevating the temperature leads to the formation of larger structures, indicated by a change in the relative peak areas (81%/65% for the 10–40 nm peak at 60 °C/100 °C, accordingly 19%/35% for the 100–700 nm peak at 60 °C/100 °C). Here we assume the formation of a mixture of differently sized cyclic structures, that are represented by the signal at 10–40 nm. Elevating the temperature leads to the formation of oligomers and polymers that possess larger hydrodynamic radii. Interestingly, the peak representing the smaller structures not only decreases, but also slightly shifts to even smaller numbers in terms of size. This shows that especially medium-sized rings are transformed into polymeric structures, while smaller rings seem to be more stable. The formation of such larger structures also explains the increasing specific viscosity in elevated temperatures. For this reason, both compounds are able to counteract the loss of viscosity at increasing temperatures and can thus act as VIIs.

These results indicate that the combination of the BINAM backbone with either the GCP or the ACP motif is suitable to provide the desired RVT effect. However, for the desired application, a substantial solubility in motor oils is necessary. In this study, two motor oils were tested: Nynas NS8 (a motor oil based on naphthenic structures) and Nexbase 3020 (a motor oil based on C_15_ to C_30_ alkanes). Unfortunately, GCP derivative **1** is neither soluble in nonpolar solvents (chloroform, toluene) nor in any of the motor oils. In contrast, ACP derivative **2** shows good solubility in chloroform and toluene, and even shows limited solubility in Nynas NS8 (vide infra). However, even compound **2** is insoluble in Nexbase 3020. For this reason, we performed structural modifications of our ditopic monomers in order to further increase the solubility, whilst hoping to preserve the RVT effect.

### Optimization of a VII on motor oil

Since compounds **1** and **2** are hardly soluble in the motor oils investigated here (Nynas NS8 and Nexbase 3020), we performed structural modifications in order to increase the solubility. Keeping the BINAM backbone and the ACP binding unit, we performed two modifications (as described above, see Figure 2b): First, the linker unit was extended by insertion of a benzyl-protected glutamic acid between the BINAM core and the ACP unit to give compound **3**. Secondly, hexyl substituents were installed in the periphery of the BINAM core via bromination and cross-coupling, giving compound **4**. In both cases, the additional nonpolar substituents were expected to increase the solubility in nonpolar solvents.

Thus, the solubility of compounds **3** and **4** in the two motor oils was tested and compared to that of compounds **1** and **2** (see Table 1). While compound **2** shows some solubility in Nynas NS8 (60 mM), but is insoluble in Nexbase 3020 (vide supra), both compounds **3** and **4** show a significantly higher solubility in the motor oils (100/>200 mM in Nynas NS8, 40/90 mM in Nexbase 3020 for compounds **3**/**4**). Thus, we were able to investigate the RVT effect of compounds **2**, **3** and **4** in the corresponding motor oils (for the viscosity behavior of **3** and **4** in DMSO, chloroform and toluene see Supporting Information File 1, chapters 6.3 and 6.4).

**Table 1:** Maximum concentration of each compound in Nynas NS8 and Nexbase 3020.

Compound	Solubility in:	

	Nynas NS8	Nexbase 3020

**1**	<5 mM	<5 mM
**2**	60 mM	<5 mM
**3**	100 mM	40 mM
**4**	≥200 mM	90 mM

**Figure 7:**
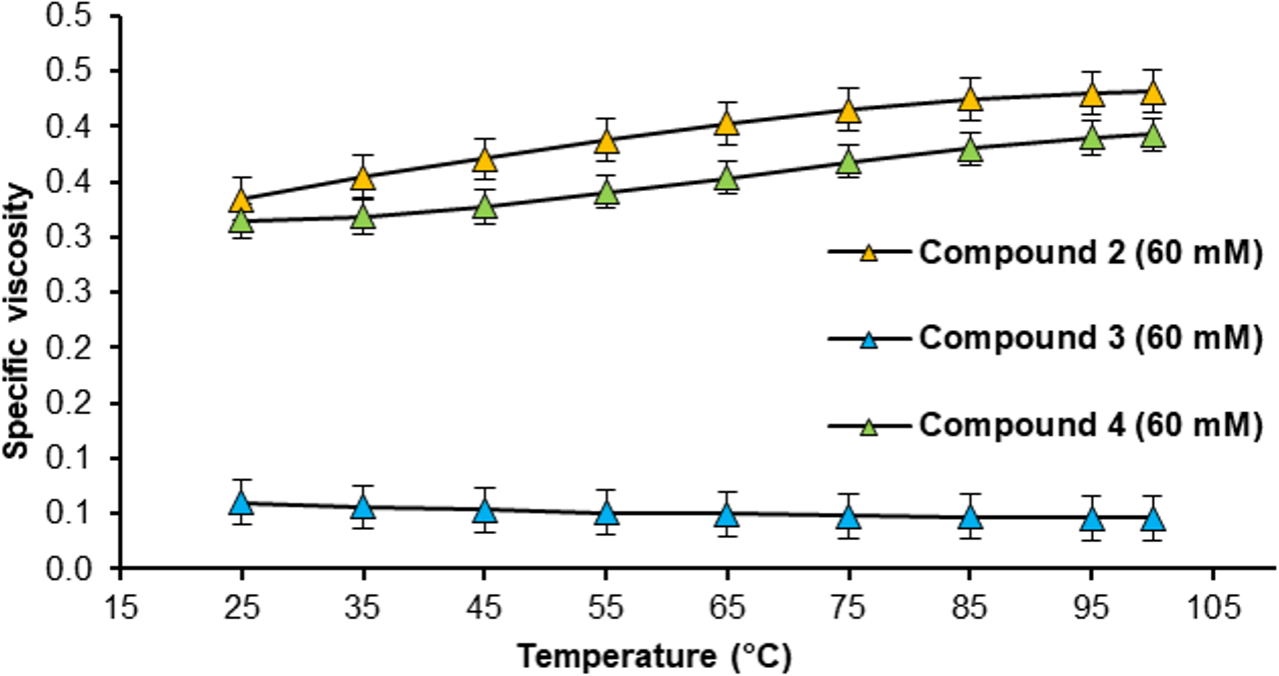
Specific viscosity of compounds **2**, **3** and **4** in Nynas NS8 in dependency to the temperature.

Starting with the measurements in Nynas NS8, compounds **2**, **3** and **4** were tested at 60 mM concentration. Compounds **2** and **4** behave in a very similar fashion and show an increase in specific viscosity over the entire temperature range (25–100 °C). In strong contrast, compound **3** does not show an RVT effect and the specific viscosity decreases significantly upon increasing the temperature (the same was also observed in toluene solution, see Supporting Information File 1, chapter 6.3.2). Thus, the modification of the linking unit by insertion of one amino acid (to give compound **3**) has a detrimental effect on the RVT effect (Figure 7). Most likely, extension of the linker-unit tips the balance between cyclic monomers and the supramolecular polymer. For this reason, no ring–chain transition takes place and no RVT effect could be established.

**Figure 8:**
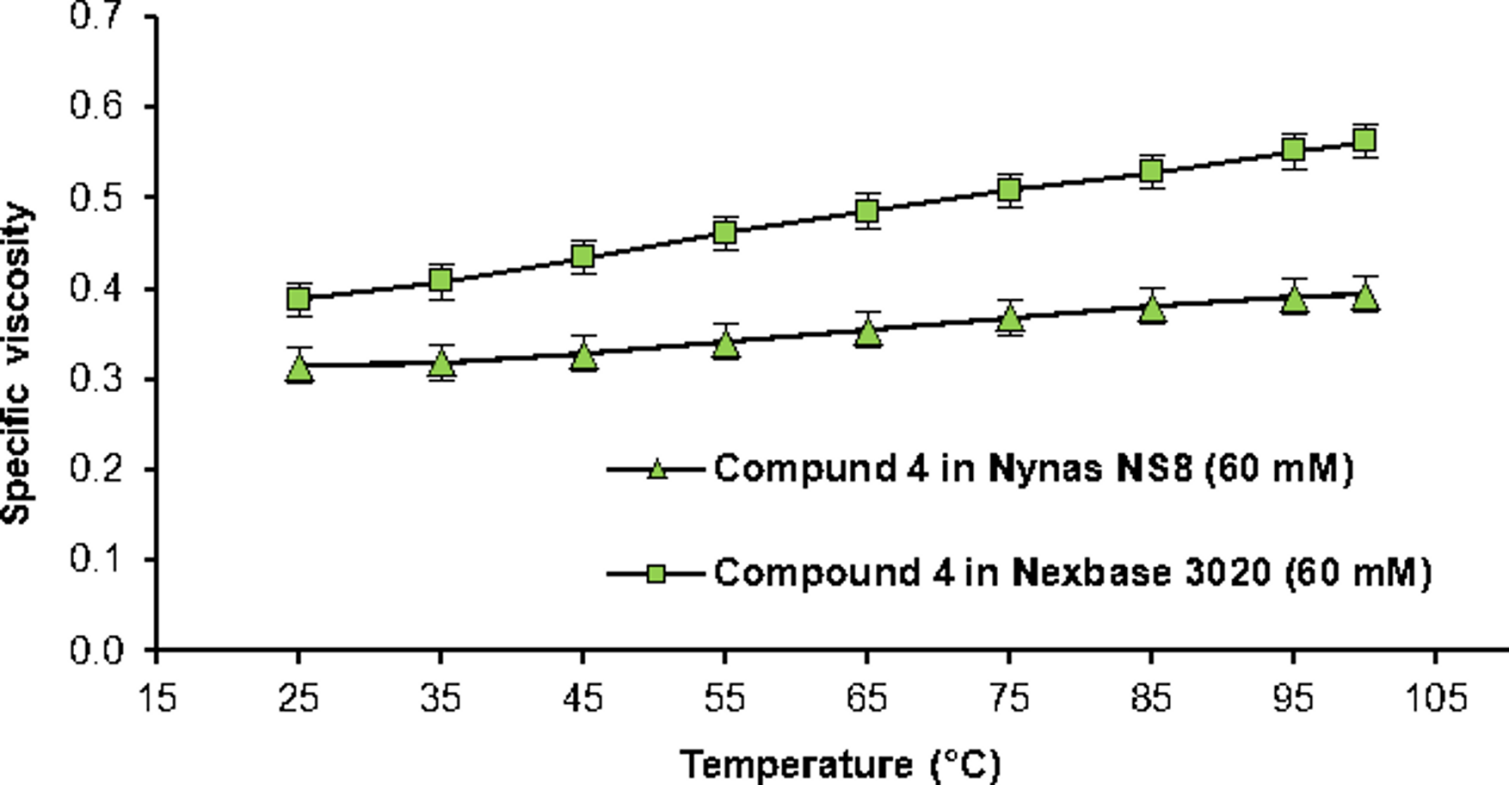
Specific viscosity of compound **4** in Nynas NS8 and Nexbase 3020*.*

Thus, we focused our investigation on compound **4**, which seems to possess the correct distance between the BINAM core and the ACP binding unit for the RVT effect, while being significantly more soluble than compound **2** due to the additional hexyl substituents. This allowed its use in Nexbase 3020 (at 60 mM concentration) in a final application test. As shown in Figure 8, the specific viscosity in Nexbase 3020 is not only higher than in Nynas NS8, but it also shows a stronger increase with elevating temperatures (+45% in Nexbase 3020, cf., +25% in Nynas NS8 between 25 °C and 100 °C).

Finally, we determined the viscosity index (VI) as defined by Dean and Davis. The kinematic viscosities of 40 °C and 100 °C are used to calculate the VI, using databases like viscosity index calculators of Evonik [32]. While the VI of Nynas NS8 was increased by 100% (VI 47 to 94) using compound **2**, compound **4** was able to increase its VI by 89% (VI 47 to 89). In contrast to that, the VI of Nexbase 3020 was increased by 73% (VI 88 to 152) using compound **4**. These results firmly establish compounds **2** and **4** as efficient VIIs.

## Conclusion

In summary, we developed a new monomeric unit which forms polymeric structures at higher temperatures. This effect depends strongly on the concentration and the precise structural requirements. Too low concentrations prevent polymerization of monomeric units. Too high concentrations lead to polymerization independently of the temperature. In terms of structural requirements, a strongly precoordinating LU and a precise distance between LU and BU (cf., compound **3** and **4**) were necessary. The employed GCP and ACP binding units turned out to be ideal for self-complimentary systems like this. In addition to optimizing concentration and temperature, the desired VI effect is also dependent on the solvent, as the RVT effect was stronger in nonpolar solvents. This behavior is very useful for VII applications in motor oil. Here, our system could compensate for the loss of viscosity at higher temperatures. This RVT effect was showcased in two motor oils (Nynas NS8 and Nexbase 3020), in which compounds **2** and **4** lead to a significant increase of the specific viscosity and VI of Nynas NS8. In Nexbase 3020, compound **4** showed an even higher RVT effect. Therefore, the development of a low molecular-weight monomer that undergoes supramolecular polymerization at elevated temperatures and can thus act as a viscosity index improver was successfully realized.

## Experimental

All experimental details can be found in Supporting Information File 1.

## Supporting Information

File 1 Experimental part.
