# Supplementary material for: Supramolecular polymers with reversed viscosity/temperature profile for application in motor oils
Source: Beilstein J Org Chem. 2021 Jan 12;17:105–14. doi: 10.3762/bjoc.17.11 (PMC7814181; doi:10.3762/bjoc.17.11)
Supplement: File 1 — Experimental part. [file Beilstein_J_Org_Chem-17-105-s001.pdf]

BEILSTEIN JOURNAL OF ORGANIC CHEMISTRY

Supporting Information

for

Supramolecular polymers with reversed viscosity/temperature profile for application in motor oils

Jan-Erik Ostwaldt, Christoph Hirschhäuser, Stefan K. Maier, Carsten Schmuck and Jochen Niemeyer

Beilstein J. Org. Chem. **2021,** *17,* 105–114. doi:10.3762/bjoc.17.11

Experimental part

License and Terms: This is a supporting information file under the terms of the Creative Commons Attribution License (https://creativecommons.org/licenses/by/4.0). Please note that the reuse, redistribution and reproduction in particular requires that the author(s) and source are credited and that individual graphics may be subject to special legal provisions.
The license is subject to the *Beilstein Journal of Organic Chemistry* terms and conditions: (https://www.beilstein-journals.org/bjoc/terms)

Table of Contents:

1. General S2

2. Synthesis of the BINAM-GCP motif 1 ....S5

3. Synthesis of the BINAM-GCP motif 2 ....S7

4. Synthesis of the amino acid extended BINAM-ACP 3 ....S11

5. Synthesis of the substituted BINAM-GCP motif 4 ....S15

6. Viscosity measurements S18

7. DLS Measurements S48

8. Force field calculations S52

9. NMR-Spectra S54

1. General

1.1 Synthesis

Each chemical mentioned is commercially available and was used without purification unless specified. All reactions were carried out under an atmosphere of argon in a pre-heated flask and all chemicals were added in argon flow. THF (Tetrahydrofuran) was freshly distilled from sodium/benzophenone. DCM (dichloromethane) and DMF (*N*,*N*-dimethylformamide) were freshly distilled from CaH_2. Diisopropylethylamine was dried over CaH_2 by refluxing for 72 h after which it was distilled under an atmosphere of argon and stored over molecular sieve under an atmosphere of argon.

^{1}H- and ^{13}C NMR spectra were recorded in $CDCl_3$ and DMSO-d_6 on Bruker DMX 300, AV Neo 400 NMR and Bruker DMX 600 spectrometers.

IR spectra were measured on a Jasco FT/IR-430 with ATR attachment spectrometer.

Low and High resolution ESI mass spectra were recorded with a Bruker amaZon SL and a Bruker maXis 4G spectrometer, respectively.

For each chiral compound, the (*S*)-enantiomer, e.g., (*S*)-BINAM and L-Glutamic acid, was used.

For Flash chromatography columns were packed with Silica gel 60 M (40–63 µm) from *Macherey-Nagel*.

Medium performance liquid chromatography (MPLC) was carried out using *Armen Instrument Liquid Chromatography Flash Apparatus*. Packed columns of *Kronlab* were used with RP18 from *YMC*. (YMC-ODS-AQ: 60 cm length, 3 cm diameter, 5 µm particle size, 12 nm pore size)

1.2 Viscosity measurements

The viscosity measurements were carried out in a "Rolling-ball viscometer Lovis 2000 M" by Anton Paar with a sample amount of 100 µL to 200 µL and a temperature range of −30 °C to 100 °C. All viscosity measurements were carried out at neutral pH, which was adjusted if necessary, by adding aqueous 0.1 N NaOH or 0.1 N HCl, monitored by pH-Meter 766 from Knickarray.

For each sample (compound and concentration) three different solutions were prepared and filtered through syringe filters (0.4 µm). Each solution was measured heating up at 25 °C, 35 °C, 45 °C, 55 °C, 65 °C, 75 °C, 85 °C, 95 °C and 100 °C and cooling down at the same temperatures except for chloroform solutions, where the maximum temperature was 50 °C. Each data point was measured by the viscometer in a sequence of six times while varying the drop angle of the sphere and each sequence was done three times. After every measurement session, the sample holder was completely cooled down to room temperature and washed with acetone and cyclohexane, after which it was dried with compressed air. With this method, falling time, kinematic viscosity and dynamic viscosity were measured.

The specific viscosity was calculated with the kinematic viscosity of each solution and the pure solvent. Therefore, the viscosity of the pure solvent was measured the same way as described before. By the formula of the specific viscosity the percentage behaviour was obtained.

$$\eta_{Sp} = \frac{\eta_C - \eta_0}{\eta_0}$$

The specific viscosity (η_{Sp}) results out of the dynamic viscosity of each concentration (η_C) and the equivalent dynamic viscosity of the solvent (η_0) at each temperature.

1.3 Abbreviations

δ	chemical shift
°C	degree celsius
ACP	aminopyridine carbonyl pyrrole carboxylic acid
BINAM	1,1′-binaphthyl-2,2′-diamine
BINOL	1,1'-binaphthyl-2,2'-diol
Boc	*tert*-butyloxycarbonyl
tBu	*tert*-butyl
br	broad
c	concentration
calcd.	calculated
$CDCl_3$	deuterated chloroform
d	doublet / diameter/ day
DCM	dichloromethane
DIPEA	*N*,*N*´-diisopropylethyl amine
DLS	dynamic light scattering
DMF	*N*,*N*´-dimethyl Formamide
DMSO	dimethyl sulfoxide
DMSO- d_6	deuterated dimethyl sulfoxide
Glu	glutamic acid
e.g.	for example
equiv	equivalent
ESI-MS	electrospray ionization mass spectrometry
Et_3N	triethylamine
FT-IR	fourier transform infrared spectroscopy
g	gram
GCP	guanidiniocarbonyl pyrrole
h	hour (s)
HCTU	1-H-Benzotriazolium 1-[bis(dimethylamino)-methylene]-5chloro-hexafluorophosphate (1-),3-oxide
HPLC	high performance liquid chromatography
Hz	Hertz
m	milli / multiplett / meter
M	mol/L
m/z	mass per charge
Me	methyl
MeOH	methanol
MHz	megahertz
min	minute (s)
µM	micromolar
mM	millimolar
MPLC	medium performance liquid chromatography
MS	mass spectrometry
NMR	nuclear magnetic resonance
p	para
Pd/C	palladium on charcoal
ppm	parts per million
q	quadruplet
r	radius
rt	room temperature
s	singlet / second(s)
t	time
TFA	trifluoroacetic acid
THF	tetrahydrofuran
VI	viscosity index

2. Synthesis of the BINAM-GCP motif 1

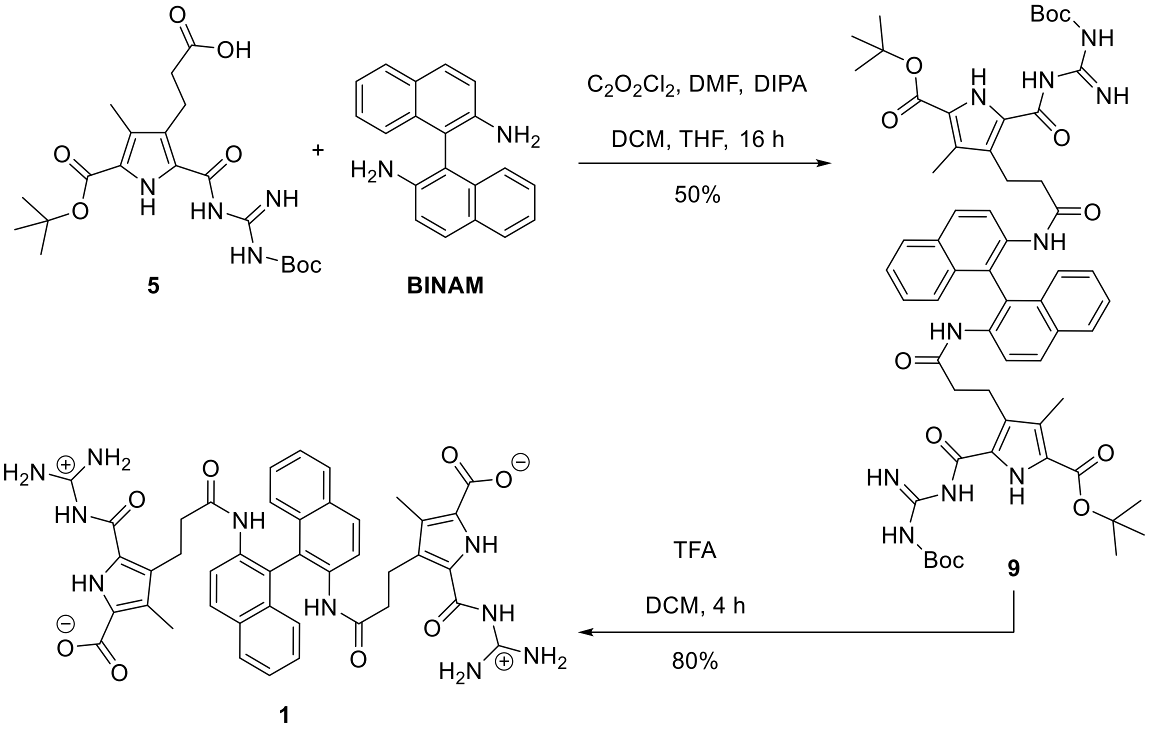

Scheme S1: Synthetic scheme of BINAM-GCP motif.

2.1 Synthesis of the protected GCP-BINAM motif 9

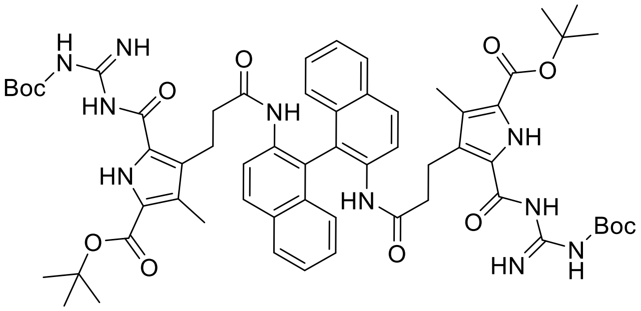

9

In a dried Schlenk tube the deprotected GCP building block **5**[1] (0.10 g, 0.228 mmol, 2.5 equiv) was dissolved in dry DCM (20 mL). DMF (0.5 mL) was added and the solution was cooled to 0 °C. At this temperature oxalyl chloride (24 µL, 34.7 mg, 0.273 mmol, 3 equiv) was added to give a red solution. The solution was stirred for three hours over which it was allowed to reach room temperature. Subsequently the solvents were removed in vacuo to give a light red foam, which was dissolved in dry THF (20 mL). This solution was added to a solution of *(S)*-1,1'-binaphthalene-2,2'-diamine (26.0 mg, 0.09 mmol, 1 equiv) and Diisopropylethylamine (76.6 µL, 58.2 mg, 0.450 mmol, 5 equiv) in THF (20 mL) at room temperature. The reaction mixture was stirred for 16 hours until a yellow suspension formed.

[1] C. Schmuck, D. Rupprecht, C. Urban and N. Walden, *Synthesis* **2006**, 89-96.

The solvent was removed in vacuo and the residue was dissolved in DCM (20 mL). The organic layer was washed with water (3 × 10 mL) and brine (3 × 10 mL) and dried over $MgSO_4$. Solvents were removed in vacuo. The product was purified by flash chromatography (ø = 3 cm, h = 30 cm, CyHex:EtOAc = 2:1) to give the pure **9** (51.2 mg, 0.0455 mmol, 50%) as a white solid.

R_f: 0.34 (CyHex:EtOAc = 2:1). **^{1}H-NMR:** (400 MHz, $CDCl_3$) δ [ppm] = 1.52 (s, 18 H, 2 x C-(C*H*$_3$)$_3$), 1.57 (s, 18 H, 2 x C-(C*H*$_3$)$_3$), 2.03 - 2.04 (m, 6 H, 2 x C*H*$_3$), 2.19 - 2.27 (m, 4 H*H*), 2.64 - 2.71 (m, 4 H, 2 x C*H*$_2$), 7.10 (d, ^3J = 8.5 Hz, 2 H, 2 x BINOL-*H*), 7.27 - 7.30 (m, 2 H, 2 x BINOL-*H*), 7.39 - 7.46 (m, 2 H, 2 x BINOL-*H*), 7.91 (d, ^3J = 8.1 Hz, 2 H, 2 x BINOL-*H*), 7.98 (d, ^3J = 9.00 Hz, 2 H, 2 x BINOL-*H*), 8.36 (d, ^3J = 8.9 Hz, 2 H, 2 x BINOL-*H*). **^{13}C-NMR:** (151 MHz, $CDCl_3$) δ [ppm] = 9.7, 21.4, 28.0, 28.4, 38.4, 81.3, 83.2, 121.5, 121.9, 122.5, 125.2, 125.6, 126.0, 127.2, 127.7, 127.9, 128.3, 129.5, 131.3, 132.3, 134.9, 153.0, 158.0, 160.4, 171.3, 172.9. **HR-MS:** (ESI, MeOH): m/z = 1125.5397 ([M+H]$^+$), calcd. 1125.5404 (for [$C_{60}H_{73}N_{10}O_{12}$]$^+$). **FT-IR:** (ATR): $\tilde{\nu}$ [cm^{-1}] = 3508, 3267, 3062, 2925, 2630, 1845, 1771, 1675, 1621, 1141, 1130, 1141, 1078, 895, 830, 681, 524. **Melting point:** 138 °C.

2.2 Deprotection of the BINAM-GCP-Motif 1

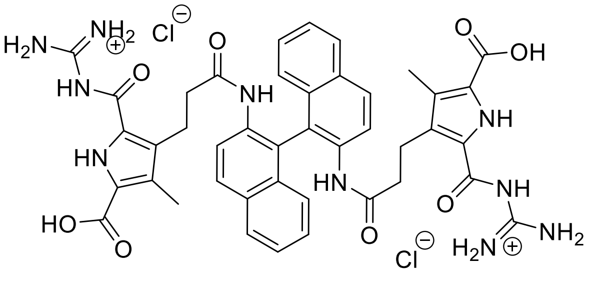

1

The *(S)*-BINAM-GCP-motif **9** (100 mg, 0.0889 mmol) was dissolved in DCM (5 mL) and cooled to 0 °C. Trifluoroacetic acid (2.5 mL) was added slowly and the mixture was allowed to warm up to room temperature over the course of one hour. The mixture was stirred at room temperature for an additional four hours and the solvent was removed in vacuo. Hydrochloric acid (1 M in H_2O, 10 mL) was added and the resulting solution was dried in vacuo. Product **1** (63.3 mg, 0.0715 mmol, 80%) was obtained as a white solid after purification by MPLC (ø = 3 cm, h = 60 cm, RP18, methanol:water = 1:10 to pure methanol).

R_f: 0.08 (CyHex:EtOAc = 1:1). **^{1}H-NMR:** (300 MHz, DMSO-*d6*) δ [ppm] = 1.89 - 1.94 (m, 6 H, 2 x CH_3), 2.15 - 2.17 (m, 4 H, 2 x C*H*$_2$), 2.35 - 2.43 (m, 4 H, 2 x C*H*$_2$), 7.03 (d, ^3J = 9.07 Hz, 2 H, 2 x BINOL-*H*), 7.29 (d, ^3J= 7.82 Hz, 1 H, 2 x BINOL-*H*), 7.49 (t, ^3J = 6.57 Hz, 2 H, 2 x BINOL-*H*), 7.82 (d, ^3J = 9.38 Hz, 2 H, 2 x BINOL-*H*), 8.03 (d, ^3J = 8.44 Hz, 1 H, 2 x BINOL-*H*), 8.09 (d, ^3J = 9.07 Hz, 2 H, 2 x BINOL-*H*), 8.24 - 8.33 (br m, 6 H), 11.02 (br s, 2 H), 11.85 (br s, 2 H), 13.18 (br s, 2 H). **^{13}C-NMR:** (151 MHz, DMSO-*d6*) δ [ppm] = 13.9, 22.1, 28.7, 120.4, 122.8, 123.5, 124.4, 124.7, 125.2, 126.5, 127.1, 127.7, 130.0, 130.4, 133.0, 134.2, 136.7, 154.3, 158.6, 161.7, 166.8. **HR-MS:** (ESI, MeOH): m/z = 813.3115 ([M+H$^+$-2HCl]$^+$), calcd. 813.3103 (for [$C_{42}H_{41}N_{10}O_8$]$^+$). **FT-IR:** (ATR): $\tilde{\nu}$ [cm^{-1}] 3531, 3054, 2867, 2627, 1773, 1691, 1660, 1650, 1120,1066, 1074,1070, 815, 777. **Elemental analysis:** calcd. (%) for $C_{42}H_{42}Cl_2N_{10}O_8$: C 56.95, H 4.78, N 15.81; found: C 52.80, H 3.91, N 11.20. **Melting point:** 166 °C.

3. Synthesis of the BINAM-GCP motif 2

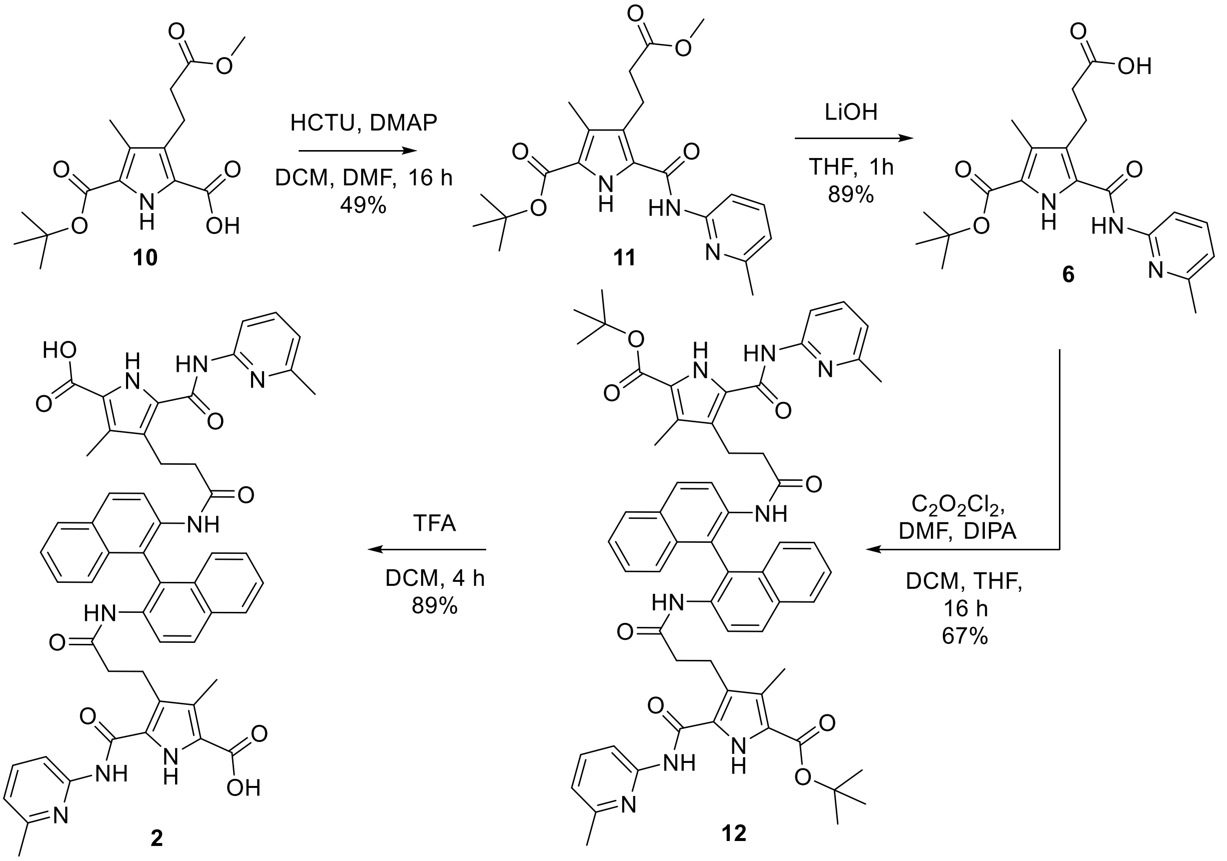

Scheme S2: Synthetic scheme of BINAM-ACP motif.

3.1 Synthesis of the ACP binding motif 11

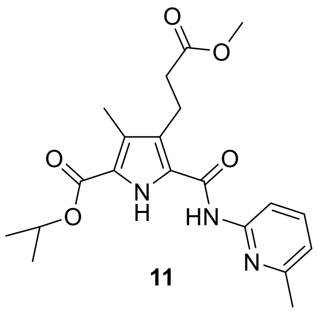

Pyrrole **10**[1] (1.00 g, 3.21 mmol, 1 equiv), HCTU (1.59 g, 3.85 mmol, 1.2 equiv) and DMAP (1.17 g, 9.63 mmol, 3 equiv) were dissolved in DCM (50 mL) and DMF (2 mL). After stirring for 30 minutes, 6-methylpyridin-2-amine (0.521 g, 4.81 mmol, 1.5 equiv) was added and the mixture was stirred for another 16 h at room temperature. The solvents were removed under reduced pressure and the residue was resolved in DCM (20 mL). After washing with water (3 × 10 mL) and brine (3 × 10 mL) the combined organic layers were evaporated and the residue was purified by flash chromatography (ø = 5 cm, h = 40 cm, CyHex:EtOAc = 5:1). Product **11** (0.630 g, 1.57 mmol, 49%) was obtained as a white solid.

R_f: 0.61 (CyHex:EtOAc = 5:1). **^{1}H-NMR:** (400 MHz, $CDCl_3$) δ [ppm] = 1.55 (s, 9 H, (C-C*H*$_3$)$_3$), 2.30 (s, 3 H, CH_3), 2.47 (s, 3 H, CH_3), 2.73 (t, ^3J = 7.0 Hz, 2 H, CH_2), 3.09 (t, ^3J = 7.0 Hz, 2 H, C*H*$_2$), 3.70 (s, 3 H, C*H*$_3$), 6.91 (d, ^3J = 7.5 Hz, 1 H, pyr-C*H*), 7.63 (dd, ^3J = 7.5, 8.2 Hz, 1 H, pyr-C*H*), 8.10 (d, ^3J = 8.2 Hz, 1 H, pyr-C*H*), 10.04 (s, 1 H, N*H*), 10.21 (br s, 1 H, N*H*). **^{13}C-NMR:** (151 MHz, $CDCl_3$) δ [ppm] = 10.4, 20.3, 23.8, 28.5, 33.9, 52.1, 81.8, 112.1, 119.3, 123.4, 124.8, 125.8, 126.5, 139.4, 151.2, 156.1,

159.9, 160.3, 174.2. **HR-MS:** (ESI, MeOH): m/z = 402.2036 ($[M+H]^+$), calcd. 402.2023 (for $[C_{21}H_{28}N_3O_5]^+$). **FT-IR:** (ATR): $\tilde{\nu}$ [cm^{-1}] = 3332, 3301, 2923, 1695, 1664, 1604, 1569, 1542, 1438, 1365, 1336, 1303, 1278, 1249, 1213, 1155, 1135, 1078, 983, 842, 779, 709, 609. **Melting Point:** 101 °C.

3.2 Deprotection of the ACP building block 6

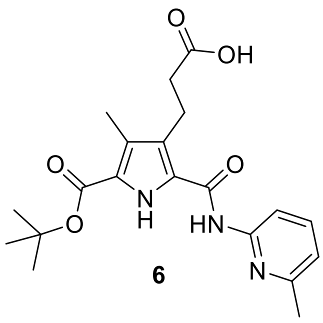

6

To a solution of the protected ACP building block **11** (1.00 g, 2.49 mmol, 1 equiv) in THF (50 mL) $LiOH\cdot H_2O$ (0.418 g, 9.96 mmol, 4 equiv) in water (20 mL) was added. The reaction mixture was stirred for 1 h at 0 °C and 8 h at room temperature. The mixture was diluted with water (10 mL) and washed with Et_2O (3 × 30 mL). The solution was acidified by 0.5 M $NaHSO_4$ (10 mL) and directly extracted with EtOAc (3 × 30 mL). All organic layers were collected, washed with brine (3 × 30 mL) and dried with $MgSO_4$. Solvents were removed in vacuo. The product **6** (0.860 g, 2.22 mmol, 89%) was obtained as a white powder.

R_f: 0.22 (CyHex:EtOAc = 3:1). **^{1}H-NMR:** (400 MHz, DMSO-d_6) δ [ppm] = 1.55 (s, 9 H, C-(CH_3)$_3$), 2.20 (s, 3 H, CH_3), 2.40 (t, ^3J = 7.7 Hz, 2 H, CH_2), 2.43 (s, 3 H, CH_3), 2.97 (t, ^3J = 7.7 Hz, 2 H, CH_2), 6.98 (d, ^3J = 7.3 Hz, 1 H, pyr-C*H*), 7.66 (dd, ^3J = 7.3, 8.4 Hz, 1 H, pyr-C*H*), 8.01 (d, ^3J = 8.4 Hz, 1 H, pyr-C*H*), 10.71 (s, 1 H, N*H*), 12.04 (br s, 1 H), 12.07 (s, 1 H). **^{13}C-NMR:** (151 MHz, MSO-d_6) δ [ppm] = 9.8, 20.2, 23.6, 28.1, 34.3, 80.8, 111.3, 118.7, 122.1, 123.7, 124.9, 130.3, 138.4, 151.6, 156.5, 158.9, 160.4, 174.2. **HR-MS:** (ESI, MeOH): m/z = 410.1680 ($[M+Na]^+$), calcd. 410.1686 (for $[C_{20}H_{25}N_3O_5Na]^+$). **FT-IR:** (ATR): $\tilde{\nu}$ [cm^{-1}] = 3311, 2919, 1704, 1670, 1612, 1571, 1450, 1367, 1319, 1272, 1218, 1159, 1128, 1089, 1000, 923, 890, 844, 800, 779,730, 659, 628, 611. **Melting point:** 195 °C.

3.3 Synthesis of the protected BINAM-ACP Motif 12

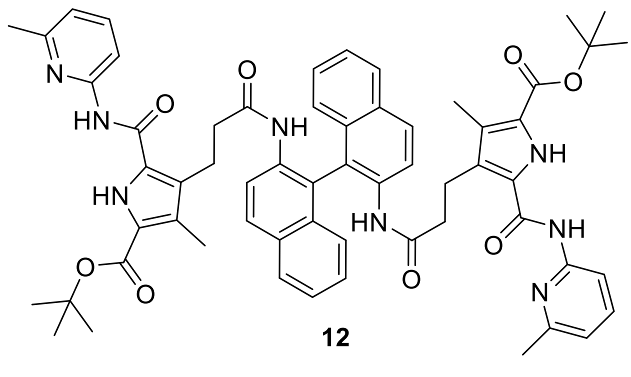

12

The deprotected ACP building block **6** (100 mg, 0.258 mmol, 3 equiv) was dissolved in dry DCM (20 mL) and DMF (0.5 mL). The solution was cooled to 0 °C. At this temperature oxalyl chloride (22.0 µL, 32.7 mg, 0.258 mmol, 3 equiv) was added to give a red solution. The solution was stirred for three hours over which the ice bath was allowed to reach room temperature. Subsequently the solvent was removed in vacuo to give a light red foam, which was dissolved in dry THF (20 mL). This solution was added to a solution of *(S)*-1,1'-binaphthalene-2,2'-diamine (24.5 mg, 0.09 mmol, 1 equiv) and diisopropylethylamine (72.3 µL, 54.9 mg, 0.425 mmol, 5 equiv) in THF (20 mL) at room temperature and was stirred for 16 hours until a yellow suspension formed. The solvent was removed in vacuo and the residue was dissolved in DCM (20 mL). The organic layer was washed with water (3 × 15 mL) and brine (3 × 15 mL), dried over $MgSO_4$, and concentrated under reduced pressure. The residue was purified by flash chromatography (ø = 3 cm, h = 30 cm, CyHex:EtOAc = 2:1) to give **12** (59.2 mg, 58.0 mmol, 67%) as a white solid.

R_f: 0.25 (CyHex:EtOAc = 2:1). **^{1}H-NMR:** (300 MHz, DMSO-d_6) δ [ppm] = 1.55 (s, 18 H, 2 x C-(CH_3)$_3$), 2.00 (s, 6 H, 2 x CH_3), 2.14 - 2.27 (m, 4 H, 2 x CH_2), 2.43 (s, 6 H, 2 x CH_3), 2.65 - 2.84 (m, 4 H, 2 x CH_2), 6.98 (d, ^3J = 7.5 Hz, 2 H, 2 x pyr-C*H*), 7.04 (d, ^3J = 8.8 Hz, 2 H, 2 x pyr-C*H*), 7.19 (d, ^3J = 8.8 Hz, 2 H, 2 x BINOL-*H*), 7.27 (dd, ^3J = 7.5, 8.7 Hz, 2 H, 2 x pyr-C*H*), 7.43 (t, ^3J = 7.5 Hz, 2 H, 2 x BINOL-*H*), 7.67 (t, ^3J = 7.8 Hz, 2 H, 2 x BINOL-*H*), 7.91 (d, ^3J = 8.1 Hz, 2 H, 2 x BINOL-*H*), 8.0 (d, ^3J = 9.4 Hz, 2 H, 2 x BINOL-*H*), 8.2 (d, ^3J = 8.44 Hz, 2 H, 2 x BINOL-*H*), 8.23 (br s, 2 H, 2 x N*H*), 10.63 (br s, 2 H, 2 x N*H*), 11.97 (br s, 2 H, 2 x N*H*). **^{13}C-NMR:** (75 MHz, DMSO-d_6) δ [ppm] = 10.2, 20.8, 23.0, 31.7, 35.2, 87.3, 108.3, 120.5, 122.8, 125.2, 126.8, 127.1, 127.9, 129.1, 130.6, 131.1, 131.9, 132.6, 133.7, 135.9, 137.1, 138.2, 142.1, 156.5, 157.9, 158.7, 161.1, 181.5. **HR-MS:** (ESI, MeOH): m/z = 1023.4752 ([M+H]$^+$), calcd. 1023.4763 (for [$C_{60}H_{63}N_8O_8$]$^+$). **FT-IR:** (ATR): $\tilde{\nu}$ [cm^{-1}] = 3452, 3446, 3087, 2866, 2642, 1778 1649, 1646, 1200, 1071, 1057, 868, 795, 624, 531. **Melting point:** 210 °C.

3.4 Deprotection of the BINAM-ACP Motif 2

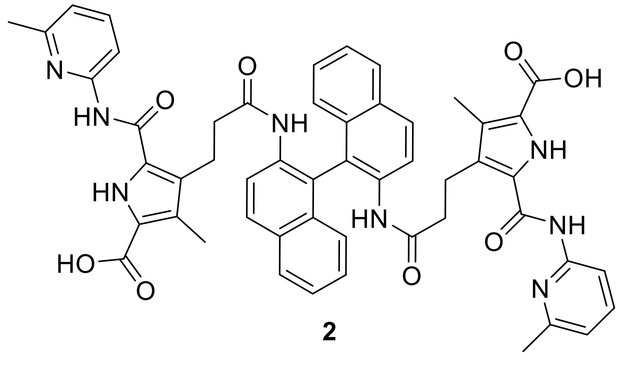

2

(S)-BINAM ACP motif **12** (100 mg, 0.0977 mmol) was dissolved in DCM (5 mL) under ice bath cooling (0 °C). TFA (1 mL) was added and the mixture was stirred for one hour over which the ice bath was allowed to thaw. Subsequently the mixture was stirred for additional four hours at room temperature. The reaction was monitored by TLC. After completion the solvent was removed in vacuo and the residue was resolved in 1 M aqueous hydrochloric acid (10 mL). The resulting solution was dried in vacuo and the crude product was purified by MPLC (ø = 3 cm, h = 60 cm, RP18, MeOH:H_2O = 1:10 to pure MeOH). The pure product **2** (79.0 mg, 0.0867 mmol, 89%) was obtained as a white solid.

R_f: 0.11 (CyHex:EtOAc = 2:1). **^{1}H-NMR:** (300 MHz, DMSO-*d_6*) δ [ppm] = 1.95 (s, 6 H, 2 x CH_3), 2.09 - 2.13 (m, 4 H, 2 x C*H_2*), 2.41 (s, 6 H, 2 x C*H_3*), 2.53 - 2.66 (m, 4 H, 2 x C*H_2*), 6.90 (d, ^3J = 9.1 Hz, 2 H, 2 x pyr-C*H*), 6.96 (d, ^3J = 7.2 Hz, 2 H, 2 x pyr-C*H*), 7.23 (dd, ^3J = 9.1, 7.2, 2 H, 2 x pyr-C*H*), 7.41 (t, ^3J = 8.1, 2 H, 2 x BINOL-*H*), 7.63 (t, ^3J = 8.1, 2 H, 2 x BINOL-*H*), 7.81 (d, ^3J = 9.1 Hz, 2 H, 2 x BINOL-*H*), 7.87 (d, ^3J = 8.1 Hz, 2 H, 2 x BINOL-*H*), 7.95 (d, ^3J = 8.8 Hz, 2 H, 2 x BINOL-*H*), 8.01 (d, ^3J = 8.8 Hz, 2 H, 2 x BINOL-*H*), 8.78 (br s, 2 H, 2 x N*H*), 10.66 (br s, 2 H, 2 x O*H*), 12.09 (br s, 2 H, 2 x N*H*). **^{13}C-NMR:** (75 MHz, DMSO-*d_6*) δ [ppm] = 10.3, 22.0, 22.5, 37.2, 89.5, 111.2, 123.2, 124.1, 125.6, 126.3, 126.9, 127.4, 127.9, 128.8, 129.4, 131.8, 133.2, 135.1, 137.6, 139.7, 141.1, 154.6, 157.2, 159.5, 162.4, 182.5. **HR-MS:** (ESI, MeOH): m/z = 933.3354 ($[M+Na]^+$), calcd. 933.3331 (for $[C_{52}H_{46}N_8O_8Na]^+$). **FT-IR:** (ATR): $\tilde{\nu}$ [cm^{-1}] = 3551, 3522, 3023, 2866, 2474, 1729, 1645, 1635, 1604, 1085, 1052, 1050, 896, 757, 676, 648. **Elemental analysis:** calcd. (%) for $C_{52}H_{46}N_8O_8$: C 68.56, H 5.09, N 12.30; found: C 68.40, H 4.77, N 8.55. **Melting point:** Decomposition at 265 °C.

4. Synthesis of the amino acid extended BINAM-ACP 3

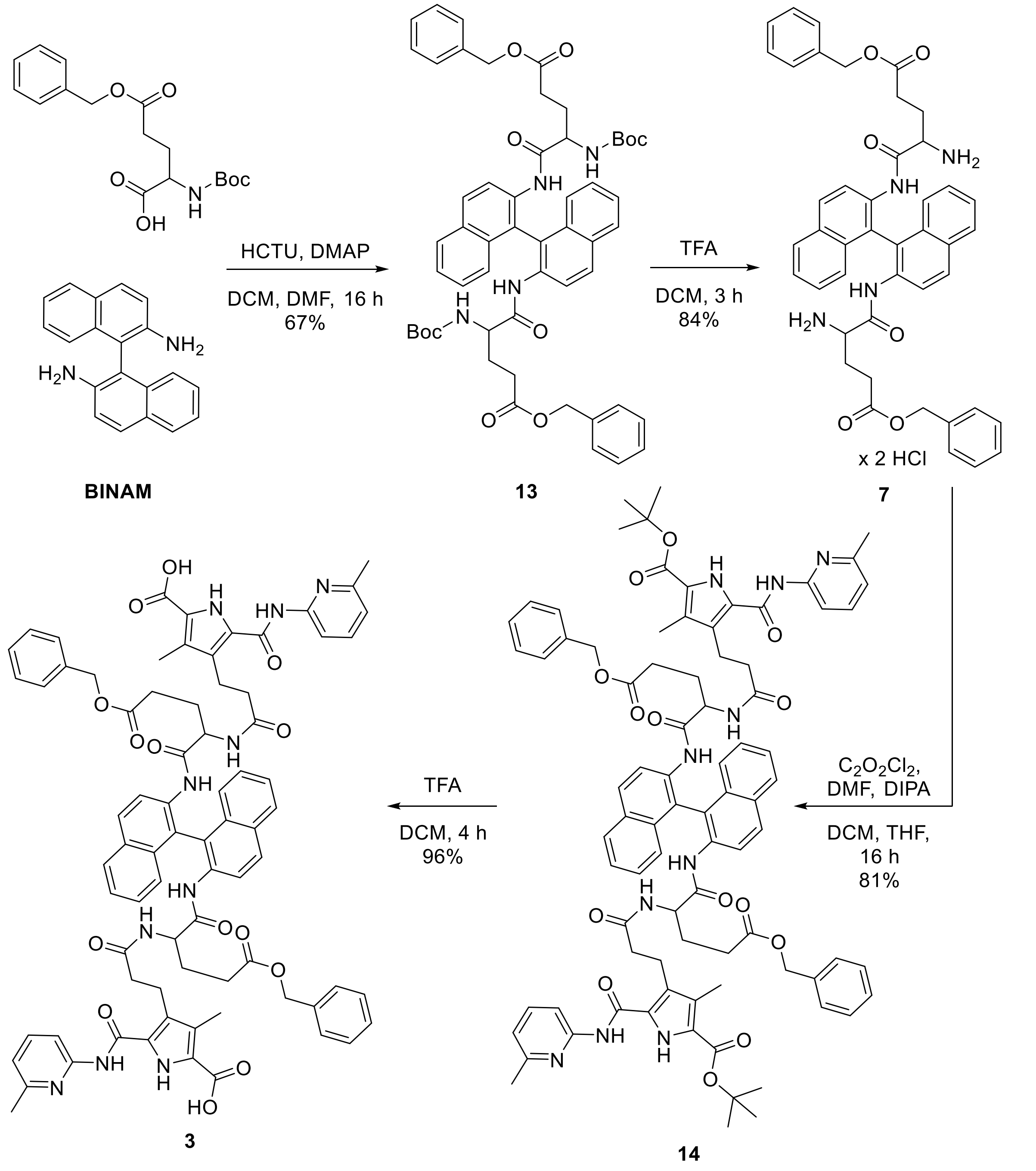

Scheme S3: Synthetic scheme of amino acid extended BINAM-ACP (3).

4.1 Synthesis of BINAM-glutamic acid 13

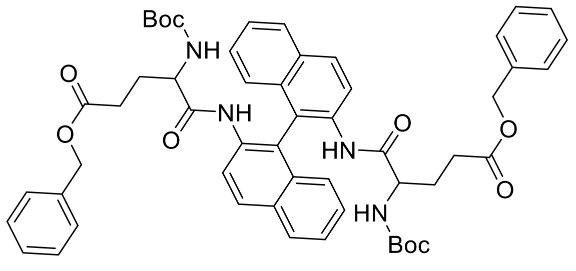

13

L-Boc-glutamic acid benzyl ester (7.11 g, 21.1 mmol, 3 equiv), HCTU (8.71 g, 21.1 mmol, 3 equiv) and DMAP (4.30 g, 35.1 mmol, 5 equiv) were dissolved in DCM (100 mL) and DMF (1 mL) and stirred for one hour. Subsequently *(S)*-BINAM (2.00 g, 7.02 mmol, 1 equiv) was added and the reaction mixture was stirred for 16 hours at room temperature. The solvent was evaporated in vacuo and the residue was redissolved in EtOAc. The organic layer was washed with water (3 × 50 mL) and brine (3 × 50 mL) and dried over $MgSO_4$. The solvent was evaporated and the crude product purified by flash chromatography (ø = 6 cm, h = 50 cm, CyHex:EtOAc = 5:1) to obtain the product **13** (4.35 g, 4.71 mmol, 67%) as a white foam.

R_f: 0.52 (CyHex:EtOAc = 5:1). **^{1}H-NMR:** (300 MHz, DMSO-d_6) δ [ppm] = 1.27 - 1.37 (m, 22 H, 2 x C-(CH_3)$_3$ + 2 x CH_2), 1.85 - 1.92 (m, 4 H, 2 x CH_2), 3.76 - 3.84 (m, 2 H, 2 x C*H*), 5.01 (s, 4 H, 2 x CH_2), 6.75 (d, ^3J = 7.82 Hz, 2 H, 2 x BINOL-*H*), 6.85 (d, ^3J = 8.44 Hz, 2 H, 2 x BINOL-*H*), 7.20 (d, ^3J = 7.50 Hz, 2 H, 2 x BINOL-*H*), 7.32 - 7.38 (m, 12 H, 2 x BINOL-*H* + 2 x C_6H_5), 7.89 (d, 7.82 Hz, 2 H, 2 x BINOL-*H*), 7.92 (br s, 2 H, 2 x N*H*), 7.99 (d, ^3J = 9.07 Hz, 2 H, 2 x BINOL-*H*), 8.90 (br s, 2 H, 2 x N*H*). **^{13}C-NMR:** (75 MHz, DMSO-d_6) δ [ppm] = 17.8, 30.3, 34.3, 54.8, 64.1, 81.5, 120.5, 122.5, 125.0, 125.8, 127.2, 128.9, 129.2, 132.3, 133.5, 136.9, 138.9, 140.7, 142.5, 149.3, 160.7, 176.0, 180.9. **HR-MS:** (ESI, MeOH): m/z = 923.4187 ($[M+H]^+$), calcd. 923.4226 (for $[C_{54}H_{59}N_4O_{10}]^+$). **FT-IR:** (ATR): $\tilde{\nu}$ [cm^{-1}] = 2979, 1675, 1596, 1496, 1452, 1365, 1247, 1160, 1052, 865, 811, 746, 696. **Melting point:** 67 °C.

4.2 Deprotection of BINAM-Glutamic acid 7

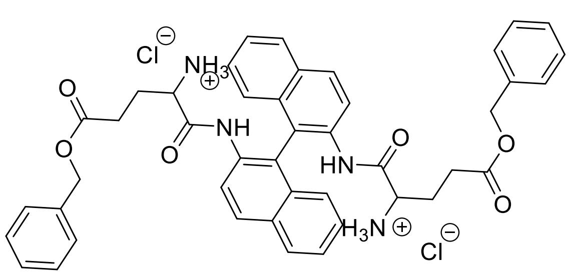

7

The *(S)*-BINAM glutamic acid **13** (200 mg, 0.217 mmol) was dissolved in DCM (6 mL) and TFA (3 mL) was added to initiate deprotection. The mixture was stirred for three hours at room temperature and the reaction progress was monitored by TLC. The solvent was removed in vacuo and the residue was dissolved in 1 M aqueous hydrochloric acid (10 mL) and dried in vacuo again. The crude product was purified by MPLC (ø = 2 cm, h = 30 cm, RP18, MeOH:H_2O = 1:10 to pure MeOH). Hydrochloric acid (1 M in H_2O, 10 mL) was added and the resulting solution was dried in vacuo. The hydrochloride **7** (145 mg, 0.182 mmol, 84%) was obtained as a white solid.

R$_f$: 0.05 (CyHex:EtOAc = 4:1). **^{1}H-NMR:** (300 MHz, DMSO-*d$_6$*) δ [ppm] = 1.25 - 1.32 (m, 4 H, 2 x C*H$_2$*), 1.42 - 1.70 (m, 4 H, 2 x C*H$_2$*), 3.77 - 3.82 (m, 2 H, 2 x C*H*), 5.05 (s, 4 H, 2 x C*H$_2$*), 6.94 (d, ^3J = 8.44 Hz, 2 H, 2 x BINOL-*H*), 7.19 (t, ^3J = 7.19 Hz, 2 H, 2 x BINOL-*H*), 7.31 - 7.48 (m, 12 H, 2 x BINOL-*H* + 2 x C$_6$*H$_5$*), 7.68 (d, ^3J = 9.07 Hz, 2 H, 2 x BINOL-*H*), 7.86 (d, ^3J = 8.13 Hz, 2 H, 2 x BINOL-*H*), 7.96 (d, ^3J = 9.07 Hz, 2 H, 2 x BINOL-*H*), 8.14 (br s, 4 H, 2 x N*H$_2$*), 9.63 (s, 2 H, 2 x N*H*). **^{13}C-NMR:** (75 MHz, DMSO-*d$_6$*) δ [ppm] = 18.5, 36.7, 56.3, 66.7, 120.1, 121.6, 122.7, 123.2, 125.6, 126.7, 128.2, 132.4, 134.0, 135.4, 137.9, 140.5, 142.4, 145.9, 165.8, 169.3. **HR-MS:** (ESI, MeOH): m/z = 723.3168 ([M+H$^+$-2HCl]$^+$), calcd. 723.3177 (for [$C_{44}H_{43}N_4O_6$]$^+$). **FT-IR:** (ATR): $\tilde{\nu}$ [cm^{-1}] = 2983, 1775, 1664, 1597, 1489, 1484, synthe1451, 1356, 1277, 1155, 1048, 852, 801, 702, 680. **Melting point:** 98 °C.

4.3 Synthesis of the BINAM-glutamic acid-ACP motif 14

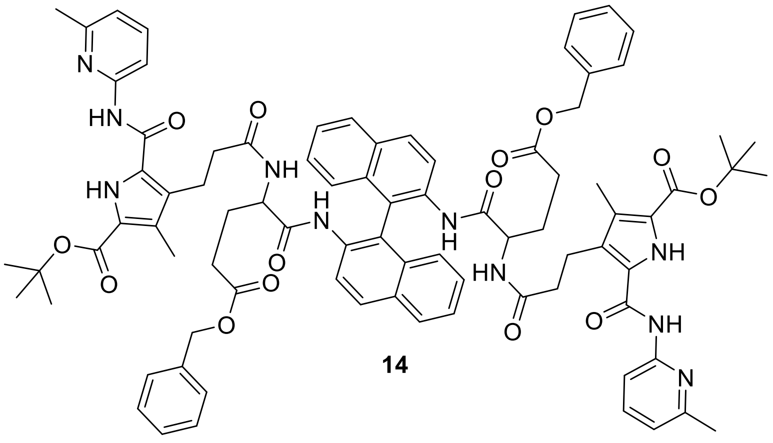

14

A solution of building block **6** (100 mg, 0.258 mmol, 3 equiv) in dry DCM (20 mL) and DMF (0.5 mL) was cooled to 0 °C. At this temperature oxalyl chloride (22.0 µL, 32.7 mg, 0.258 mmol, 3 equiv) was added, which resulted in a yellow solution. The solution was stirred for 16 hours over which the ice bath was allowed to reach room temperature. Subsequently the solvents were removed in vacuo, yielding a yellow foam which was dissolved in dry THF (20 mL). This solution was added to a solution of the deprotected *(S)*-BINAM glutamic acid **7** (62.2 mg, 0.0860 mmol, 1 equiv) and diisopropylethylamine (72.0 µL, 54.7 mg, 0.423 mmol, 5 equiv) in THF (20 mL) at room temperature. The reaction mixture was stirred for 16 hours until a suspension had formed. The solvent was removed in vacuo and the residue was dissolved in DCM (20 mL). The organic layer was washed with water (3 × 15 mL) and brine (3 × 15 mL) and dried over $MgSO_4$. The solvents were removed in vacuo and the residue was purified by column chromatography (ø = 3 cm, h = 30 cm, CyHex:EtOAc = 4:1) to give pure **14** (102 mg, 0.0698 mmol, 81%) as a white solid.

R$_f$: 0.46 (CyHex:EtOAc = 2:1). **^{1}H-NMR:** (300 MHz, $CDCl_3$) δ [ppm] = 1.56 (s, 18 H, 2 x C-(C*H$_3$*)$_3$), 2.24 (s, 6 H, 2 x C*H$_3$*), 2.52 (s, 6 H, 2 x C*H$_3$*), 2.67 - 2.78, (m, 8 H, 4 x C*H$_2$*), 3.15 (t, ^3J = 7.3 Hz, 4 H, 2 x C*H$_2$*), 3.27 (t, ^3J = 7.2 Hz, 4 H, 2 x C*H$_2$*), 3.80 - 3.81 (m, 2 H, 2 x C*H*), 5.08 (s, 4 H, 2 x C*H$_2$*), 6.86 (d, ^3J = 7.5 Hz, 2 H, 2 x pyr-C*H*), 6.96 (d, ^3J = 2.8 Hz, 2 H, 2 x pyr-C*H*), 7.07 (d, ^3J = 3.8 Hz, 2 H, 2 x BINOL-*H*), 7.27 - 7.30 (m, 14 H, 2 x BINOL-*H* + 2 x C$_6$*H$_5$* + 2 x pyr-C*H*), 7.43 (d, ^3J = 3.5 Hz, 2 H, 2 x BINOL-*H*), 7.53 (t, ^3J = 6.3 Hz, 2 H, 2 x BINOL-*H*), 7.69 (d, ^3J = 3.3, 2 H, 2 x BINOL-*H*), 8.07 (d, ^3J = 8.1 Hz, 2 H, 2 x BINOL-*H*). **^{13}C-NMR:** (75 MHz, $CDCl_3$) δ [ppm] = 26.2, 27.2, 30.3, 30.8, 31.3, 36.8, 43.1, 62.7, 66.9, 70.7, 94.6, 104.9, 109.8, 112.7, 113.8, 119.4, 121.3, 123.8, 127.0, 131.0, 131.8,

132.7, 134.7, 137.0, 138.0, 138.7, 140.7, 141.7, 142.1, 142.8, 147.2, 149.8, 152.5, 156.1, 157.1, 167.8, 171.4, 174.1. **HR-MS:** (ESI, DCM): m/z = 1461.6493 ($[M+H]^+$), calcd. 1461.6554 (for $[C_{84}H_{89}N_{10}O_{14}]^+$). **FT-IR: (**ATR): $\tilde{\nu}$ [cm^{-1}] = 3437, 3266, 3058, 2925, 2710, 1798, 1675, 1666, 1634, 1158, 1058, 1030, 1012, 821, 801, 765, 653, 641. **Melting point:** Decomposition at 254 °C.

4.4 Deprotection of the BINAM-glutamic acid-ACP motif 3

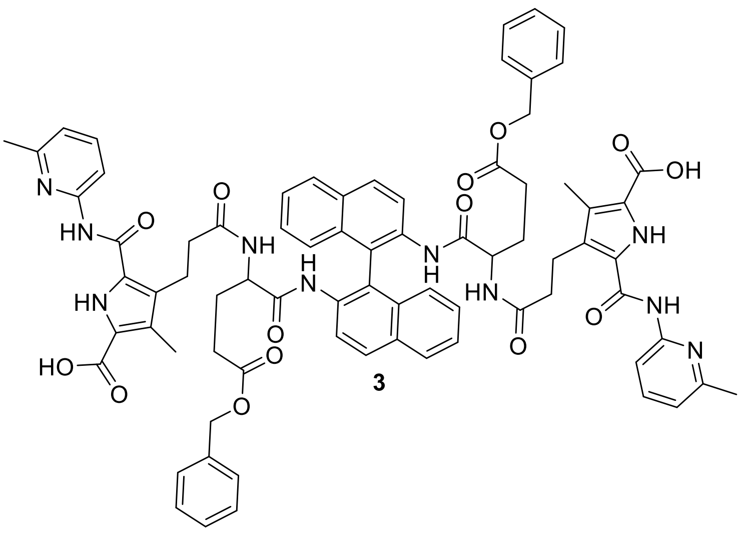

3

The *(S)*-BINAM glutamic acid ACP motif **14** (100 mg, 0.0684 mmol) was dissolved in DCM (5 mL) and TFA (1 mL) was added to initiate deprotection. The mixture was stirred for 16 hours and the reaction progress was monitored by TLC. The solvent was removed in vacuo, and the residue was taken up in 1 M aqueious hydrochloric acid (10 mL), dried in vacuo and the crude product was purified by MPLC (ø = 2 cm, h = 30 cm, RP18, MeOH:H_2O = 1:10 to pure MeOH). The deprotected product **3** (88.5 mg, 65.6 µmol, 96%) was obtained as a white solid.

R_f: 0.21 (CyHex:EtOAc = 4:1). **^{1}H-NMR:** (300 MHz, $CDCl_3$) δ [ppm] = 2.04 (s, 6 H, 2 x CH_3), 2.27, (s, 4 H, 2 x CH_2), 2.30 - 2.33 (m, 4 H, 2 x CH_2), 2.36 - 2.38 (m, 4 H, 2 x CH_2), 2.69 - 2.71 (m, 4 H, 2 x CH_2), 2.85 - 2.88 (m, 4 H, 2 x CH_2), 4.27 - 4.29 (m, 2 H, 2 x C*H*), 4.97 (s, 4 H, 2 x CH_2), 6.98 (d, ^3J = 3.1 Hz, 2 H, pyr-C*H*), 7.12 (d, ^3J = 2.8 Hz, 2 H, pyr-C*H*), 7.17 (d, ^3J = 5.6 Hz, 2 H, 2 x BINOL-*H*), 7.32 (m, 14 H, , 2 x BINOL-*H* + 2 x C_6H_5 + 2 x pyr-C*H*), 7.45 (d, ^3J = 4.7 Hz, 2 H, 2 x BINOL-*H*), 7.55 (t, ^3J = 3.1 Hz, 2 H, 2 x BINOL-*H*), 7.66 (d, ^3J = 5.6 Hz, 2 H, 2 x BINOL-*H*), 7.87 (d, ^3J = 3.1 Hz, 2 H, 2 x BINOL-*H*). **^{13}C-NMR:** (75 MHz, $CDCl_3$) δ [ppm] = 25.9, 29.4, 30.0, 30.5, 36.6, 42.8, 62.4, 66.6, 72.2, 94.9, 104.6, 109.5, 110.2, 113.5, 119.2, 121.1, 130.2, 130.7, 131.5, 132.4, 134.4, 136.7, 137.7, 138.4, 140.4, 141.4, 141.8, 142.5, 149.5, 150.8, 152.3, 154.0, 156.8, 167.6, 168.8, 173.8. **HR-MS:** (ESI, MeOH): m/z = 1371.5078 ($[M+Na]^+$), calcd. 1371.5122 (for $[C_{76}H_{72}N_{10}O_{14}Na]^+$). **FT-IR: (**ATR): $\tilde{\nu}$ [cm^{-1}] 3415, 3030, 2901, 2891, 1847, 1672, 1621, 1071, 1100, 922, 859, 821, 794, 633. **Elemental analysis:** calcd. (%) for $C_{76}H_{72}N_{10}O_{14}$: C 67.64, H 5.38, N 10.38; found: C 67.2, H 4.60, N 8.76. **Melting point:** Decomposition at 219 °C.

5. Synthesis of the substituted BINAM-GCP motif 4

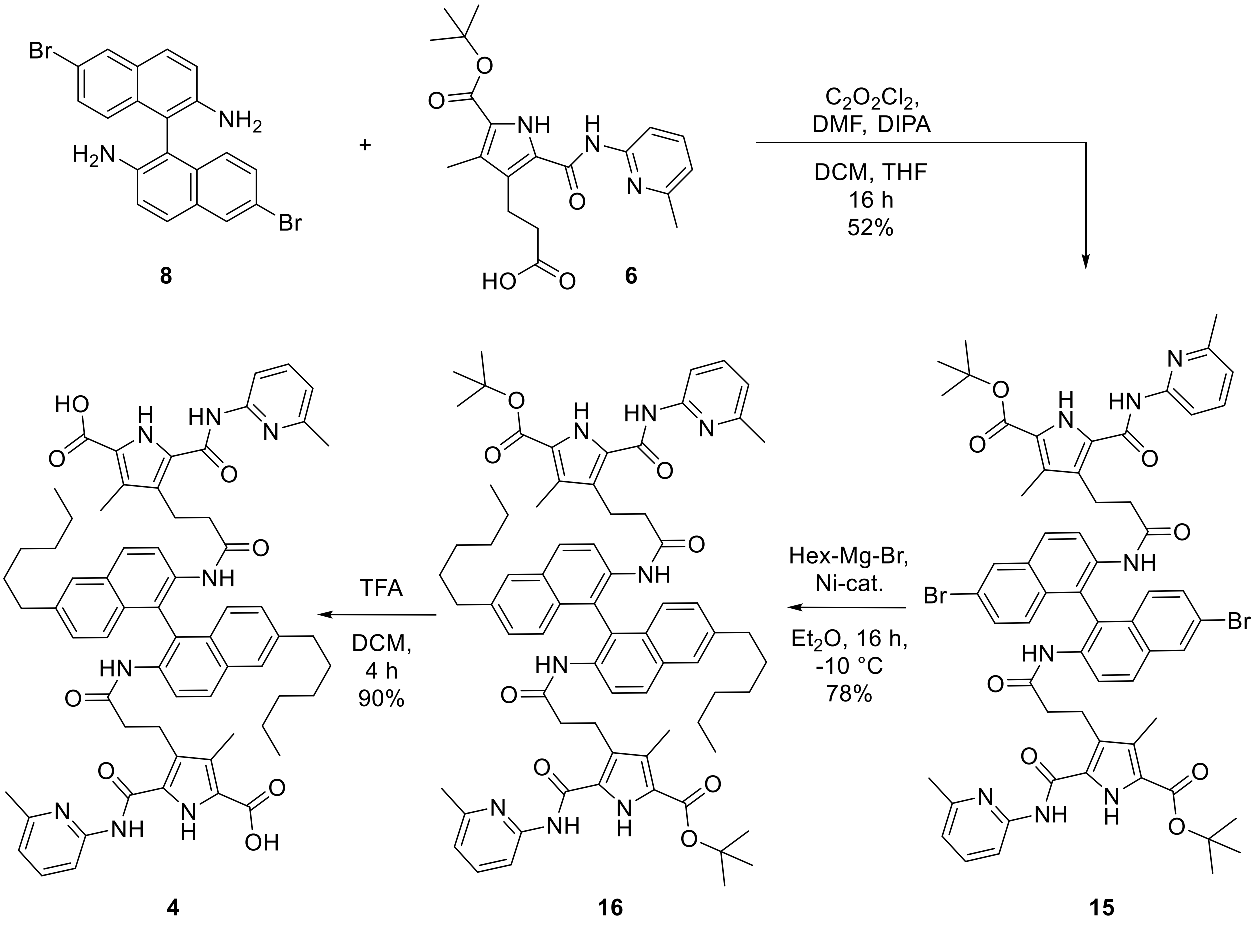

Scheme S4: Synthetic scheme of 4

5.1 Synthesis of BINAM-Br-ACP 15

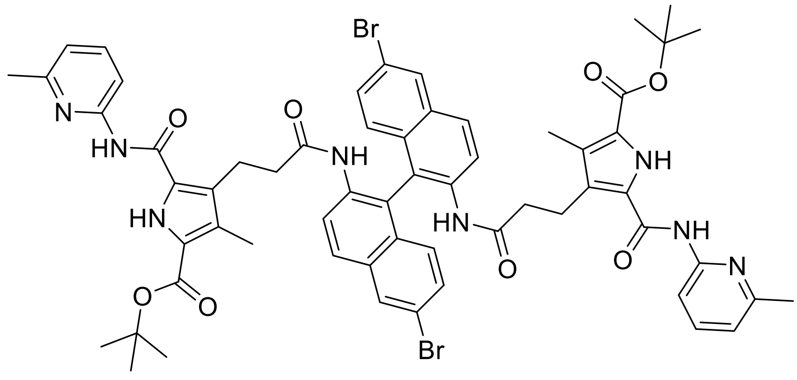

15

A dry schlenk tube was filled with a solution of the deprotected ACP building block **6** (26.0 mg, 0.0671 mmol, 3 equiv) in dry DCM (10 mL). DMF (0.1 mL) was added and the solution was cooled to 0 °C. At this temperature oxalyl chloride (6.0 µL, 8.52 mg, 0.0671 mmol, 3 equiv) was added yielding a yellow solution. The solution was stirred for 16 hours over which the ice bath was allowed to thaw. Subsequently the solvents were removed in vacuo yielding a yellow foam, which was dissolved in dry THF (10 mL). At room temperature this solution was added to a solution of

the brominated *(S)*-BINAM **8**[2], (9.89 mg, 0.0224 mmol, 1 equiv) and diisopropylethylamine (18.8 µL, 14.3 mg, 0.111 mmol, 5 equiv) in THF (20 mL). After stirring for 16 hours at the same temperature a suspension had formed. The solvents were removed under reduced pressure and the residue was dissolved in DCM (20 mL). The organic layer was washed with water (3 × 15 mL) and brine (3 × 15 mL) and dried over $MgSO_4$. The product was purified by flash chromatography (ø = 3 cm, h = 30 cm, CyHex:EtOAc = 10:1) yielding **15** (13.8 mg, 0.0117 mmol, 52%) as a white solid.

R_f: 0.56 (CyHex:EtOAc = 3:1). **^{1}H-NMR:** (300 MHz, DMSO-d_6) δ [ppm] = 1.56 (s, 18 H, 2 x C-(C*H*$_3$)$_3$), 1.98 (s, 6 H, 2 x C*H*$_3$), 2.22 (t, ^3J = 6.7 Hz, 4 H, 2 x C*H*$_2$), 2.41 (s, 6 H, 2 x C*H*$_3$), 2.76 (t, ^3J = 6.6 Hz, 4 H, 2 x C*H*$_2$), 6.7 (d, ^3J = 9.1 Hz, 2 H, 2 x pyr-C*H*), 6.99 (d, ^3J = 8.4 Hz, 2 H, 2 x pyr-C*H*), 7.22 (dd, ^3J = 8.4 Hz, 9.2 Hz, 2 H, 2 x pyr-C*H*), 7.23 (d, ^3J = 8.8 Hz, 2 H, 2 x BINOL-*H*), 7.66 (t, ^3J = 6.0 Hz, 2 H, 2 x BINOL-*H*), 7.74 (d, ^3J = 9.1 Hz, 2 H, 2 x BINOL-*H*), 7.98 (d, ^3J = 1.9 Hz, 2 H, 2 x BINOL-*H*), 8.02 (d, ^3J = 6.0 Hz, 2 H, 2 x BINOL-*H*), 8.20 (br s, 2 H, 2 x N*H*), 10.59 (br s, 2 H, 2 x N*H*), 11.93 (br s, 2 H, 2 x N*H*). **^{13}C-NMR:** (151 MHz, $CDCl_3$) δ [ppm] =12.2, 19.3, 23.1, 30.3, 35.2, 88.1, 114.5, 118.6, 120.2, 121.6, 123.3, 123.8, 125.4, 126.8, 127.1, 128.2, 128.7, 129.7, 129.9, 130.7, 132.3, 133.2, 133.9, 135.9, 149.3, 153.5, 162.8, 175.9. **HR-MS:** (ESI, MeOH): m/z = 1181.2826 ([M+H]$^+$), calcd. 1181.2953 (for [$C_{60}H_{61}Br_2N_8O_8$]$^+$). **FT-IR: (**ATR): $\tilde{\nu}$ [cm^{-1}] = 3403, 3321, 3061, 2876, 2464, 1843, 1796, 1663, 1629, 1130, 1108, 1034, 864, 753, 585, 572. **Melting point:** 204 °C.

5.2 Synthesis of Hexyl-BINAM-ACP 16

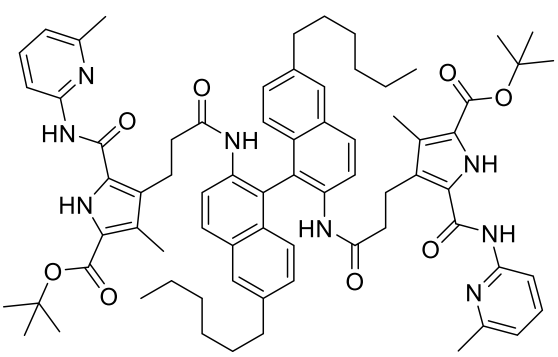

16

The brominated product **15** (20.0 mg, 0.0169 mmol, 1 equiv) and 1,3-bis(diphenyl phosphino)propane nickel(II) chloride (0.220 mg, 0.406 µmol, 2.4 mol %) were dissolved in dry Et_2O (20 mL). The mixture was cooled to −10 °C in an ice/NaCl bath. Then a 0.8 M solution of hexyl magnesium bromide in Et_2O (40.0 µL, 0.034 mmol, 2 equiv) was added at this temperature. The cooling bath was allowed to thaw while stirring was continued for 16 hours. The catalyst was filtered of and the organic layer was washed with 0.5 M HCl (2 × 10 mL), water (3 × 15 mL) and brine (3 × 15 mL). After the removal of the solvents under reduced pressure, the crude product was purified by flash chromatography (ø = 3 cm, h = 30 cm, CyHex:EtOAc = 7:1). The pure product **16** (15.8 mg, 0.0133 mmol, 78%) was obtained as a white solid.

R_f: 0.68 (CyHex:EtOAc = 4:1). **^{1}H-NMR:** (400 MHz, $CDCl_3$) δ [ppm] = 0.91 (t, ^3J = 6.88 Hz, 6 H, 2 x C*H*$_3$), 1.24 - 1.46 (m, 12 H, 2 x C*H*$_2$-C*H*$_2$-$_2$), 1.28 - 1.56 (m, 24 H, 2 x C-(C*H*$_3$)$_3$ + 2 x C*H*$_3$), 2.30 (s, 6 H, 2 x C*H*$_3$), 2.56 (s, 4 H, 2 x C*H*$_2$), 2.61 - 2.67 (m, 8 H, 4 x C*H*$_2$), 2.79 (t, ^3J = 7.5 Hz, 4 H, 2 x C*H*$_2$), 6.89 (d, ^3J = 9.1 Hz, 2 H, pyr-C*H*), 7.02 (d, ^3J = 6.0 Hz, 2 H, 2 x pyr-C*H*), 7.15 (d, ^3J = 8.8 Hz, 2 H, 2 x

[2] A. Sakakura, K. Suzuki and K. Ishihara, *Adv. Synth. Catal.* **2006**, *348*, 2457-2465.

BINOL-*H*), 7.26 (dd, ^3J = 6.1 Hz, 9.1 Hz, 2 H, 2 x pyr-C*H*), 7.64 (t, ^3J = 7.8 Hz, 2 H, 2 x BINOL-*H*), 7.7 (d, ^3J = 8.76 Hz, 2 H, 2 x BINOL-*H*), 7.9 (d, ^3J = 2.19 Hz, 2 H, 2 x BINOL-*H*), 8.1 (d, ^3J = 6.88 Hz, 2 H, 2 x BINOL-*H*). **^{13}C-NMR:** (151 MHz, $CDCl_3$) δ [ppm] = 12.5, 13.9, 22.5, 22.9, 29.2, 30.2, 30.7, 30.8, 31.7, 35.2, 36.1, 81.4, 104.2, 115.5, 117.8, 119.6, 124.1, 125.7, 127.4, 128.5, 129.3, 130.2, 132.1, 132.9, 134.3, 135.3, 137.1, 137.5, 141.4, 149.7, 155.5, 160.0, 160.7, 174.5. **HR-MS:** (ESI, DCM): m/z = 1191.6701 ([M+H]$^+$), calcd. 1191.6641 (for [$C_{72}H_{87}N_8O_8$]$^+$). **FT-IR: (**ATR): $\tilde{\nu}$ [cm^{-1}] = 3462, 3358, 3070, 2872, 2684, 1821, 1742, 1633, 1630, 1172, 1134, 1040, 944, 765, 748, 550. **Melting point:** 255 °C.

5.3 Deprotection of hexyl-BINAM-ACP 4

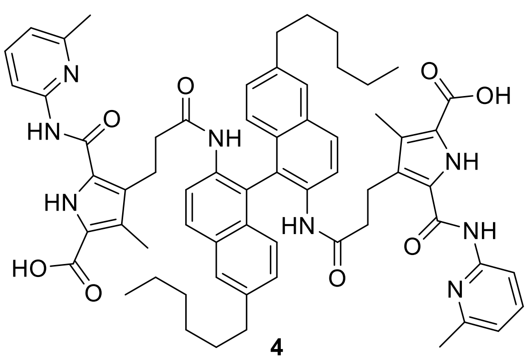

4

The *(S)*-BINAM hexyl ACP motif **16** (12 mg, 0.0101 mmol) was dissolved in DCM (5 mL) and TFA (1 mL) was added to initiate deprotection. The mixture was stirred for 16 hours and the reaction progress was monitored by TLC. The solvent was removed *in vacuo* and the crude product was purified by MPLC (ø = 2 cm, h = 30 cm, RP18, MeOH:H_2O = 1:10 to pure MeOH). The deprotected product **4** (9.80 mg, 0.00908 mmol, 90%) was obtained as a white solid.

R_f: 0.33 (CyHex:EtOAc = 4:1). **^{1}H-NMR:** (400 MHz, $CDCl_3$) δ [ppm] = 0.88 (t, ^3J = 7.97 Hz, 6 H, 2 x C*H*$_3$), 1.18 - 1.36 (m, 12 H, 2 x CH_2-C*H*$_2$-C*H*$_2$), 1.52 (m, 4 H, 2 x C*H*$_2$), 1.55 (m, 4 H, 2 x C*H*$_2$), 2.05 (s, 6 H, 2 x C*H*$_3$), 2.16 (m, 4 H, 2 x C*H*$_2$), 2.46 (s, 6 H, 2 x C*H*$_3$), 2.78 (m, 4 H, 2 x C*H*$_2$), 6.87 (m, 4 H, 2 x C*H*$_2$), 6.90 (m, 2 H, 2 x BINOL-*H* + 2 x pyr-C*H*), 7.15 (d, ^3J = 9.1 Hz, 2 H, 2 x pyr-C*H*), 7.23 (d, ^3J = 6.7 Hz, 2 H, 2 x BINOL-*H*), 7.28 (d, ^3J = 2.2 Hz, 2 H, 2 x BINOL-*H*), 7.62 (dd, ^3J = 7.5 Hz, 9.0 Hz, 2 H, pyr-C*H*), 7.71 (d, ^3J = 8.8 Hz, 2 H, 2 x BINOL-*H*), 7.93 (d, ^3J = 2.2 Hz, 2 H, 2 x BINOL-*H*). **^{13}C-NMR:** (151 MHz, $CDCl_3$) δ [ppm] = 12.0, 14.1, 21.4, 21.9, 28.6, 29.3. 30.2, 32.7, 34.6, 37.2, 109.6, 114.5, 117.2, 119.7, 123.5, 126.1, 126.8, 127.2, 128.7, 130.4, 131.6, 132.9, 133.7, 135.3, 136.5, 138.4, 140.8, 146.5, 154.9, 164.4, 166.0, 173.2. **HR-MS:** (ESI, DCM): m/z = 1101.5299 ([M+Na]$^+$), calcd. 1101.5209 (for [$C_{64}H_{70}N_8O_8Na$]$^+$). **FT-IR: (**ATR): $\tilde{\nu}$ [cm^{-1}] = 3456, 3121, 3021, 2925, 1756, 1658, 1655, 1635, 1143, 1058, 1066, 955, 791, 764. **Elemental analysis:** calcd. (%) for $C_{64}H_{70}N_8O_8$: C 71.22, H 6.54, N 10.38; found: C 69.40, H 6.02, N 10.60. **Melting point:** Decomposition at 278 °C.

6. Viscosity measurements

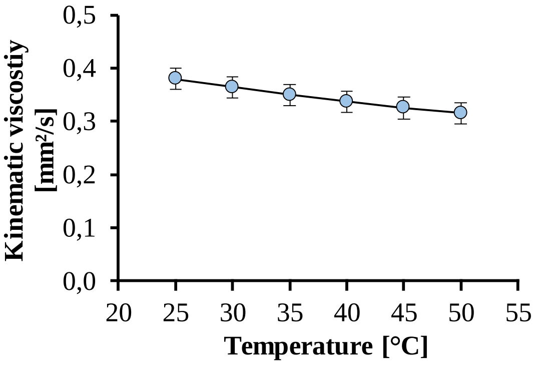

Figure S1: Kinematic viscosity of pure chloroform.

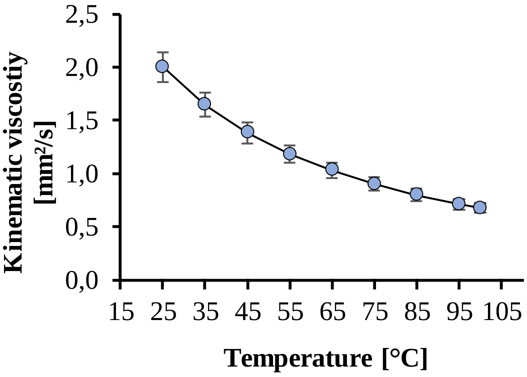

Figure S2: Kinematic viscosity of pure DMSO.

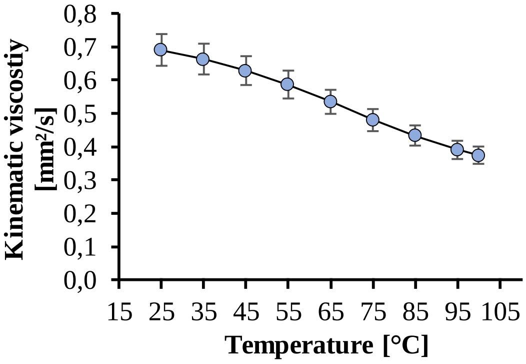

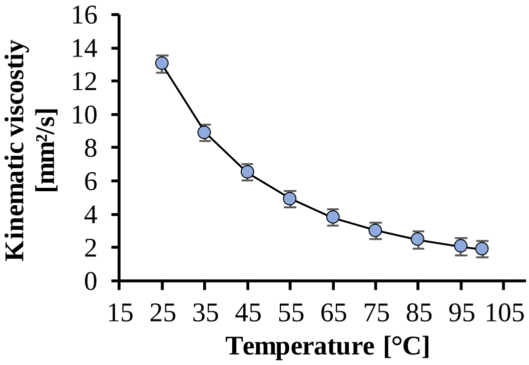

Figure S3: Kinematic viscosity of pure toluene.

Figure S4: Kinematic viscosity of pure Nynas NS8.

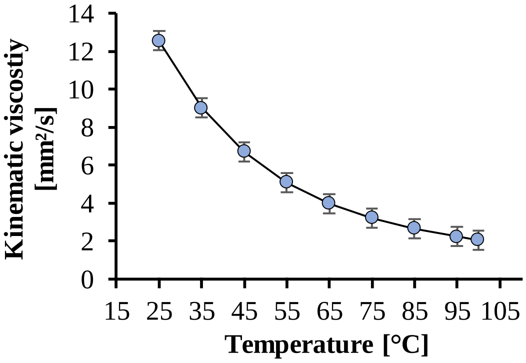

Figure S5: Kinematic viscosity of Nexbase 3020.

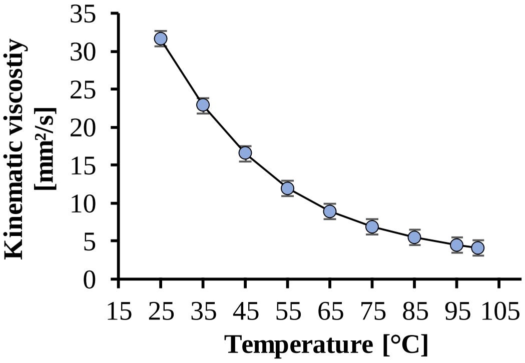

Figure S6: Kinematic viscosity of Nexbase 3043.

6.1 Viscosity measurements of 1 in DMSO

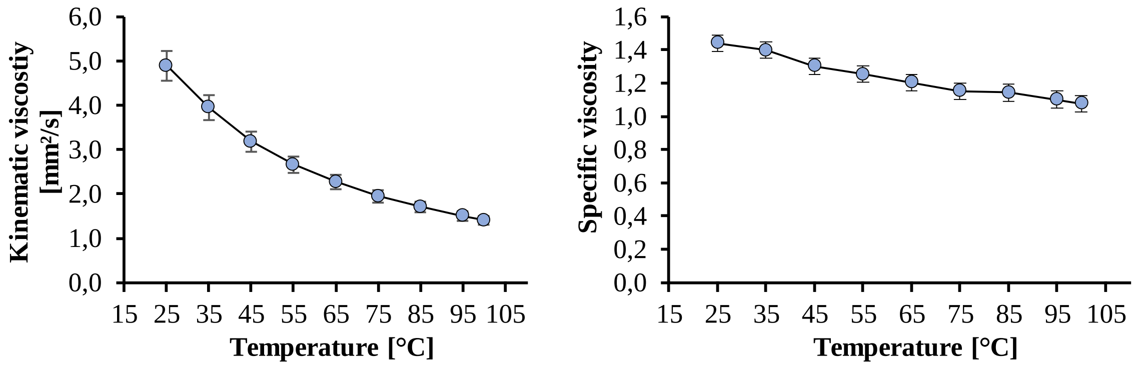

Figure S7: Kinematic (left) and specific (right) viscosity for 1 in DMSO at 200 mM.

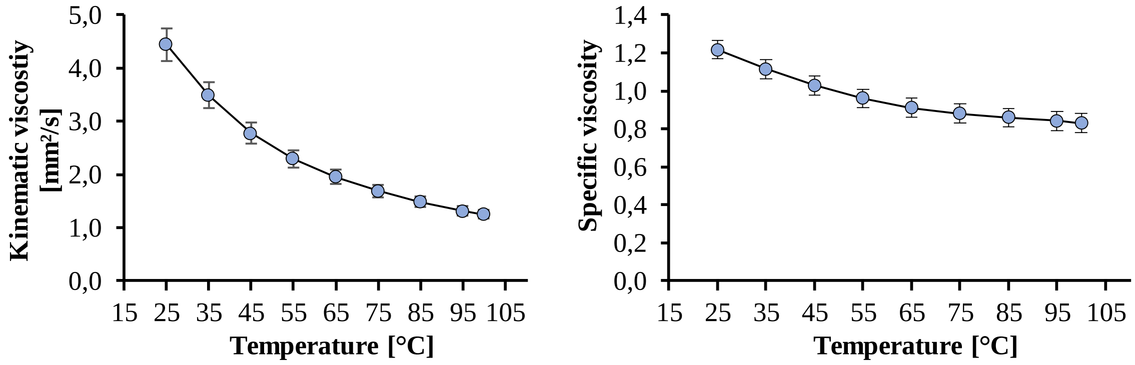

Figure S8: Kinematic (left) and specific (right) viscosity for 1 in DMSO at 180 mM.

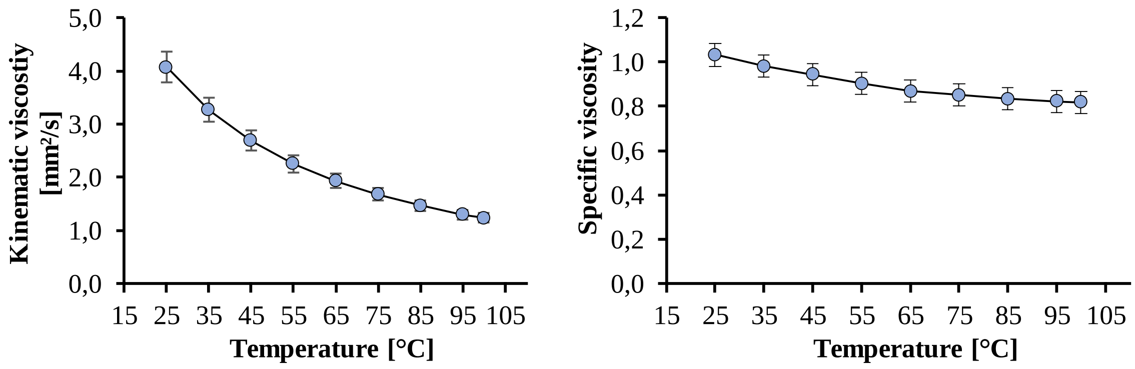

Figure S9: Kinematic (left) and specific (right) viscosity for 1 in DMSO at 160 mM.

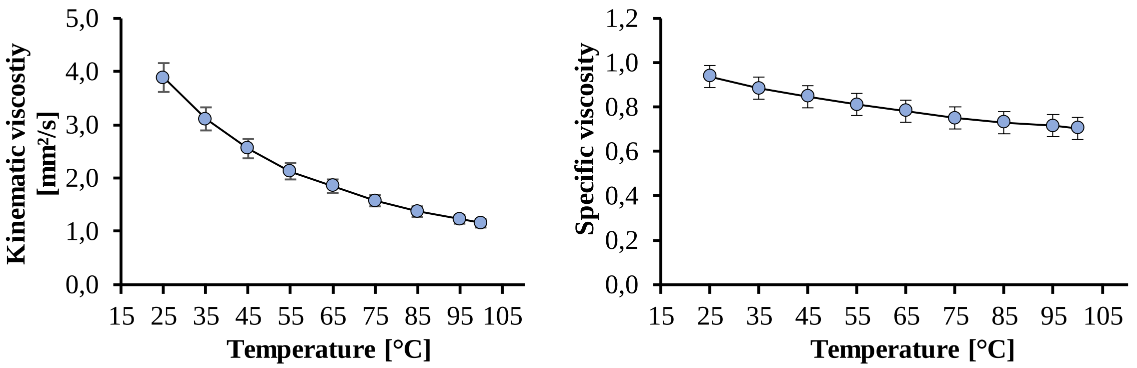

Figure S10: Kinematic (left) and specific (right) viscosity for 1 in DMSO at 140 mM.

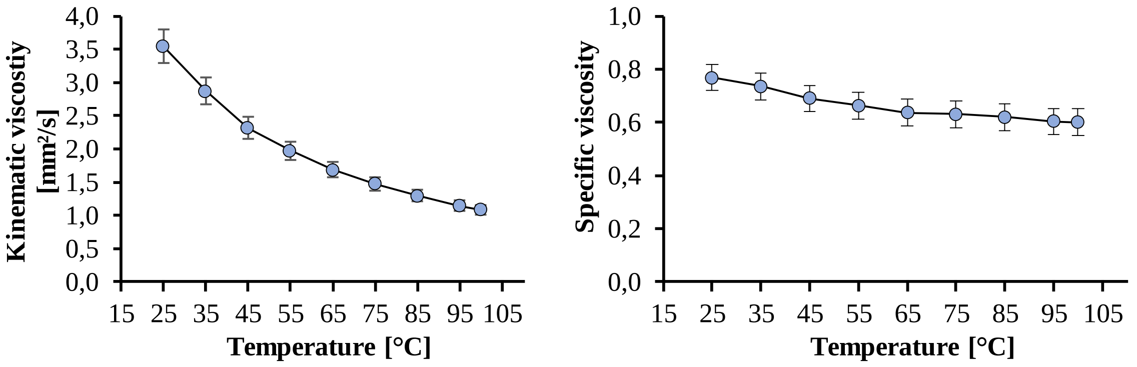

Figure S11: Kinematic (left) and specific (right) viscosity for 1 in DMSO at 120 mM.

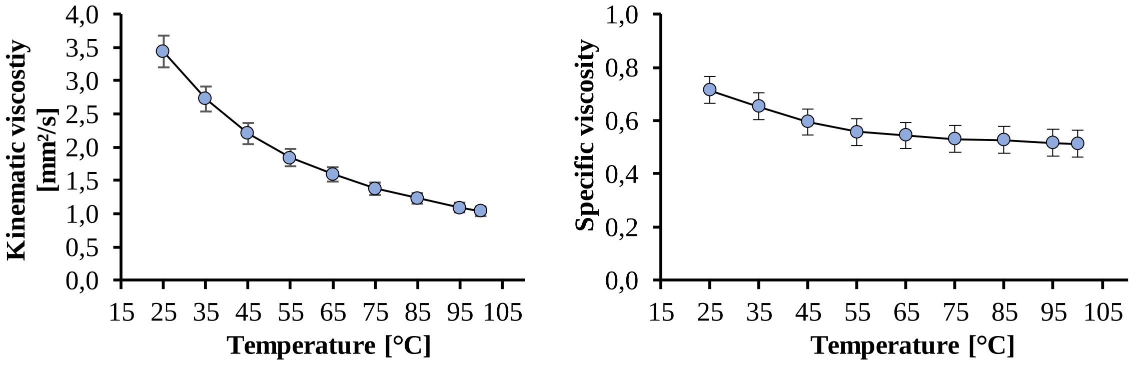

Figure S12: Kinematic (left) and specific (right) viscosity for 1 in DMSO at 100 mM.

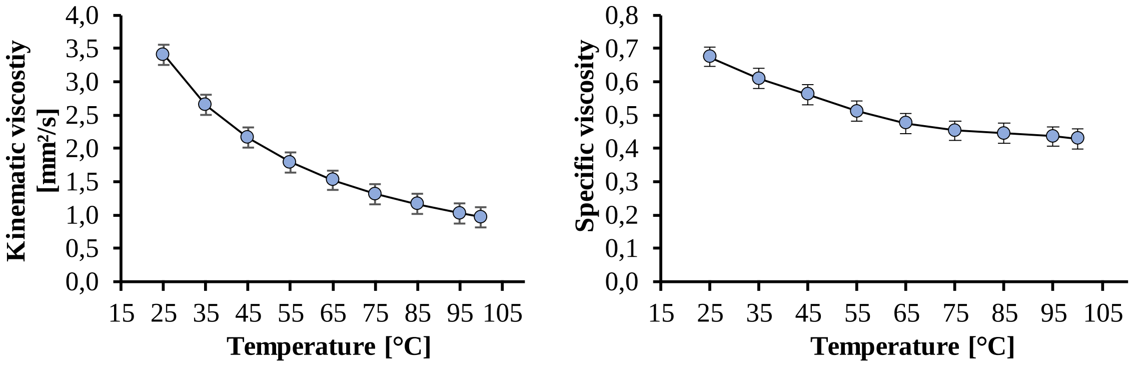

Figure S13: Kinematic (left) and specific (right) viscosity for 1 in DMSO at 80 mM.

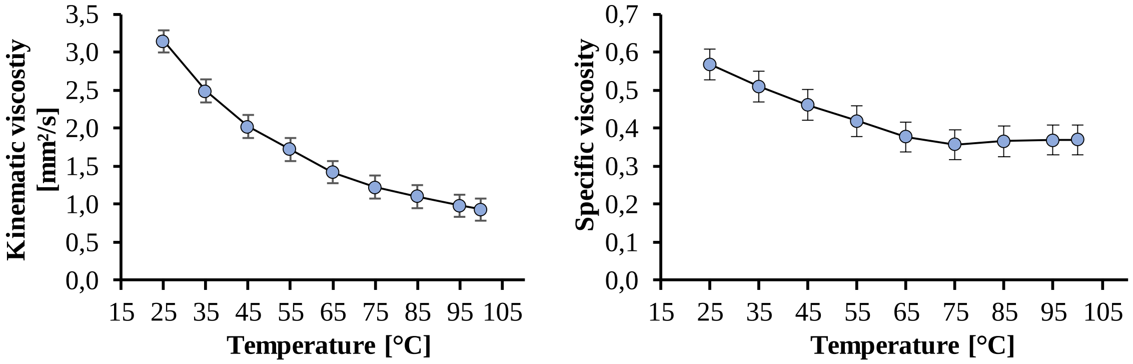

Figure S14: Kinematic (left) and specific (right) viscosity for 1 in DMSO at 60 mM.

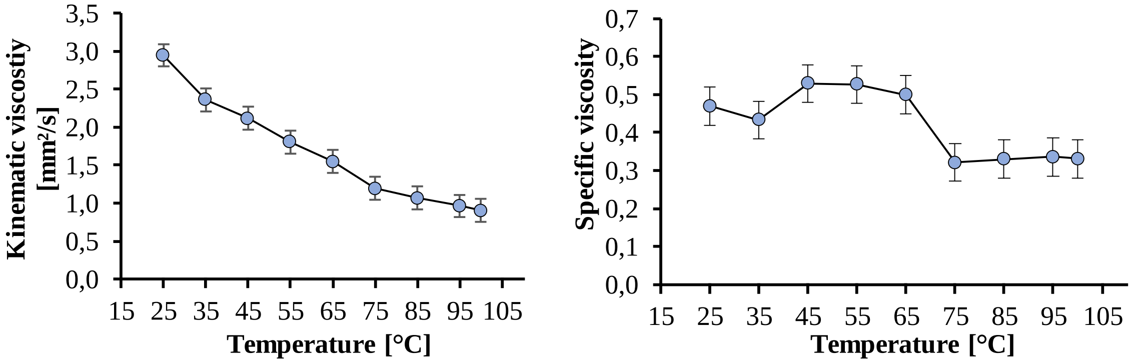

Figure S15: Kinematic (left) and specific (right) viscosity for 1 in DMSO at 50 mM.

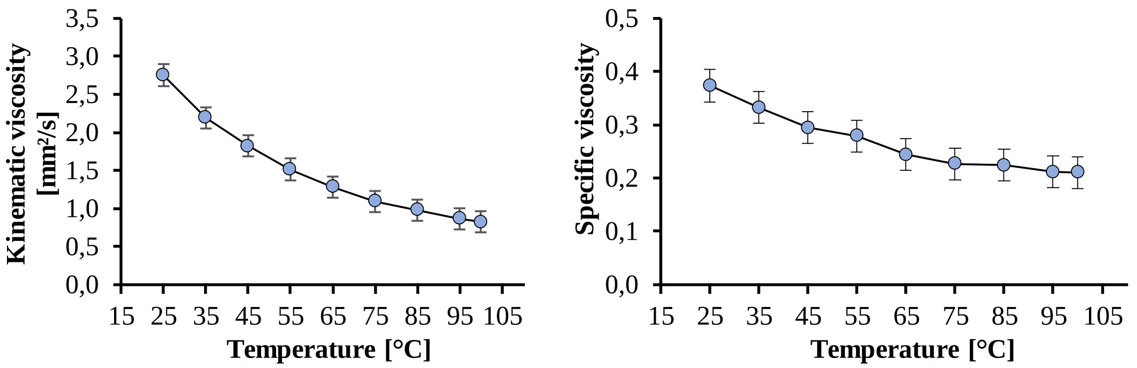

Figure S16: Kinematic (left) and specific (right) viscosity for 1 in DMSO at 40 mM.

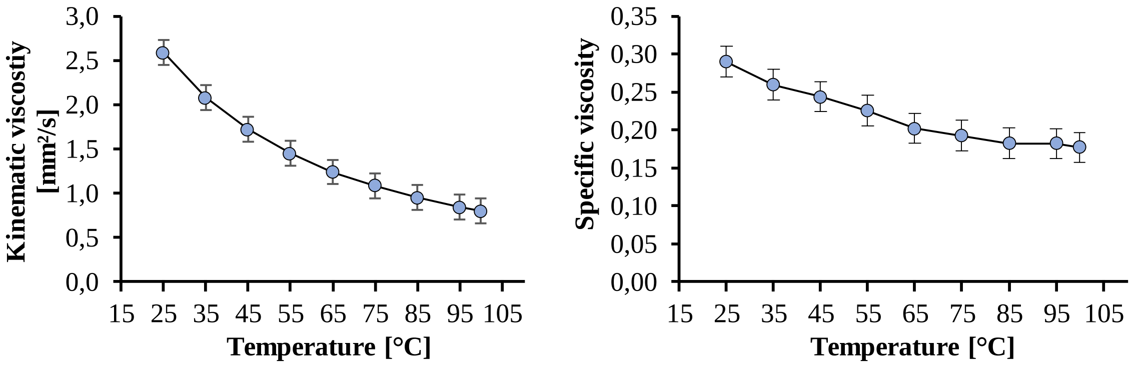

Figure S17: Kinematic (left) and specific (right) viscosity for 1 in DMSO at 30 mM.

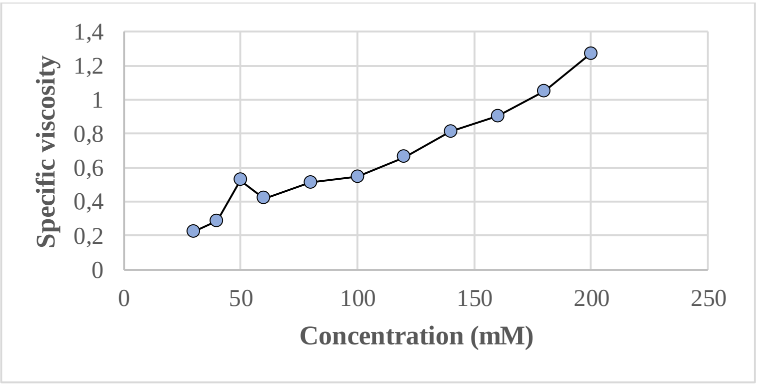

Figure S18: Specific viscosity for 1 in DMSO at 55 °C.

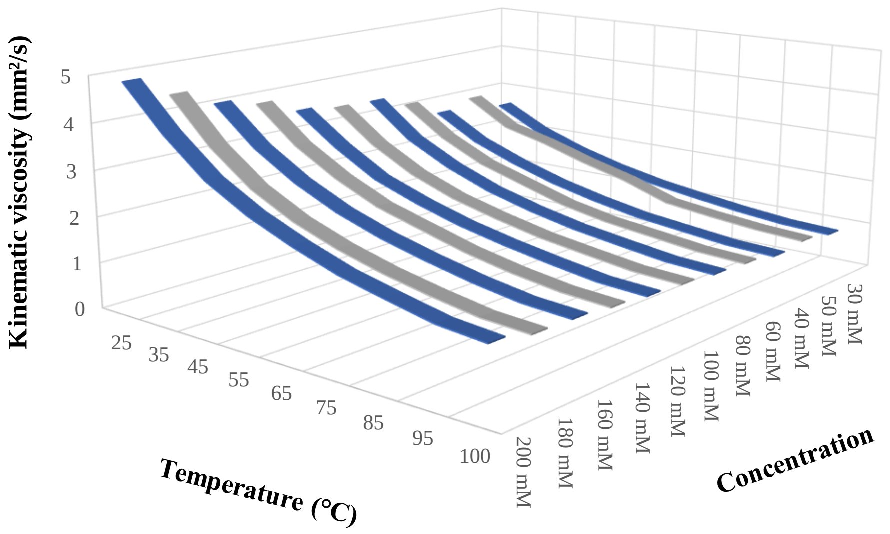

Figure S19: Kinematic viscosity of all measurements of 1 in DMSO.

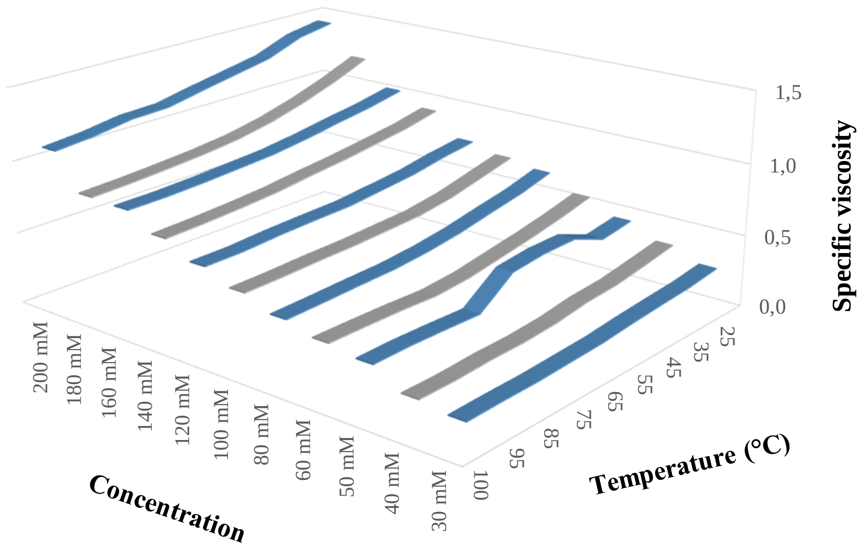

Figure S20: Specific viscosity of all measurements of 1 in DMSO.

6.2 Viscosity measurements of 2

6.2.1 Measurement of 2 in DMSO

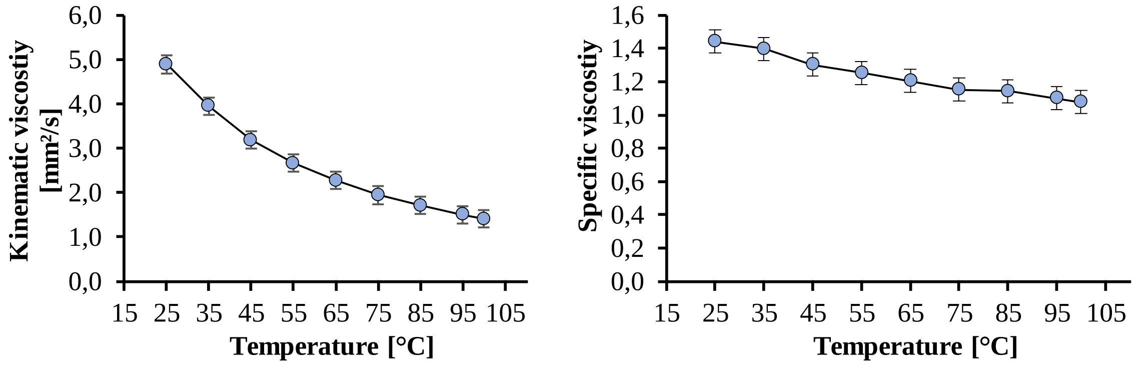

Figure S21: Kinematic (left) and specific (right) viscosity for 2 in DMSO at 200 mM

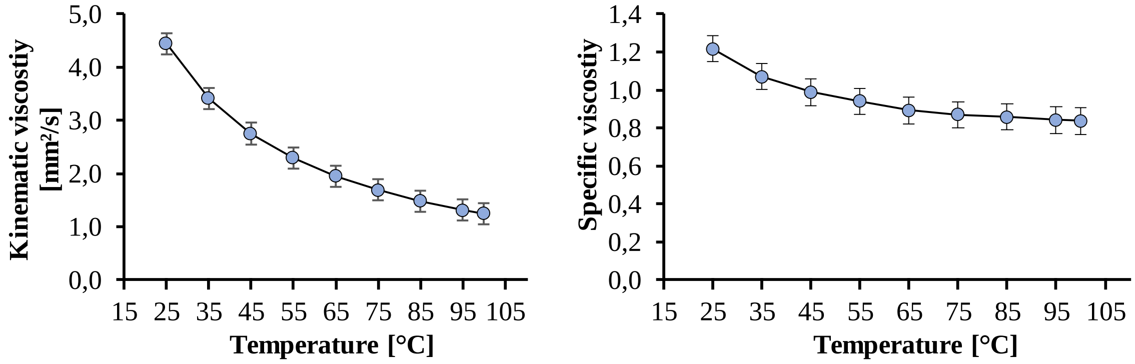

Figure S22: Kinematic (left) and specific (right) viscosity for 2 in DMSO at 180 mM.

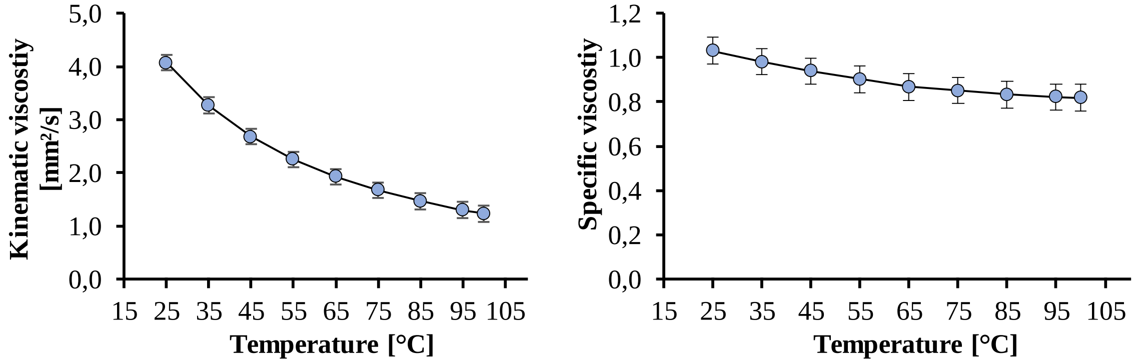

Figure S23: Kinematic (left) and specific (right) viscosity for 2 in DMSO at 160 mM.

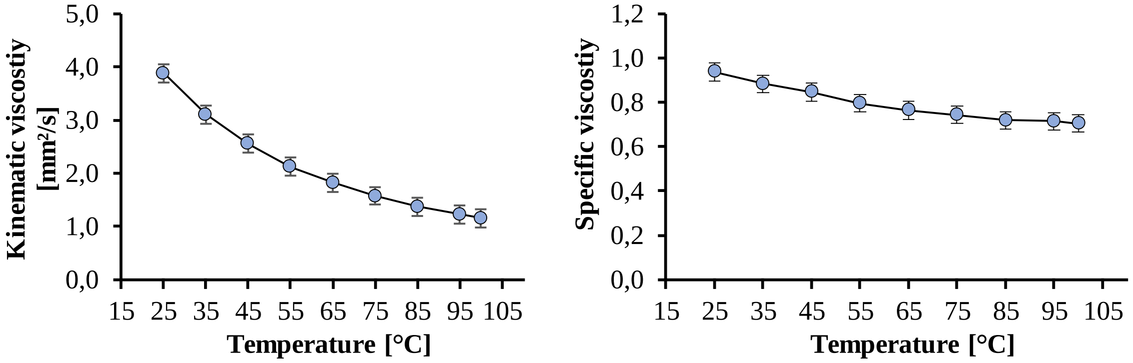

Figure S24: Kinematic (left) and specific (right) viscosity for 2 in DMSO at 140 mM.

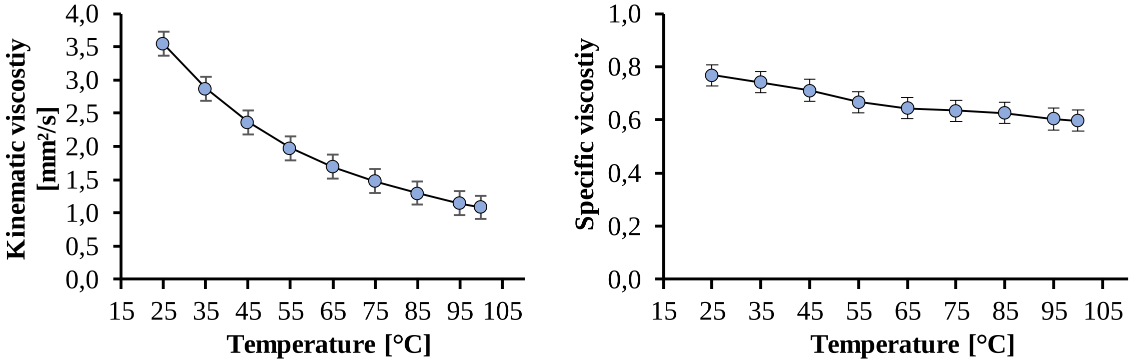

Figure S25: Kinematic (left) and specific (right) viscosity for 2 in DMSO at 120 mM.

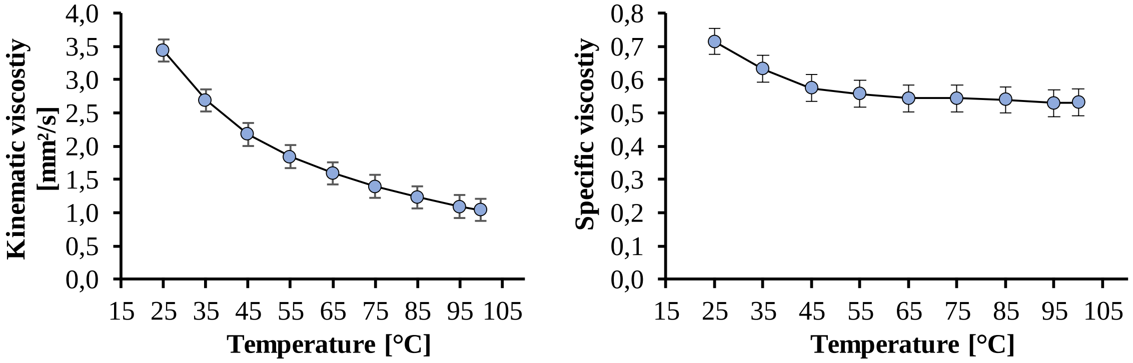

Figure S26: Kinematic (left) and specific (right) viscosity for 2 in DMSO at 100 mM.

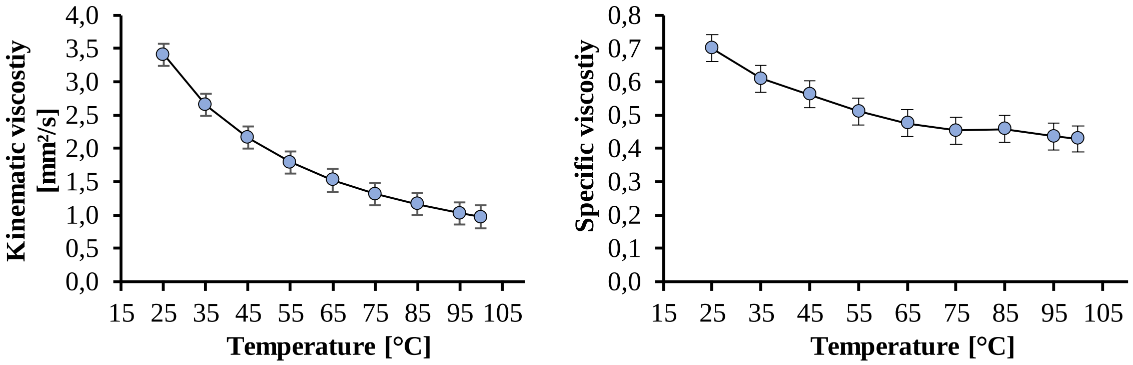

Figure S27: Kinematic (left) and specific (right) viscosity for 2 in DMSO at 80 mM.

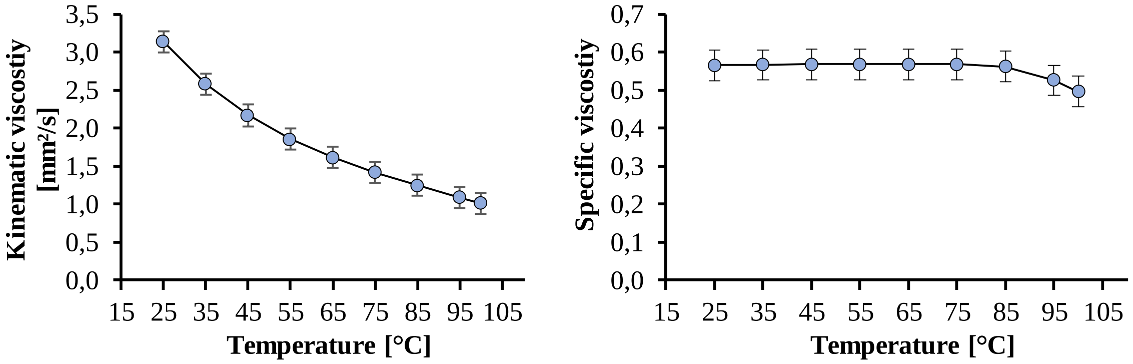

Figure S28: Kinematic (left) and specific (right) viscosity for 2 in DMSO at 60 mM.

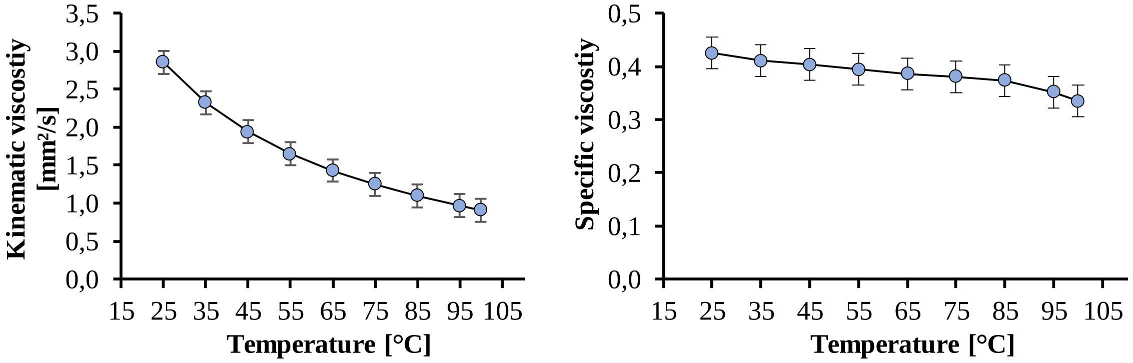

Figure S29: Kinematic (left) and specific (right) viscosity for 2in DMSO at 50 mM.

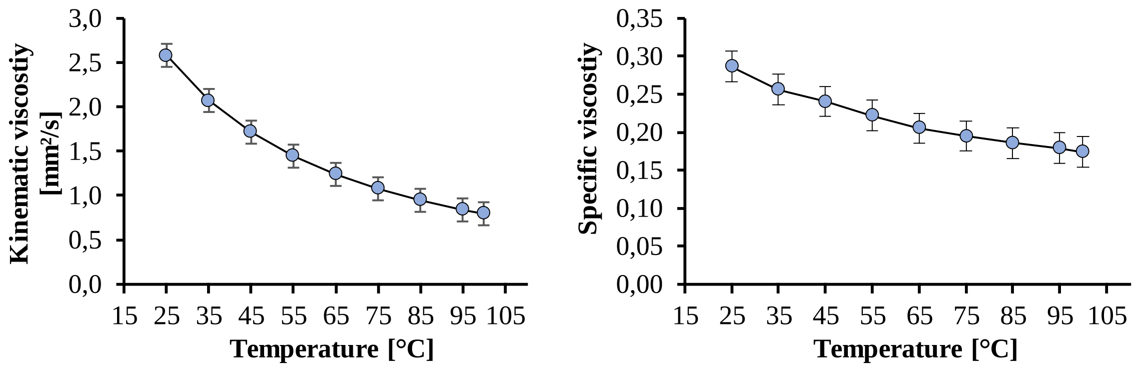

Figure S30: Kinematic (left) and specific (right) viscosity for 2 in DMSO at 40 mM.

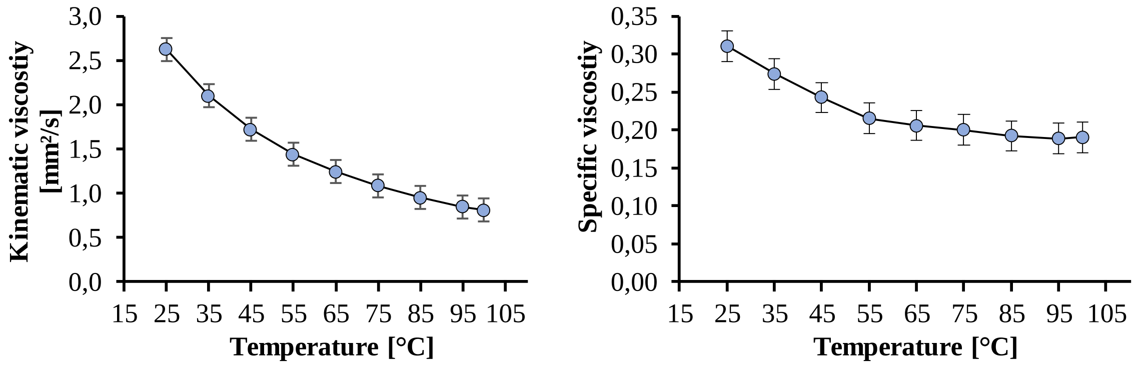

Figure S31: Kinematic (left) and specific (right) viscosity for 2 in DMSO at 30 mM.

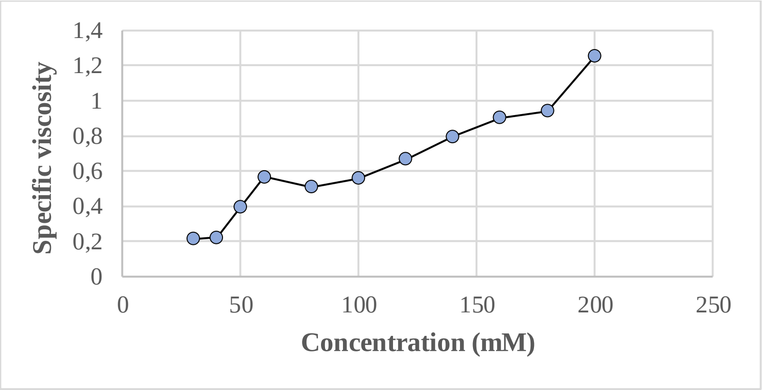

Figure S32: Specific viscosity for 2 in DMSO at 55 °C.

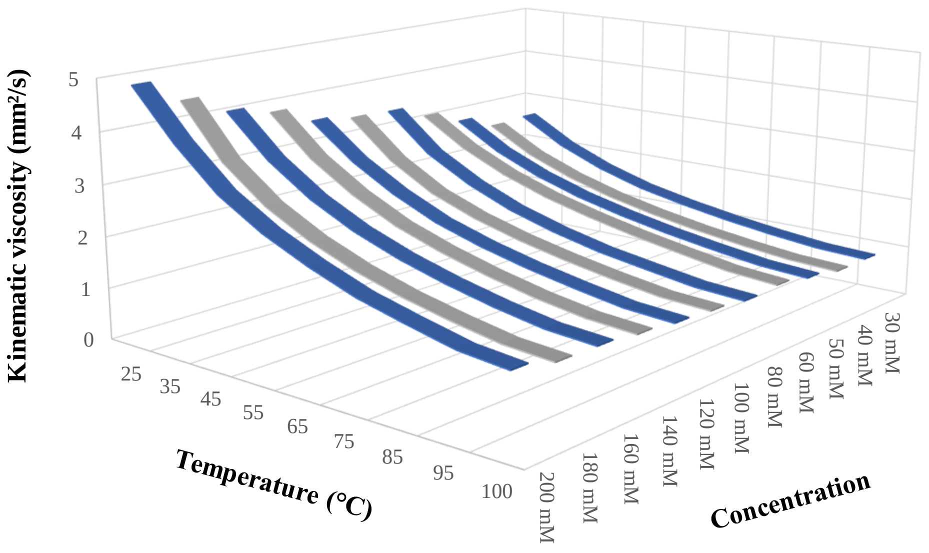

Figure S33: Kinematic viscosity of all measurements of 2 in DMSO.

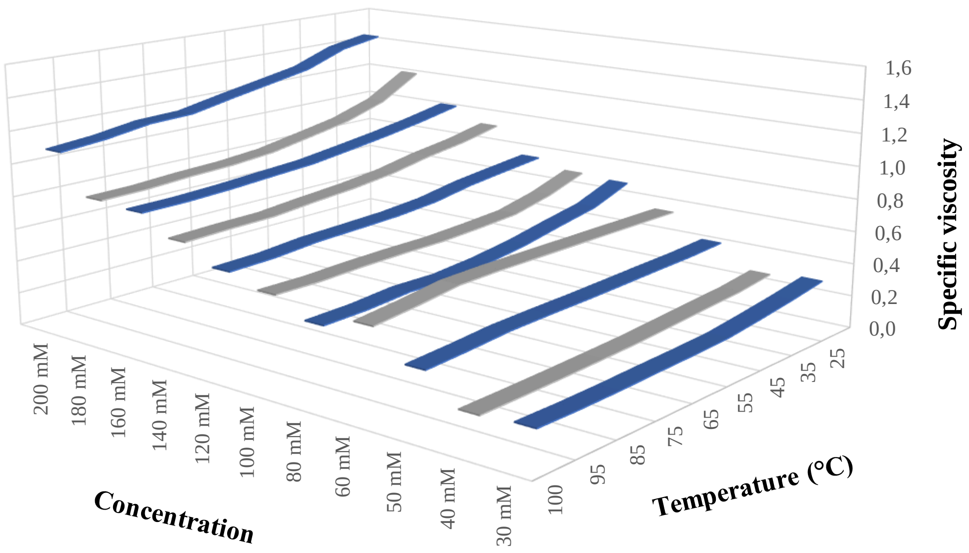

Figure S34: Specific viscosity of all measurements of 2 in DMSO.

6.2.2 Measurement of 2 in chloroform

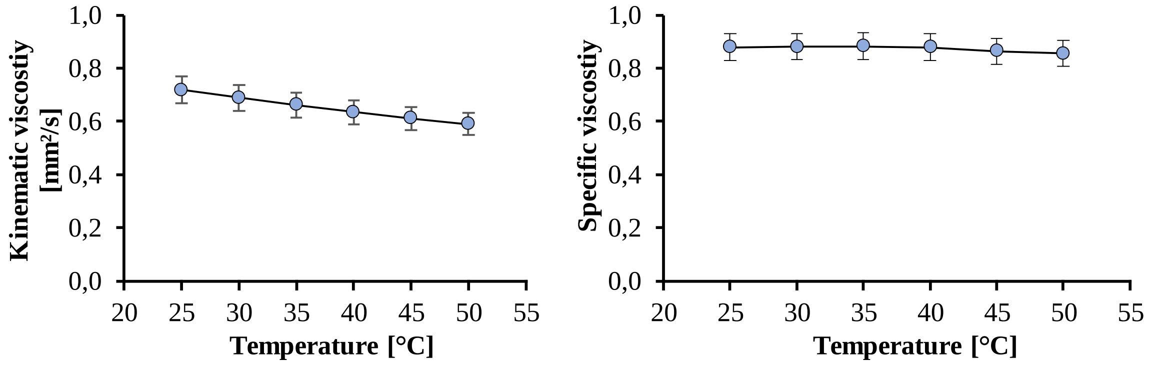

Figure S35: Kinematic (left) and specific (right) viscosity for 2 in chloroform at 100 mM.

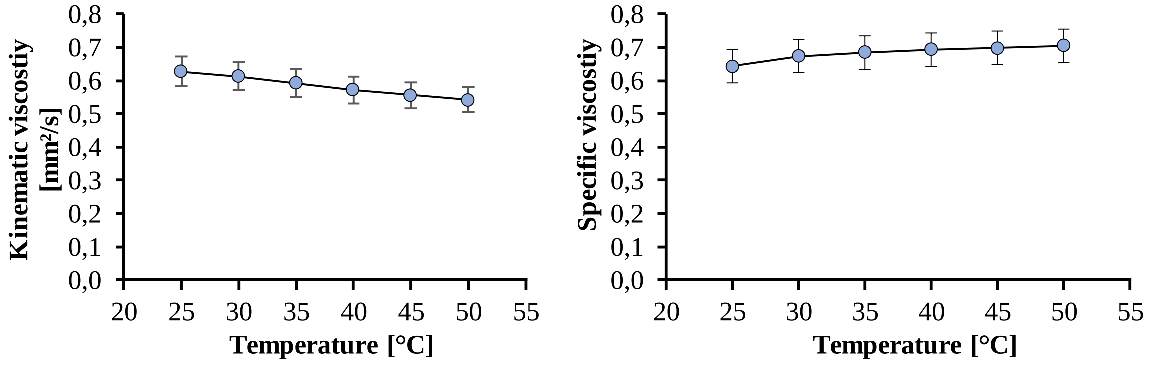

Figure S36: Kinematic (left) and specific (right) viscosity for 2 in chloroform at 80 mM.

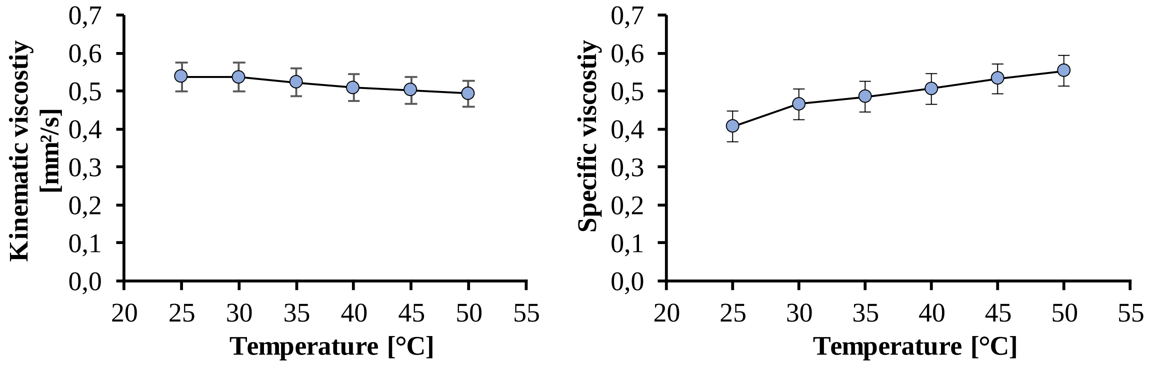

Figure S37: Kinematic (left) and specific (right) viscosity for 2 in chloroform at 60 mM.

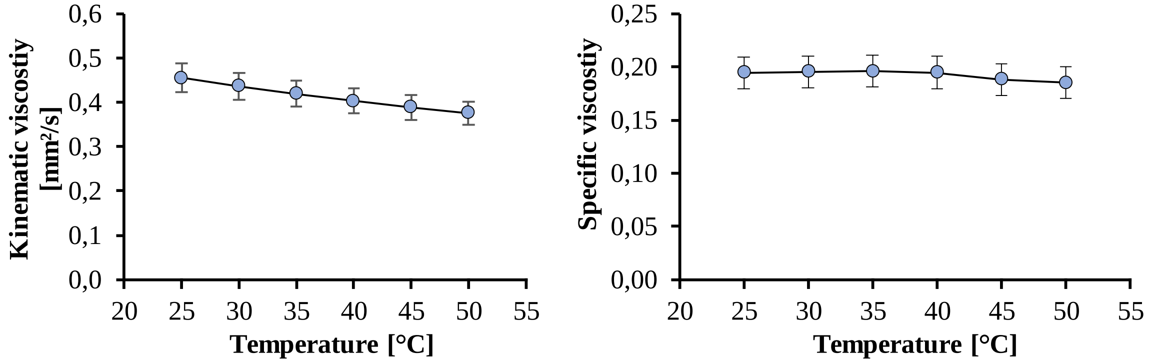

Figure S38: Kinematic (left) and specific (right) viscosity for 2 in chloroform at 40 mM.

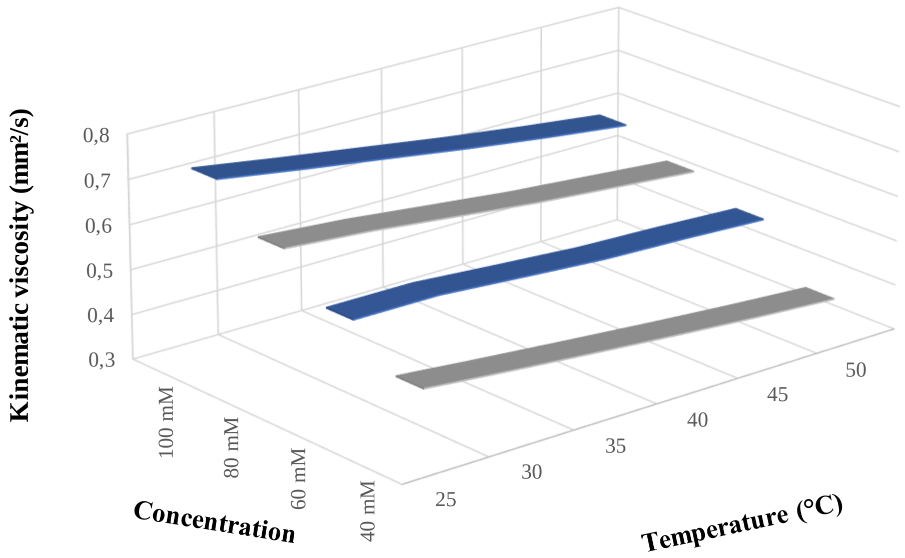

Figure S39: Kinematic viscosity of all measurements of 2 in chloroform.

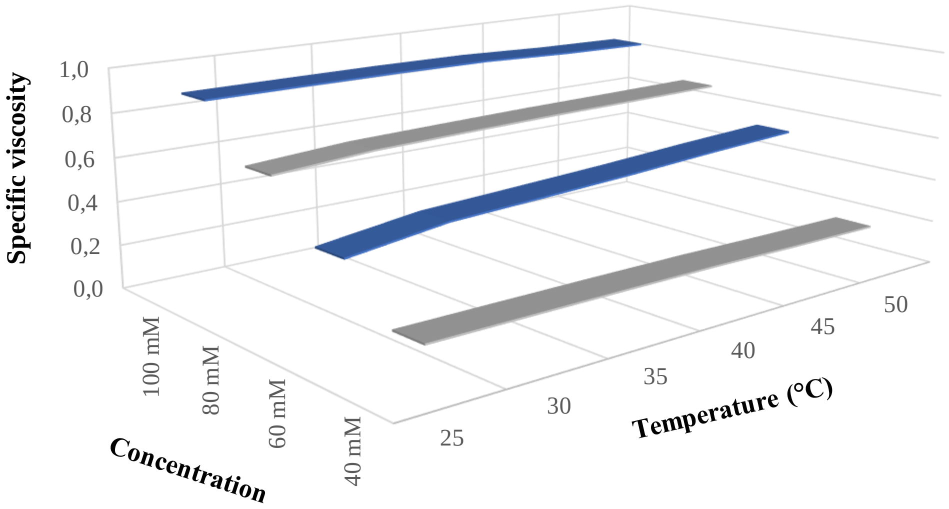

Figure S40: Specific viscosity of all measurements of 2 in chloroform.

6.2.3 Measurement of 2 in toluene

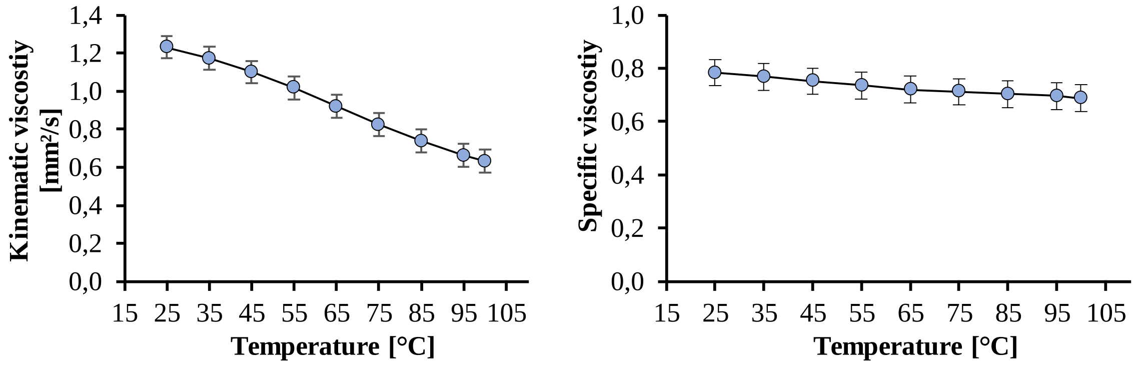

Figure S41: Kinematic (left) and specific (right) viscosity for 2 in toluene at 100 mM.

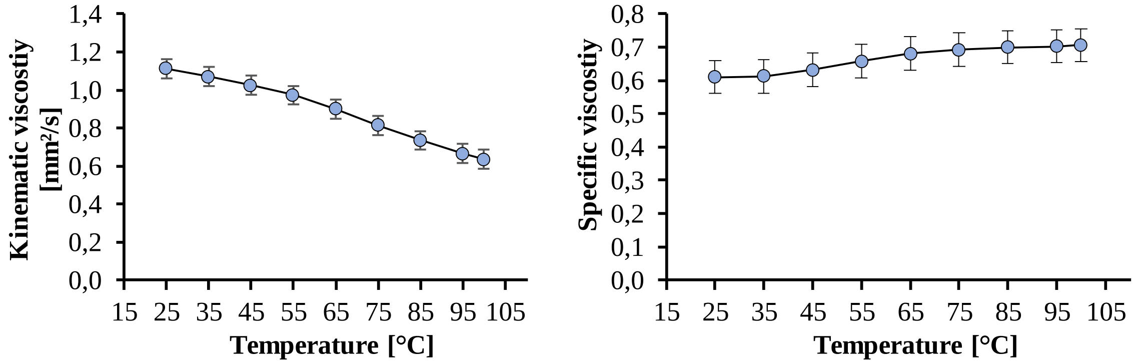

Figure S42: Kinematic (left) and specific (right) viscosity for 2 in toluene at 80 mM.

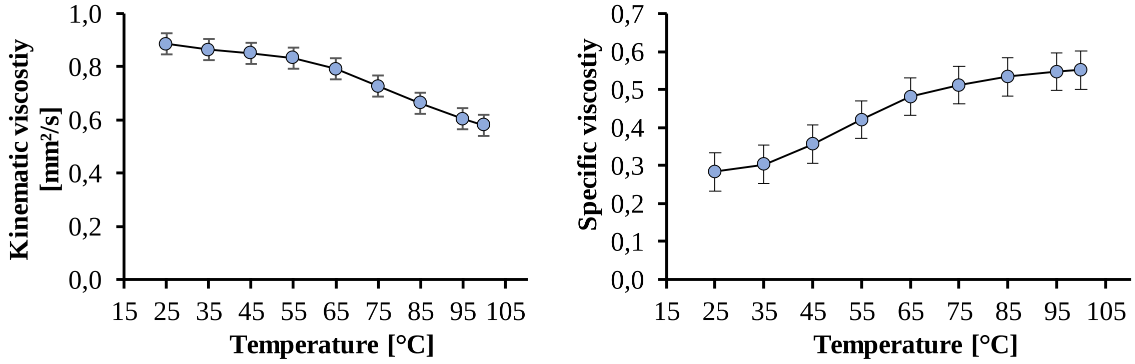

Figure S43: Kinematic (left) and specific (right) viscosity for 2 in toluene at 60 mM.

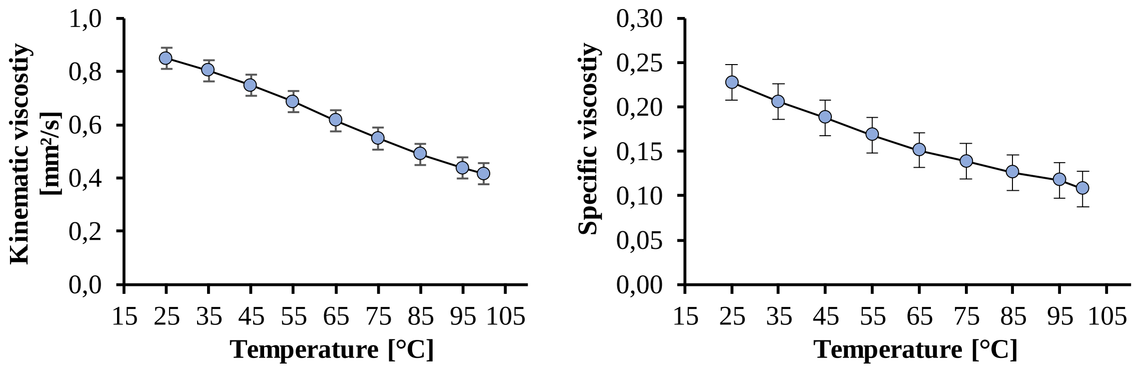

Figure S44: Kinematic (left) and specific (right) viscosity for 2 in toluene at 40 mM.

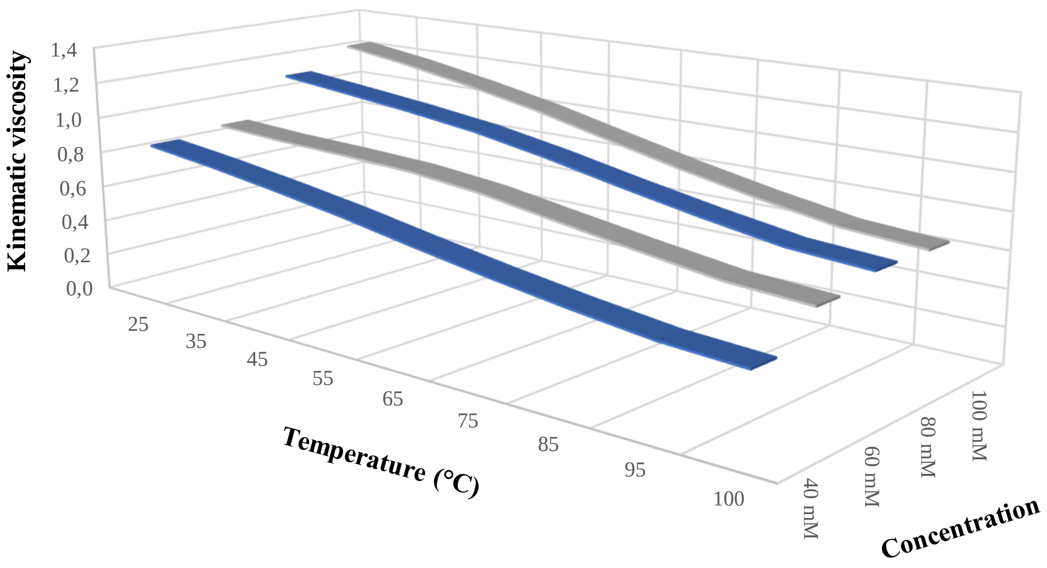

Figure S45: Kinematic viscosity of all measurements of 2 in toluene.

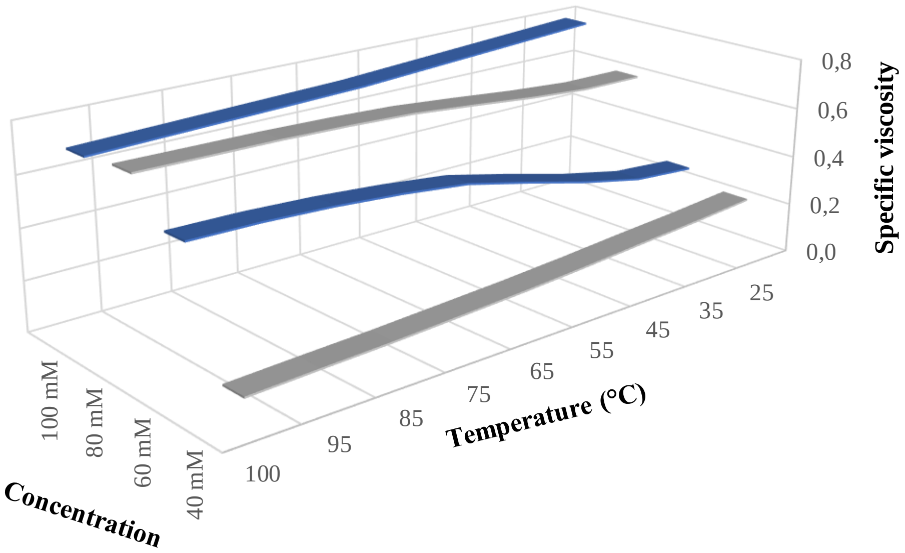

Figure S46: Specific viscosity of all measurements of 2 in toluene.

6.2.4 Measurement of 2 in Nynas NS8

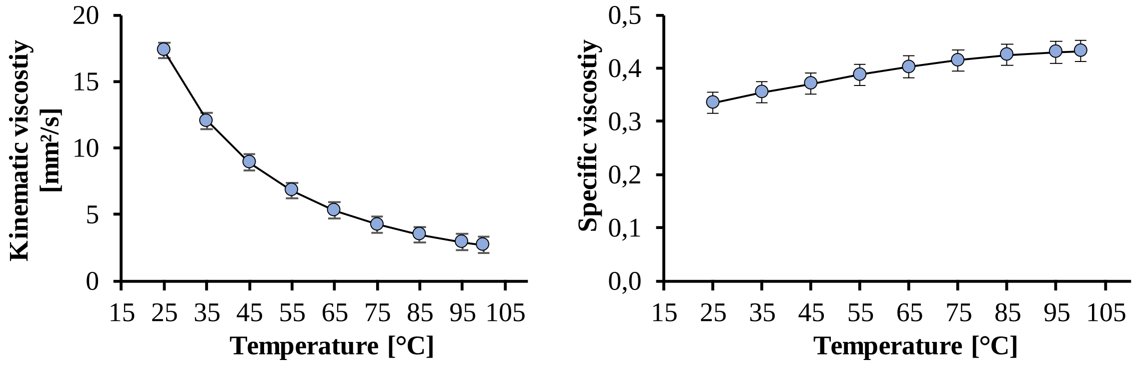

Figure S47: Kinematic (left) and specific (right) viscosity for 2 in Nynas NS8 at 60 mM.

6.3 Viscosity measurements of 3

6.3.1 Measurement of 3 in Nynas NS8

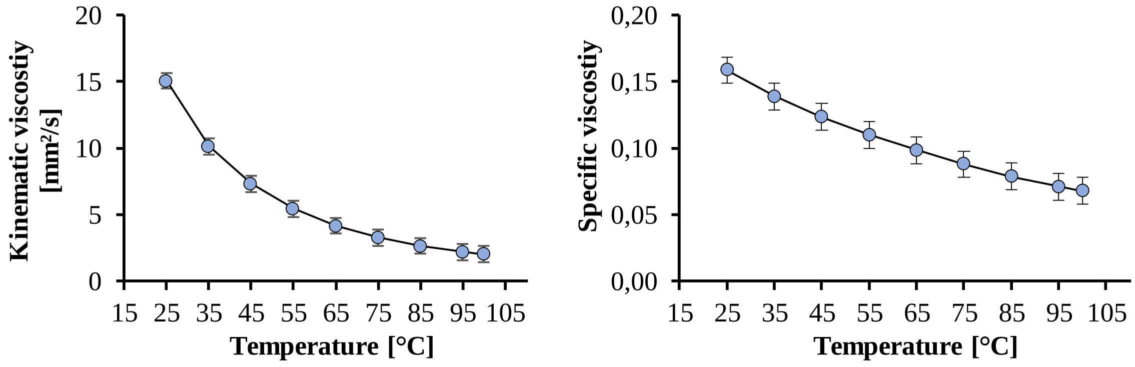

Figure S48: Kinematic (left) and specific (right) viscosity for 3 in Nynas NS8 at 100 mM.

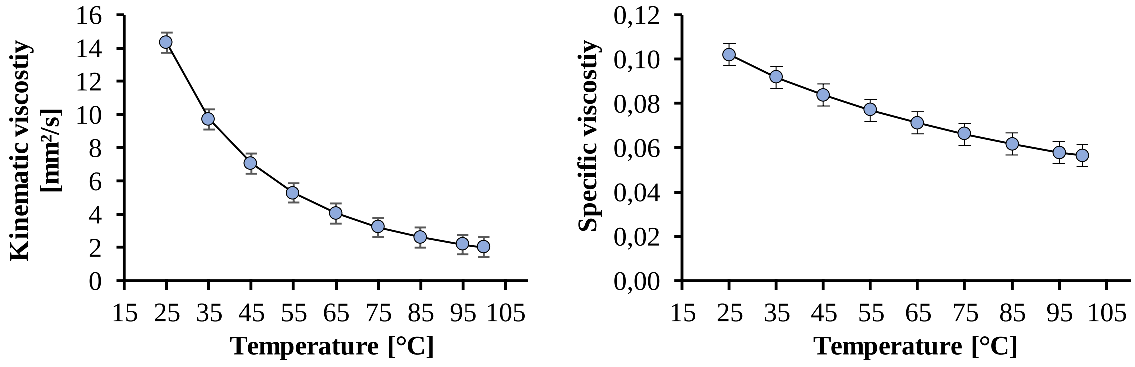

Figure S49: Kinematic (left) and specific (right) viscosity for 3 in Nynas NS8 at 80 mM.

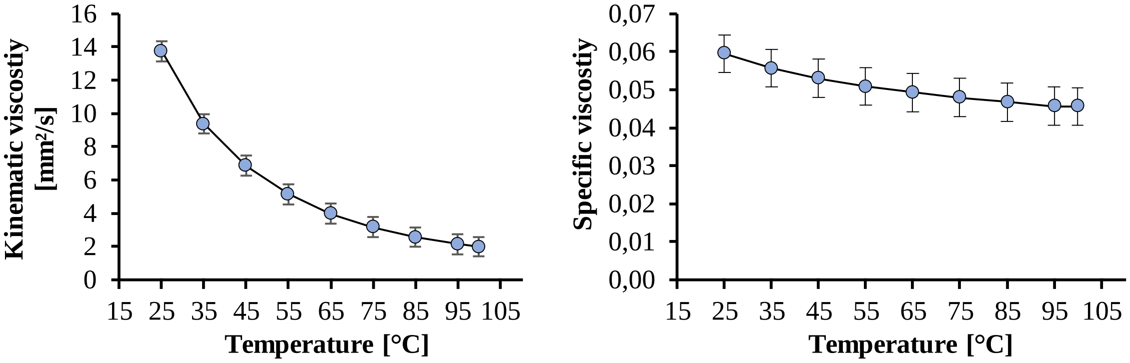

Figure S50: Kinematic (left) and specific (right) viscosity for 3 in Nynas NS8 at 60 mM.

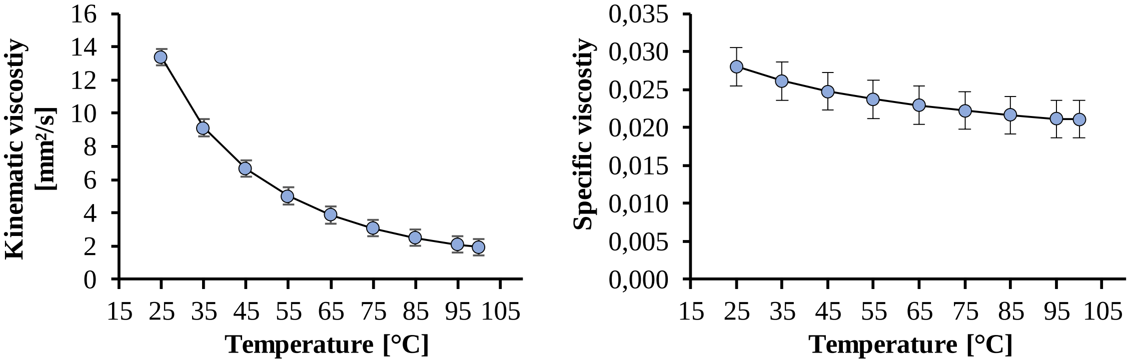

Figure S51: Kinematic (left) and specific (right) viscosity for 3 in Nynas NS8 at 40 mM.

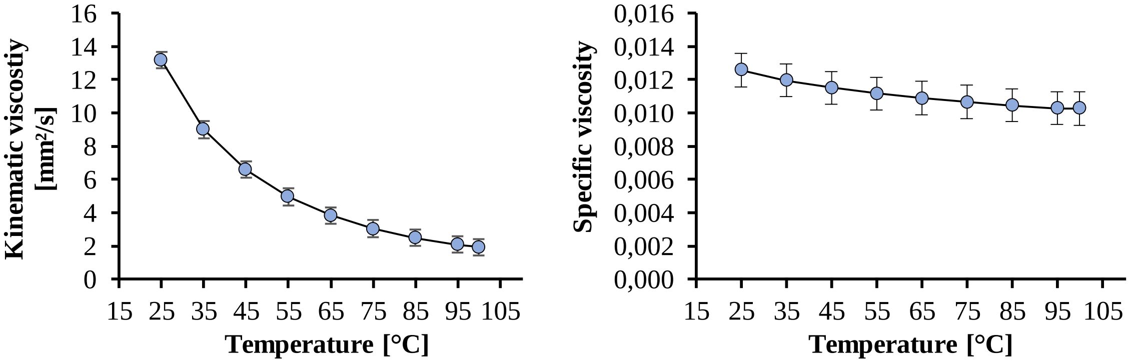

Figure S52: Kinematic (left) and specific (right) viscosity for 3 in Nynas NS8 at 20 mM.

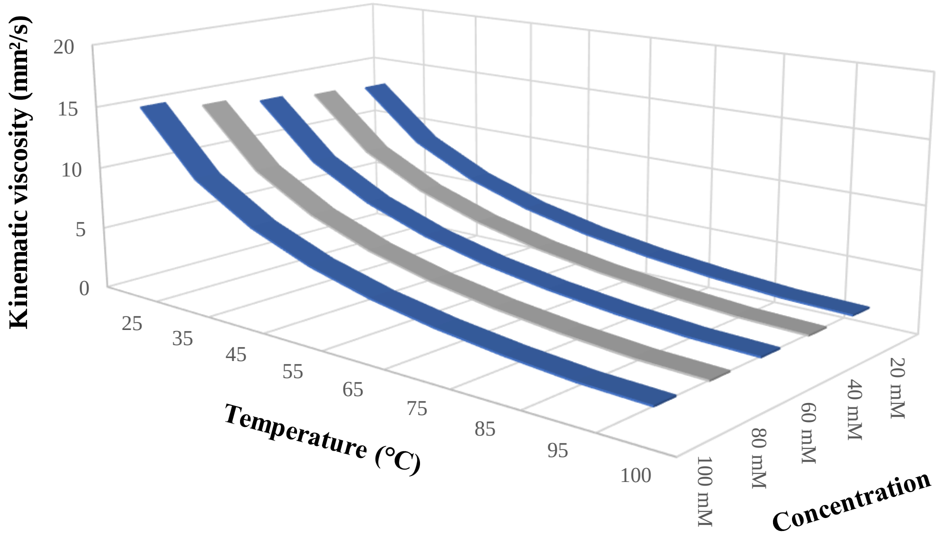

Figure S53: Kinematic viscosity of all measurements of 3 in Nynas NS8.

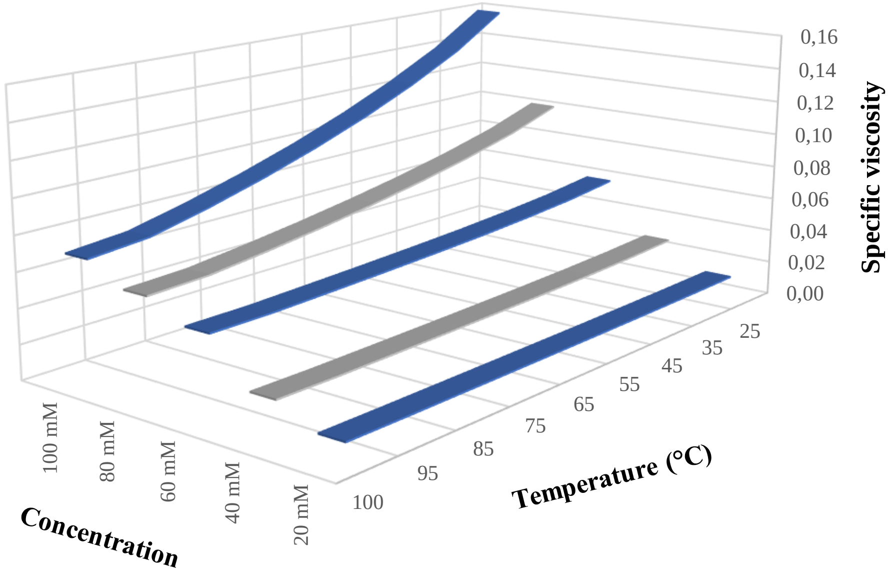

Figure S54: Specific viscosity of all measurements of 3 in Nynas NS8.

6.3.2 Measurement of 3 in Nexbase 3020

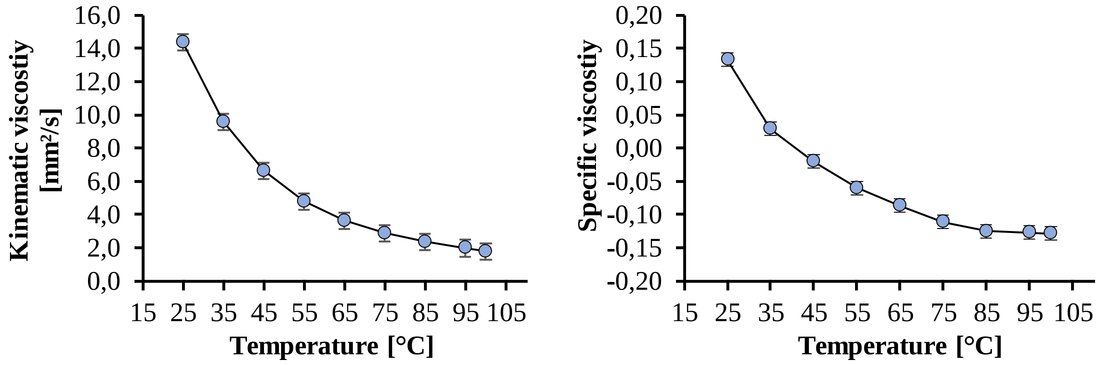

Figure S55: Kinematic (left) and specific (right) viscosity for 3 in Nexbase 3020 at 40 mM.

6.3.2 Measurement of 3 in Toluene

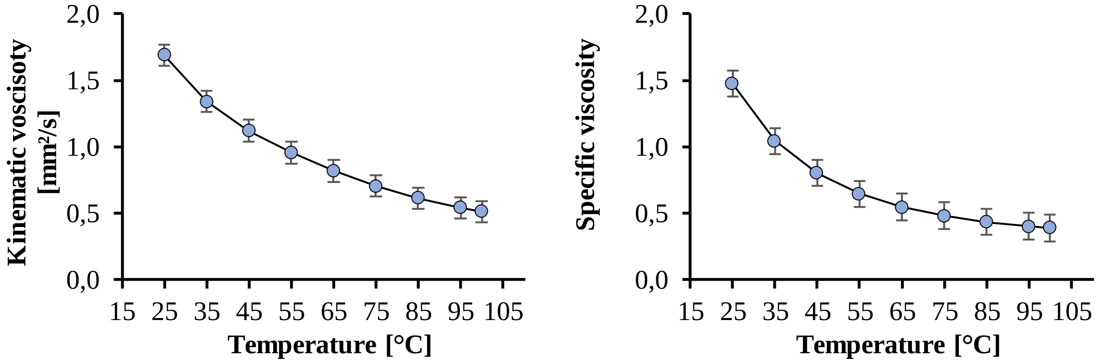

Figure S56: Kinematic (left) and specific (right) viscosity for 3 in toluene at 100 mM.

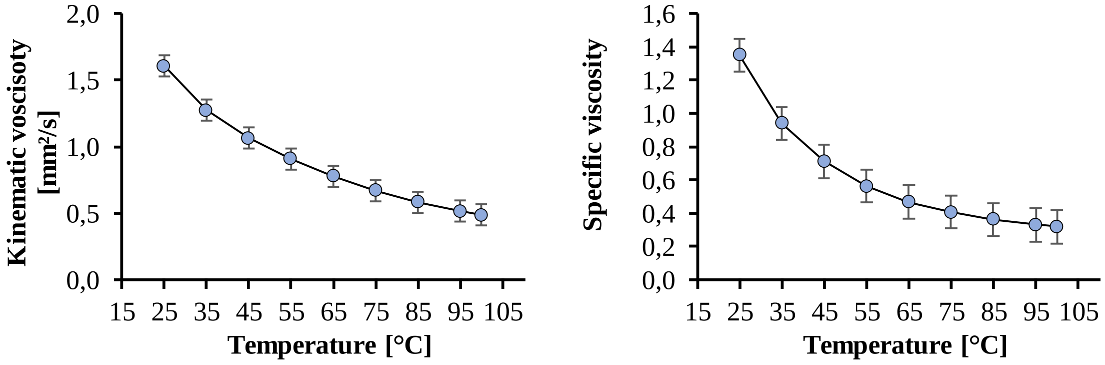

Figure S57: Kinematic (left) and specific (right) viscosity for 3 in toluene at 80 mM.

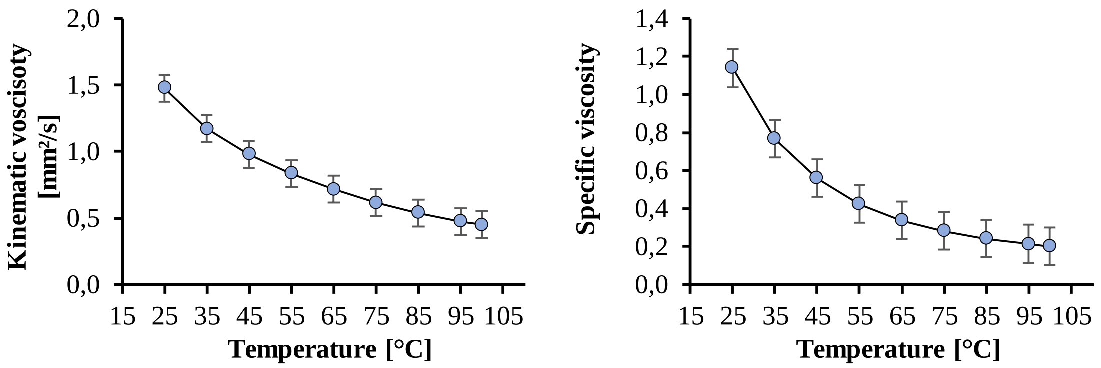

Figure S58: Kinematic (left) and specific (right) viscosity for 3 in toluene at 60 mM.

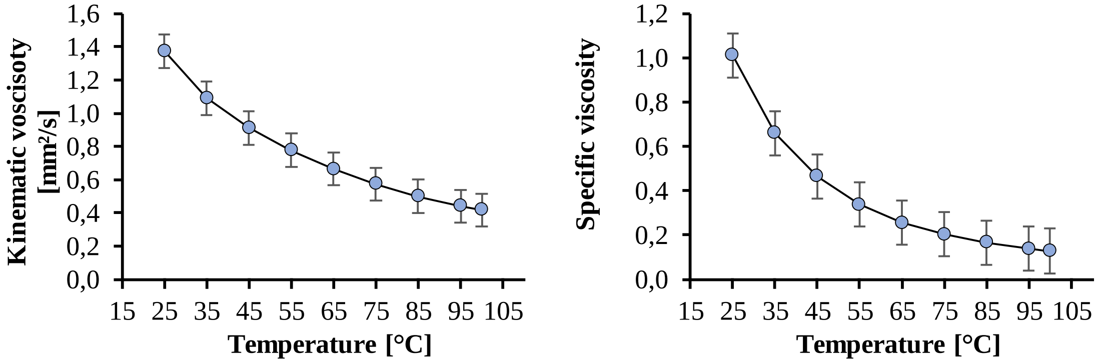

Figure S59: Kinematic (left) and specific (right) viscosity for 3 in toluene at 40 mM.

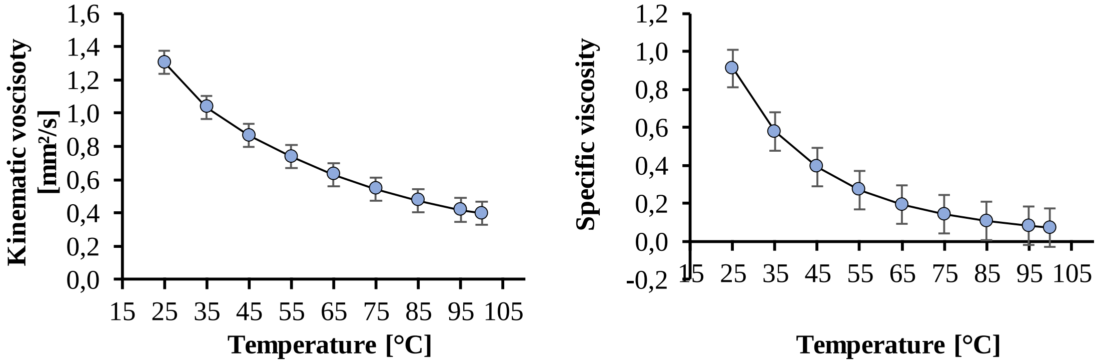

Figure S60: Kinematic (left) and specific (right) viscosity for 3 in toluene at 30 mM.

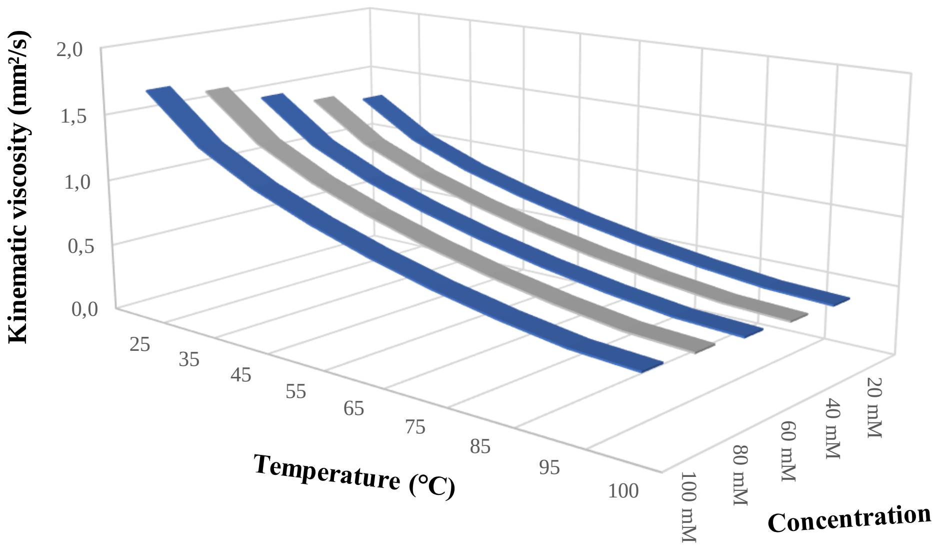

Figure S61: Kinematic viscosity of all measurements of 3 in toluene.

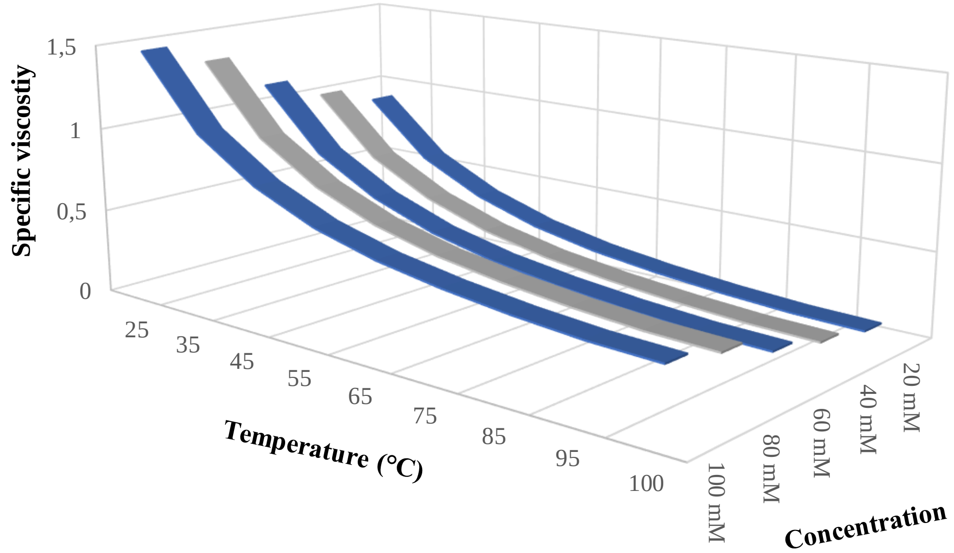

Figure S62: Specific viscosity of all measurements of 3 in toluene.

6.4 Viscosity measurement of 4

6.4.1 Measurement of 4 in DMSO

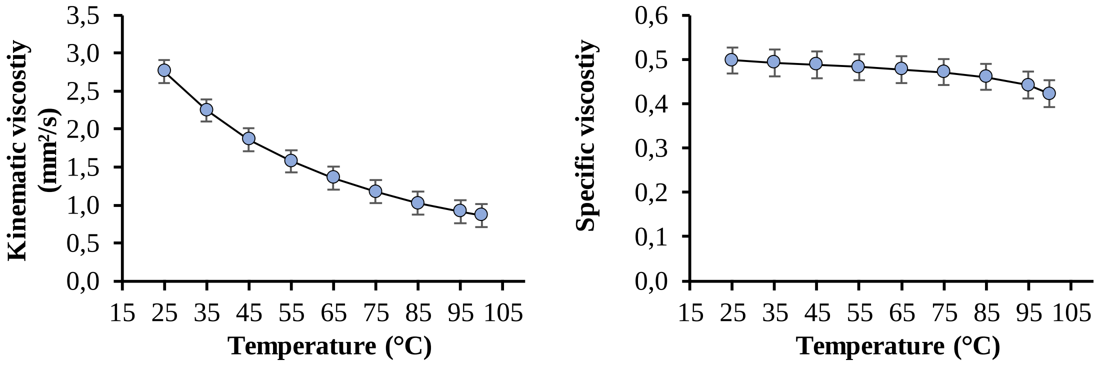

Figure S63: Kinematic (left) and specific (right) viscosity for 4 in DMSO at 60 mM.

6.4.2 Measurement of 4 in chloroform

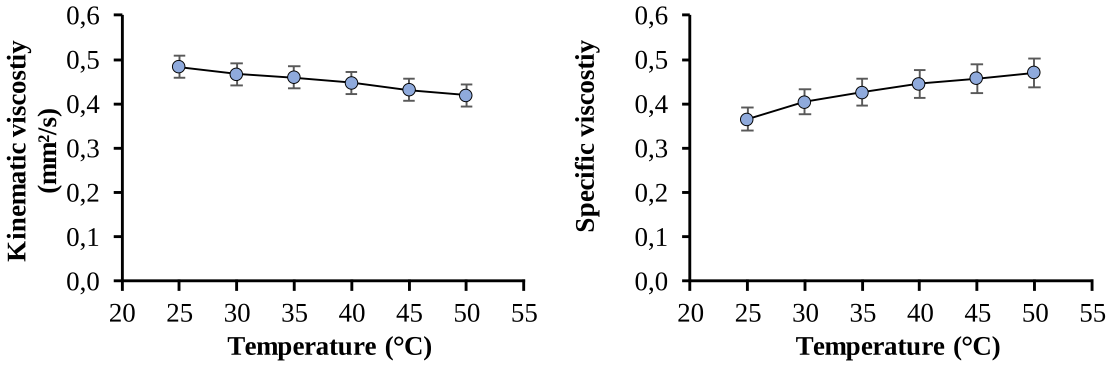

Figure S64: Kinematic (left) and specific (right) viscosity for 4 in DMSO at 60 mM.

6.4.3 Measurement of 4 in toluene

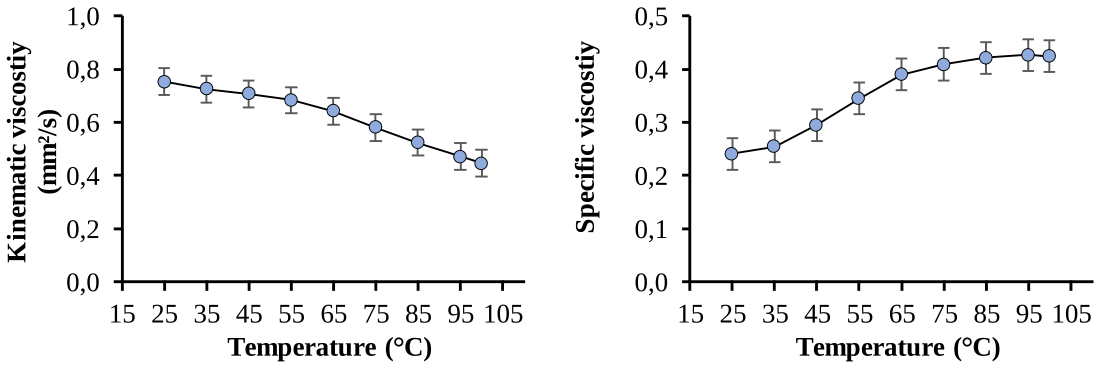

Figure S65: Kinematic (left) and specific (right) viscosity for 4 in toluene at 60 mM.

6.4.4 Measurement of 4 in Nynas NS8

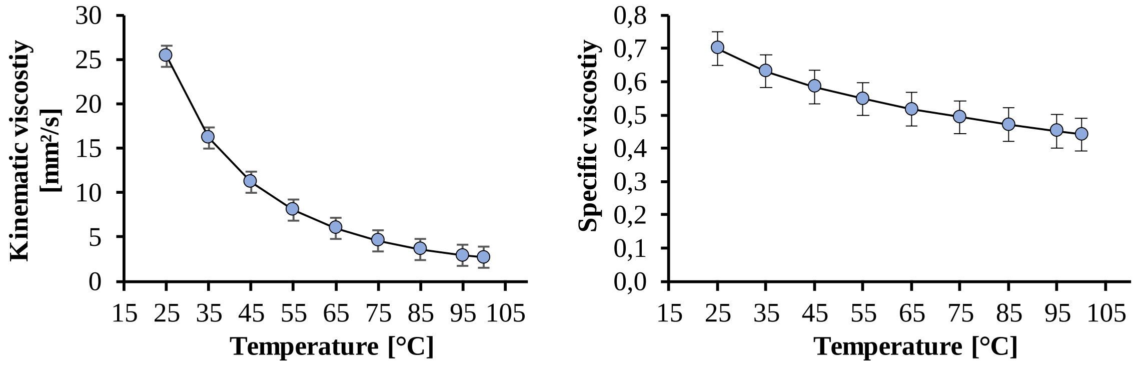

Figure S66: Kinematic (left) and specific (right) viscosity for 4 in Nynas NS8 at 100 mM.

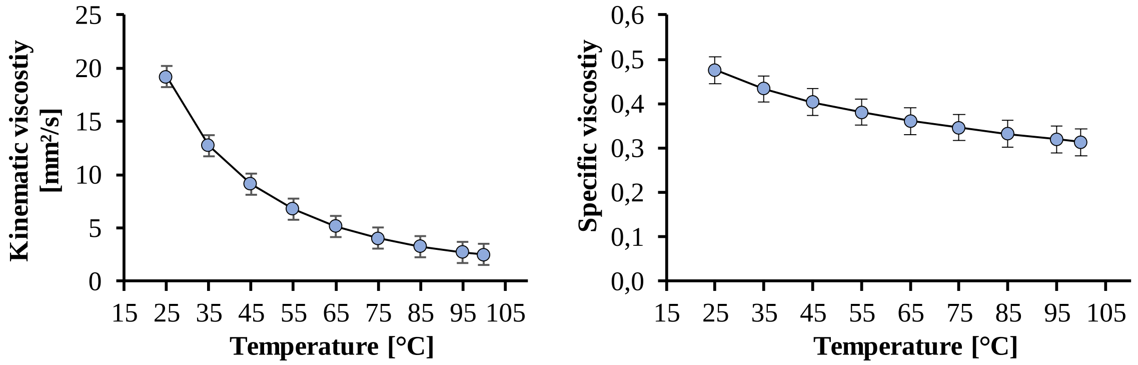

Figure S67: Kinematic (left) and specific (right) viscosity for 4 in Nynas NS8 at 80 mM.

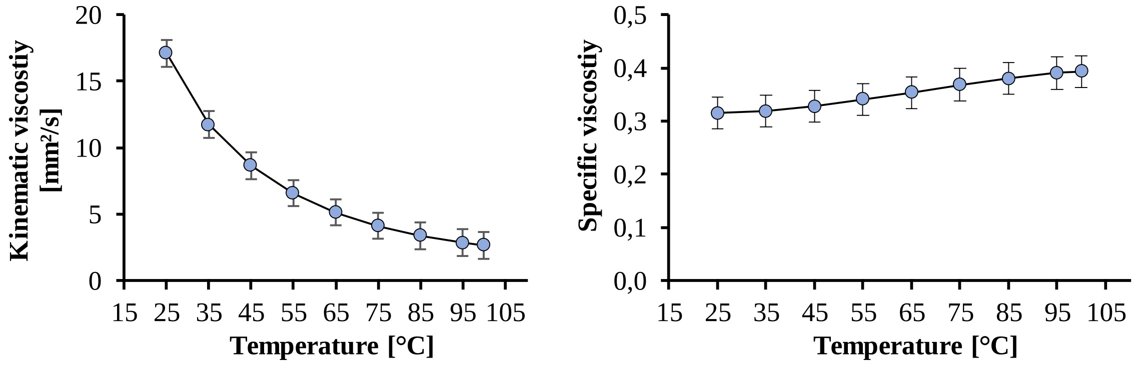

Figure S68: Kinematic (left) and specific (right) viscosity for 4 in Nynas NS8 at 60 mM.

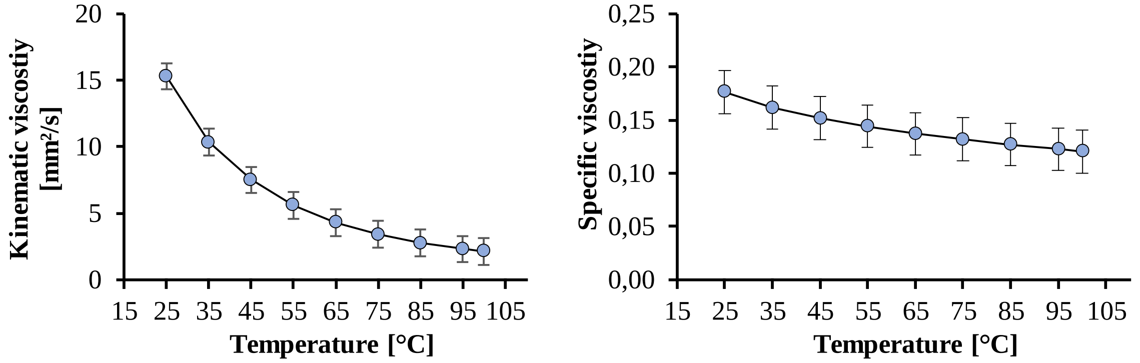

Figure S69: Kinematic (left) and specific (right) viscosity for 4 in Nynas NS8 at 40 mM.

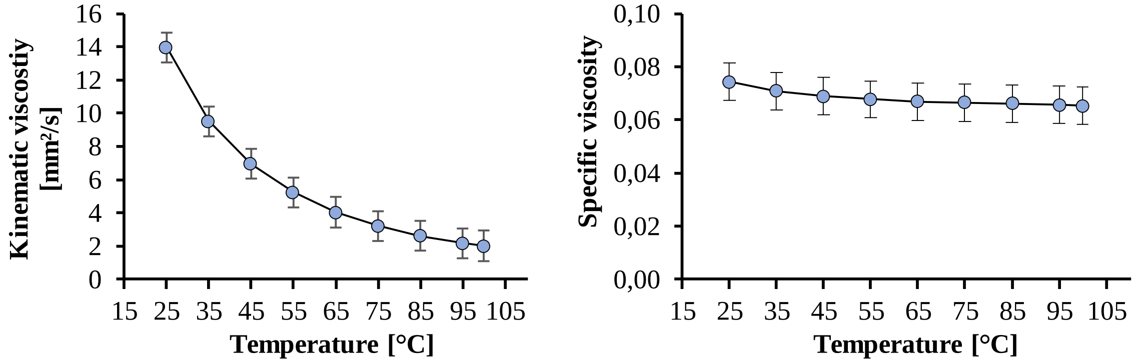

Figure S70: Kinematic (left) and specific (right) viscosity for 4 in Nynas NS8 at 20 mM.

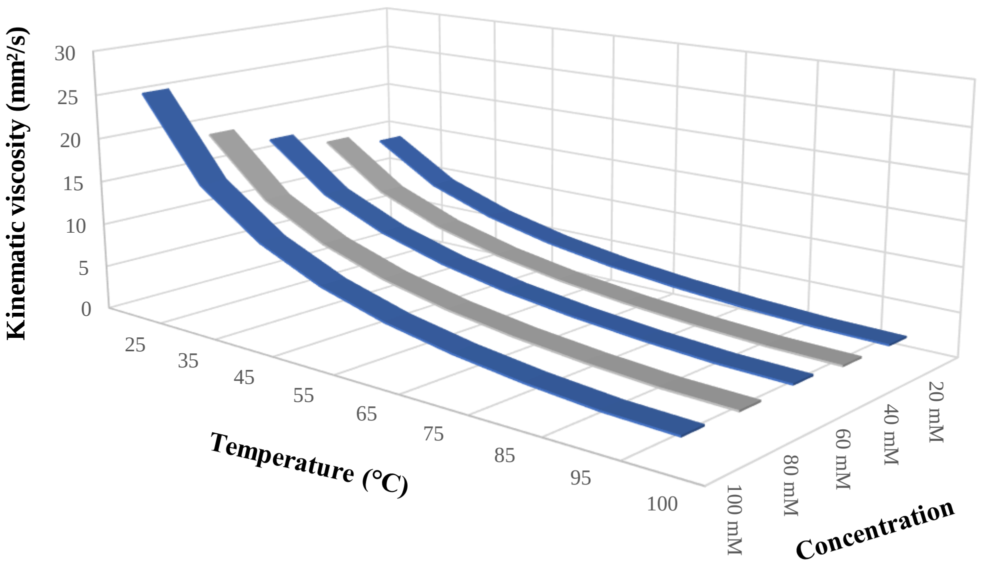

Figure S71: Kinematic viscosity of all measurements of 4 in Nynas NS8.

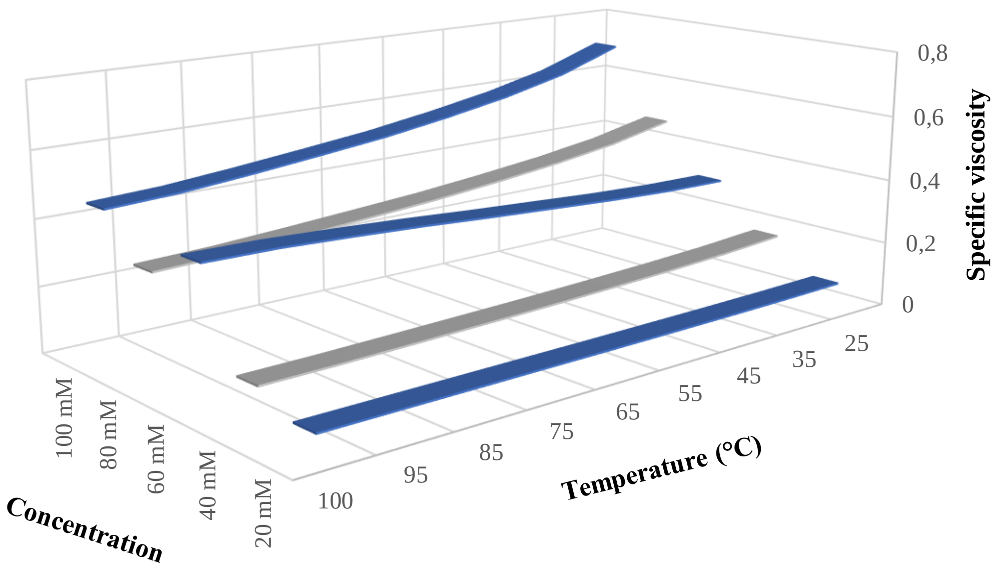

Figure S72: Specific viscosity of all measurements of 4 in Nynas NS8.

6.4.5 Measurement of 4 in Nexbase 3020

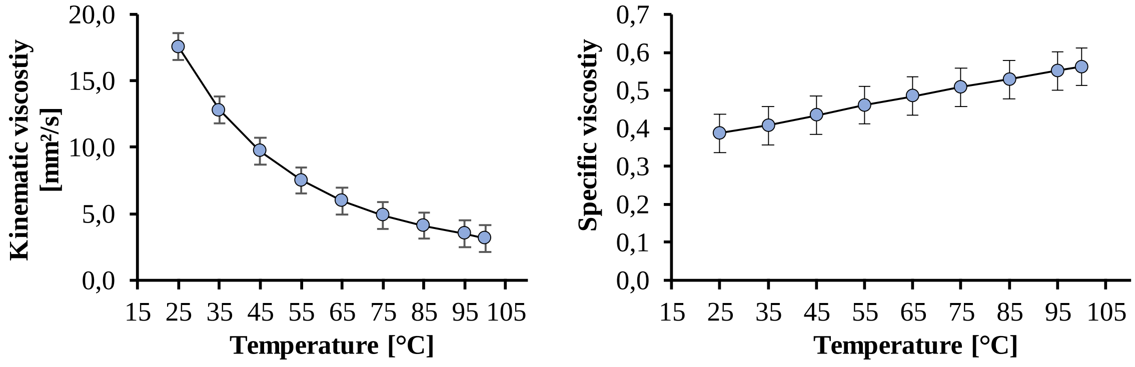

Figure S73: Kinematic (left) and specific (right) viscosity for 4 in Nexbase 3020 at 60 mM.

Table S1: Viscosity measurements of 1 in DMSO.

	Temp (°C)	25	35	45	55	65	75	85	95	100
200 mM	Kin. V. (mm^2/s)	4.891	3.945	3.184	2.666	2.274	1.944	1.711	1.500	1.409
	Specific V.	1.441	1.398	1.302	1.273	1.205	1.152	1.133	1.101	1.077
180 mM	Kin. V. (mm^2/s)	4.443	3.486	2.776	2.296	1.952	1.689	1.484	1.314	1.252
	Specific V.	1.218	1.116	1.030	1.052	0.910	0.880	0.859	0.842	0.830
160 mM	Kin. V. (mm^2/s)	4.071	3.265	2.688	2.251	1.927	1.680	1.466	1.300	1.234
	Specific V.	1.032	0.982	0.944	0.902	0.868	0.850	0.833	0.822	0.819
140 mM	Kin. V. (mm^2/s)	3.883	3.104	2.554	2.125	1.847	1.575	1.370	1.224	1.157
	Specific V.	0.938	0.884	0.847	0.811	0.783	0.750	0.730	0.715	0.705
120 mM	Kin. V. (mm^2/s)	3.544	2.868	2.316	1.972	1.688	1.476	1.299	1.144	1.086
	Specific V.	0.769	0.735	0.690	0.663	0.637	0.630	0.620	0.603	0.601
100 mM	Kin. V. (mm^2/s)	3.437	2.725	2.206	1.843	1.592	1.378	1.230	1.091	1.039
	Specific V.	0.715	0.654	0.595	0.547	0.544	0.531	0.527	0.517	0.513
80 mM	Kin. V. (mm^2/s)	3.408	2.651	2.160	1.788	1.522	1.312	1.165	1.025	0.969
	Specific V.	0.675	0.609	0.562	0.511	0.475	0.453	0.446	0.436	0.429
60 mM	Kin. V. (mm^2/s)	3.141	2.487	2.020	1.719	1.420	1.225	1.103	0.979	0.929
	Specific V.	0.568	0.510	0.461	0.419	0.377	0.356	0.366	0.369	0.369
50 mM	Kin. V. (mm^2/s)	2.945	2.361	2.115	1.806	1.546	1.193	1.066	0.963	0.903
	Specific V.	0.470	0.433	0.529	0.527	0.499	0.321	0.330	0.336	0.331
40 mM	Kin. V. (mm^2/s)	2.752	2.195	1.821	1.513	1.283	1.095	0.988	0.864	0.821
	Specific V.	0.374	0.332	0.295	0.285	0.244	0.227	0.224	0.211	0.211
30 mM	Kin. V. (mm^2/s)	2.585	2.075	1.720	1.450	1.240	1.085	0.950	0.844	0.798
	Specific V.	0.290	0.259	0.244	0.225	0.202	0.192	0.182	0.182	0.177

Table S2: Viscosity measurement of 2 in DMSO.

	Temp (°C)	25	35	45	55	65	75	85	95	100
200 mM	Kin. V. (mm²/s)	4.891	3.950	3.184	2.666	2.274	1.944	1.712	1.500	1.409
	Specific V.	1.441	1.398	1.302	1.253	1.205	1.152	1.143	1.101	1.077
180 mM	Kin. V. (mm²/s)	4.443	3.412	2.752	2.296	1.952	1.689	1.485	1.314	1.246
	Specific V.	1.218	1.071	0.990	0.940	0.893	0.870	0.859	0.842	0.836
160 mM	Kin. V. (mm²/s)	4.071	3.265	2.681	2.251	1.927	1.672	1.465	1.300	1.234
	Specific V.	1.032	0.982	0.939	0.902	0.868	0.851	0.833	0.822	0.819
140 mM	Kin. V. (mm²/s)	3.883	3.104	2.554	2.125	1.820	1.575	1.373	1.224	1.157
	Specific V.	0.938	0.884	0.847	0.796	0.765	0.744	0.719	0.715	0.705
120 mM	Kin. V. (mm²/s)	3.544	2.868	2.366	1.972	1.696	1.476	1.299	1.144	1.083
	Specific V.	0.769	0.741	0.711	0.667	0.644	0.635	0.626	0.603	0.597
100 mM	Kin. V. (mm²/s)	3.437	2.689	2.178	1.843	1.592	1.395	1.230	1.091	1.039
	Specific V.	0.715	0.632	0.575	0.557	0.544	0.544	0.539	0.529	0.531
80 mM	Kin. V. (mm²/s)	3.408	2.651	2.160	1.788	1.522	1.312	1.165	1.025	0.969
	Specific V.	0.701	0.609	0.562	0.509	0.475	0.453	0.458	0.436	0.429
60 mM	Kin. V. (mm²/s)	3.137	2.581	2.168	1.855	1.617	1.416	1.248	1.089	1.015
	Specific V.	0.565	0.566	0.567	0.568	0.568	0.568	0.562	0.526	0.497
50 mM	Kin. V. (mm²/s)	2.856	2.324	1.941	1.650	1.429	1.247	1.097	0.965	0.905
	Specific V.	0.425	0.411	0.403	0.394	0.386	0.381	0.373	0.352	0.335
40 mM	Kin. V. (mm²/s)	2.578	2.070	1.715	1.446	1.243	1.079	0.948	0.841	0.796
	Specific V.	0.287	0.256	0.240	0.222	0.205	0.195	0.186	0.179	0.174
30 mM	Kin. V. (mm²/s)	2.626	2.099	1.718	1.438	1.244	1.084	0.953	0.848	0.808
	Specific V.	0.310	0.274	0.243	0.215	0.206	0.200	0.192	0.189	0.191

Table S3: Viscosity measurement of 2 in chloroform.

	Temp (°C)	25	30	35	40	45	50
100 mM	Kin. V. (mm²/s)	0.718	0.689	0.662	0.635	0.611	0.590
	Specific V.	0.879	0.881	0.882	0.879	0.864	0.856
80 mM	Kin. V. (mm²/s)	0.628	0.613	0.593	0.572	0.557	0.542
	Specific V.	0.643	0.674	0.684	0.693	0.698	0.705
60 mM	Kin. V. (mm²/s)	0.537	0.537	0.522	0.509	0.502	0.493
	Specific V.	0.407	0.465	0.485	0.506	0.532	0.553
40 mM	Kin. V. (mm²/s)	0.455	0.437	0.420	0.403	0.388	0.376
	Specific V.	0.195	0.196	0.196	0.195	0.188	0.185

Table S4: Viscosity measurement of 2 in toluene.

	Temp (°C)	25	35	45	55	65	75	85	95	100
100 mM	Kin. V. (mm²/s)	1.231	1.173	1.100	1.018	0.920	0.823	0.737	0.663	0.631
	Specific V.	0.784	0.768	0.752	0.736	0.720	0.712	0.704	0.696	0.688
80 mM	Kin. V. (mm²/s)	1.111	1.069	1.025	0.972	0.899	0.814	0.735	0.665	0.636
	Specific V.	0.610	0.612	0.632	0.657	0.681	0.692	0.700	0.702	0.706
60 mM	Kin. V. (mm²/s)	0.886	0.864	0.852	0.833	0.792	0.727	0.663	0.604	0.580
	Specific V.	0.283	0.303	0.356	0.421	0.481	0.512	0.533	0.547	0.551
40 mM	Kin. V. (mm²/s)	0.849	0.803	0.747	0.686	0.615	0.548	0.489	0.438	0.415
	Specific V.	0.228	0.206	0.188	0.168	0.151	0.139	0.126	0.118	0.108

Table S5: Viscosity measurement of 3 in Nynas NS8.

	Temp (°C)	25	35	45	55	65	75	85	95	100
60 mM	Kin. V. (mm²/s)	17.348	12.045	8.921	6.802	5.311	4.256	3.486	2.930	2.714
	Specific V.	0.335	0.355	0.371	0.388	0.403	0.415	0.425	0.430	0.432

Table S6: Viscosity measurement of 3 in Nynas NS8.

	Temp (°C)	25	35	45	55	65	75	85	95	100
100 mM	Kin. V. (mm²/s)	15.059	10.125	7.312	5.440	4.159	3.272	2.640	2.194	2.024
	Specific V.	0.159	0.139	0.124	0.110	0.098	0.088	0.079	0.071	0.068
80 mM	Kin. V. (mm²/s)	14.322	9.704	7.053	5.278	4.056	3.206	2.598	2.167	2.002
	Specific V.	0.102	0.092	0.084	0.077	0.071	0.066	0.062	0.058	0.056
60 mM	Kin. V. (mm²/s)	13.751	9.371	6.843	5.143	3.968	3.147	2.558	2.139	1.979
	Specific V.	0.060	0.056	0.053	0.051	0.049	0.048	0.047	0.046	0.046
60 mM	Kin. V. (mm²/s)	13.361	9.121	6.669	5.017	3.873	3.074	2.500	2.092	1.935
	Specific V.	0.028	0.026	0.025	0.024	0.023	0.022	0.022	0.021	0.021
40 mM	Kin. V. (mm²/s)	13.161	8.996	6.583	4.955	3.827	3.040	2.473	2.070	1.914
	Specific V.	0.013	0.012	0.012	0.011	0.011	0.011	0.010	0.010	0.010

Table S7: Viscosity measurement of 3 in Nexbase 3020.

	Temp (°C)	25	35	45	55	65	75	85	95	100
40 mM	Kin. V. (mm²/s)	14.356	9.586	6.639	4.781	3.625	2.868	2.349	1.976	1.771
	Specific V.	13.297	2.876	-2.042	-5.988	-8.650	-11.124	-12.504	-12.713	-12.875

Table S8. Viscosity measurement of 3 in toluene.

	Temp (°C)	25	35	45	55	65	75	85	95	100
100 mM	Kin. V. (mm²/s)	1.690	1.341	1.121	0.955	0.818	0.705	0.614	0.542	0.514
	Specific V.	1.474	1.042	0.803	0.645	0.545	0.481	0.434	0.402	0.388
80 mM	Kin. V. (mm²/s)	1.606	1.274	1.065	0.907	0.777	0.669	0.583	0.515	0.488
	Specific V.	1.351	0.940	0.713	0.563	0.468	0.407	0.362	0.331	0.319
60 mM	Kin. V. (mm²/s)	1.477	1.172	0.979	0.835	0.715	0.616	0.537	0.474	0.449
	Specific V.	1.140	0.767	0.559	0.423	0.337	0.281	0.241	0.212	0.201
40 mM	Kin. V. (mm²/s)	1.374	1.090	0.911	0.776	0.665	0.573	0.499	0.441	0.417
	Specific V.	1.011	0.660	0.465	0.337	0.256	0.204	0.166	0.139	0.128
20 mM	Kin. V. (mm²/s)	1.305	1.035	0.865	0.738	0.632	0.544	0.474	0.419	0.397
	Specific V.	0.911	0.577	0.392	0.270	0.193	0.143	0.107	0.082	0.072

Table S9: Viscosity measurement of 4 in DMSO.

	Temp (°C)	25	35	45	55	65	75	85	95	100
60 mM	Kin. V. (mm²/s)	2.760	2.245	1.864	1.576	1.358	1.176	1.023	0.915	0.863
	Specific V.	0.498	0.493	0.488	0.482	0.477	0.471	0.461	0.442	0.422

Table S10 Viscosity measurement of 4 in chloroform.

	Temp (°C)	25	30	35	40	45	50
60 mM	Kin. V. (mm²/s)	0.484	0.467	0.460	0.448	0.432	0.419
	Specific V.	0.366	0.405	0.427	0.445	0.457	0.470

Table S11: Viscosity measurement of 4 in toluene.

	Temp (°C)	25	35	45	55	65	75	85	95	100
60 mM	Kin. V. (mm²/s)	0.753	0.726	0.707	0.683	0.642	0.581	0.524	0.471	0.447
	Specific V.	0.241	0.254	0.295	0.345	0.390	0.409	0.421	0.426	0.425

Table S12: Viscosity measurement of 4 in Nynas NS8.

	Temp (°C)	25	35	45	55	65	75	85	95	100
100 mM	Kin. V. (mm²/s)	25.371	16.173	11.218	8.064	5.977	4.583	3.610	2.939	2.682
	Specific V.	0.700	0.631	0.584	0.548	0.517	0.493	0.471	0.452	0.441
80 mM	Kin. V. (mm²/s)	19.183	12.739	9.135	6.767	5.153	4.048	3.259	2.703	2.488
	Specific V.	0.476	0.433	0.404	0.381	0.361	0.346	0.332	0.319	0.313
60 mM	Kin. V. (mm²/s)	17.085	11.719	8.641	6.570	5.125	4.115	3.377	2.848	2.640
	Specific V.	0.315	0.318	0.328	0.341	0.354	0.368	0.380	0.390	0.393
40 mM	Kin. V. (mm²/s)	15.289	10.328	7.496	5.606	4.306	3.404	2.758	2.300	2.123
	Specific V.	0.176	0.162	0.152	0.144	0.137	0.132	0.127	0.123	0.120
20 mM	Kin. V. (mm²/s)	13.963	9.518	6.956	5.232	4.039	3.208	2.609	2.183	2.019
	Specific V.	0.074	0.071	0.069	0.068	0.067	0.067	0.066	0.066	0.065

Table S13: Viscosity measurement of 4 in Nexbase 3020.

	Temp (°C)	25	35	45	55	65	75	85	95	100
60 mM	Kin. V. (mm²/s)	17.576	12.792	9.717	7.507	5.964	4.891	4.103	3.510	3.153
	Specific V.	0.387	0.407	0.434	0.461	0.485	0.508	0.528	0.551	0.562

7. DLS Measurements

7.1 DLS Measurement of the 1 in 60 mM DMSO

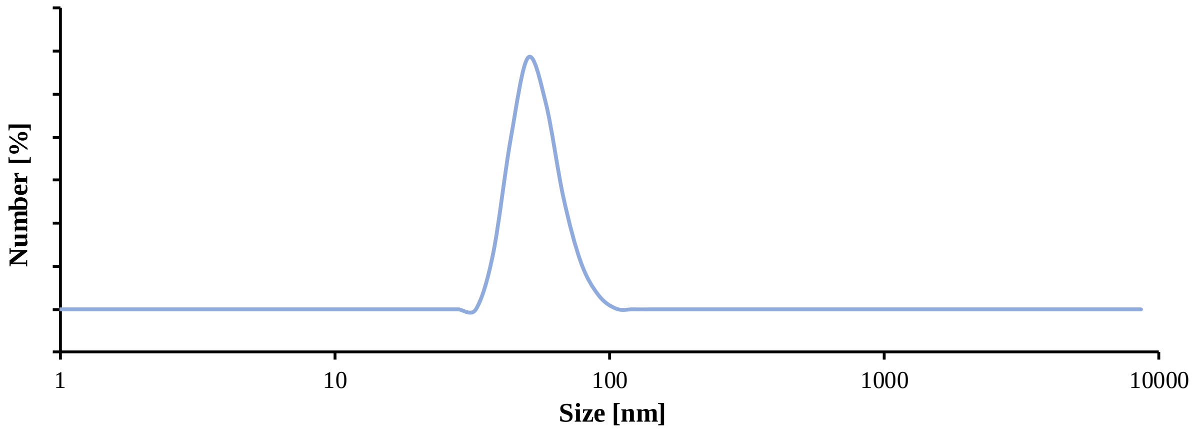

Figure S74: DLS measurement of 1 in 60 mM DMSO at 25 °C. Signal number peak: 100%.

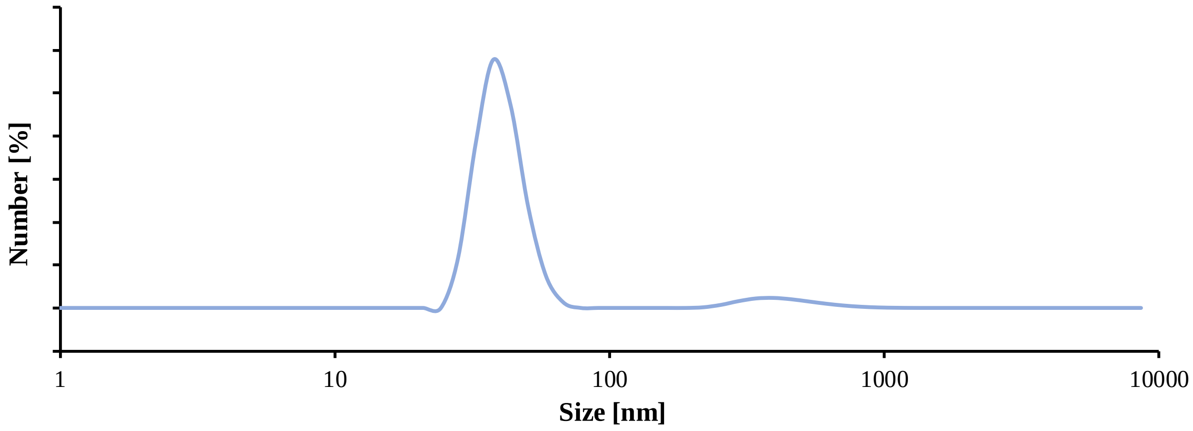

Figure S75: DLS measurement of 1 in 60 mM DMSO at 60 °C. Signal number peak left: 93.7% peak right 6.3%.

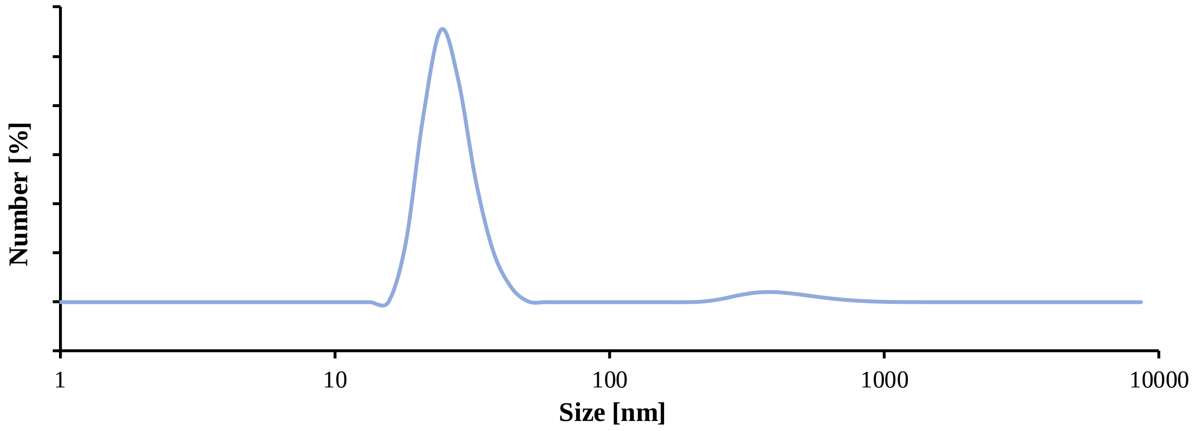

Figure S76: DLS measurement of 1 in 60 mM DMSO at 100 °C. Signal number peak left: 94.5% peak right 5.5%.

7.2 DLS Measurement of 2 in 60 mM DMSO

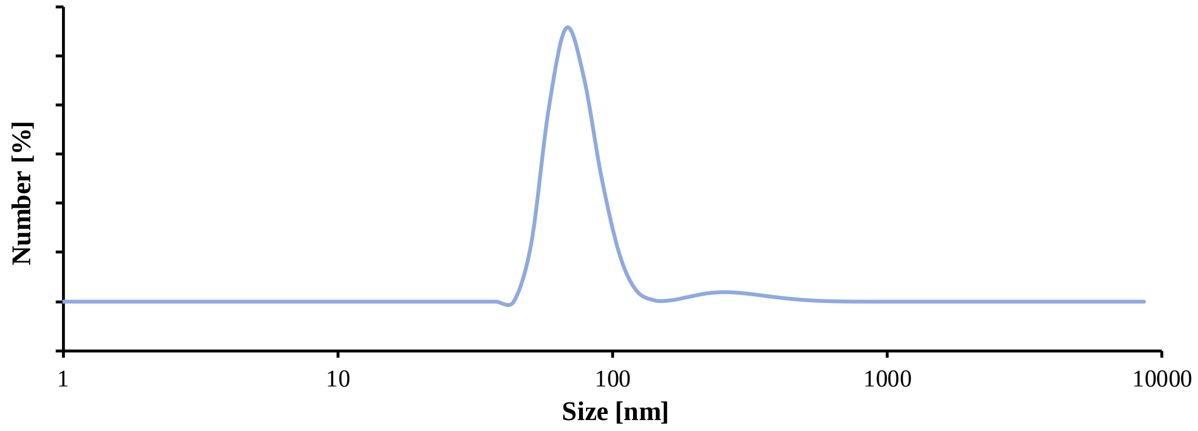

Figure S77: DLS measurement of 2 in 60 mM DMSO at 25 °C. Signal number peak left: 95.1% peak right 4.9%.

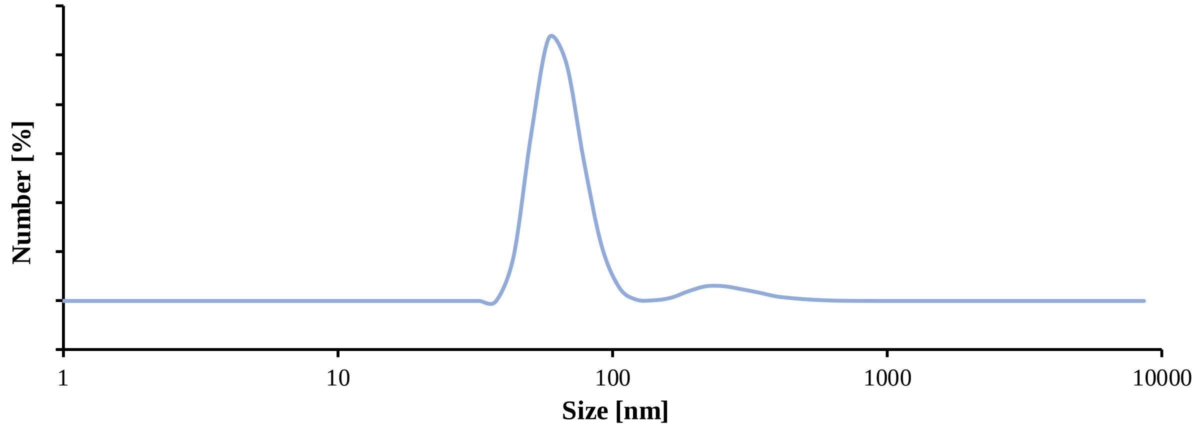

Figure S78: DLS measurement of 2 in 60 mM DMSO at 60 °C. Signal number peak left: 94.1% peak right 4.9%.

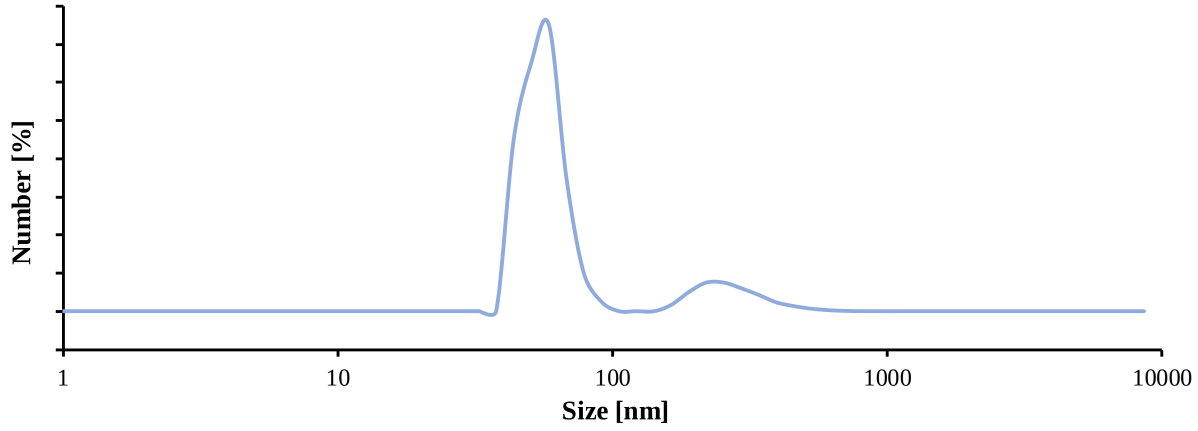

Figure S79: DLS measurement of 2 in 60 mM DMSO at 100 °C. Signal number peak left: 86.4% peak right 13.6%.

7.3 DLS Measurement of the 2 in 60 mM Chloroform

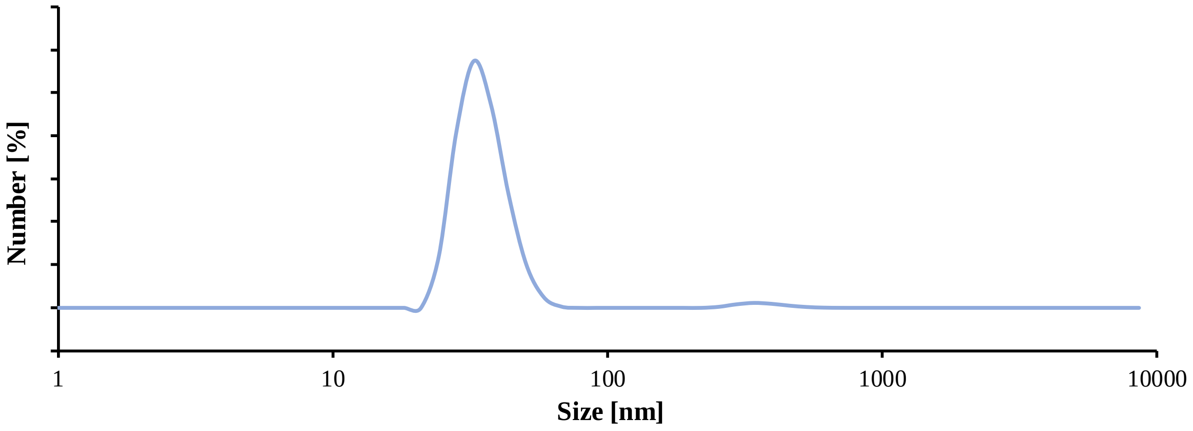

Figure S80: DLS measurement of 2generation in 60 mM chloroform at 25 °C. Signal number peak left: 98.0% peak right 2.0%.

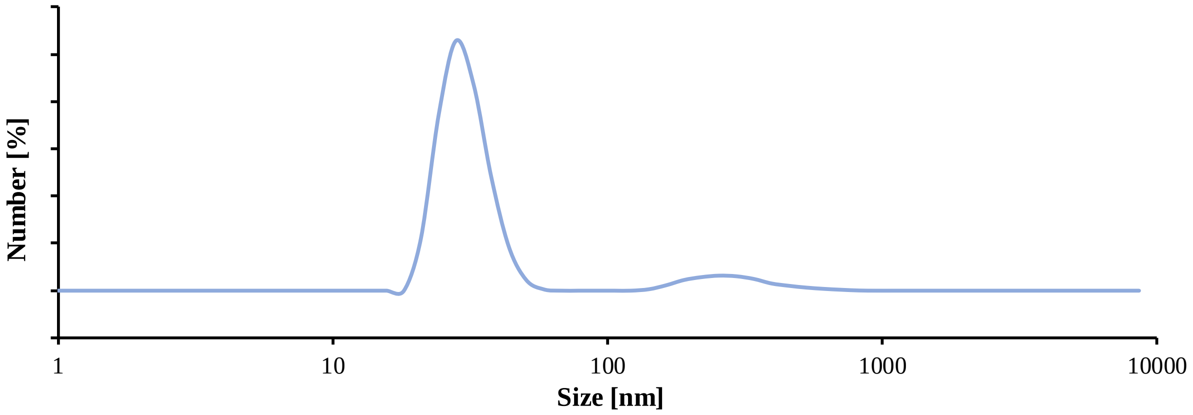

Figure S81: DLS measurement of 2 in 60 mM chloroform at 37 °C. Signal number peak left: 90.5% peak right 9.5%.

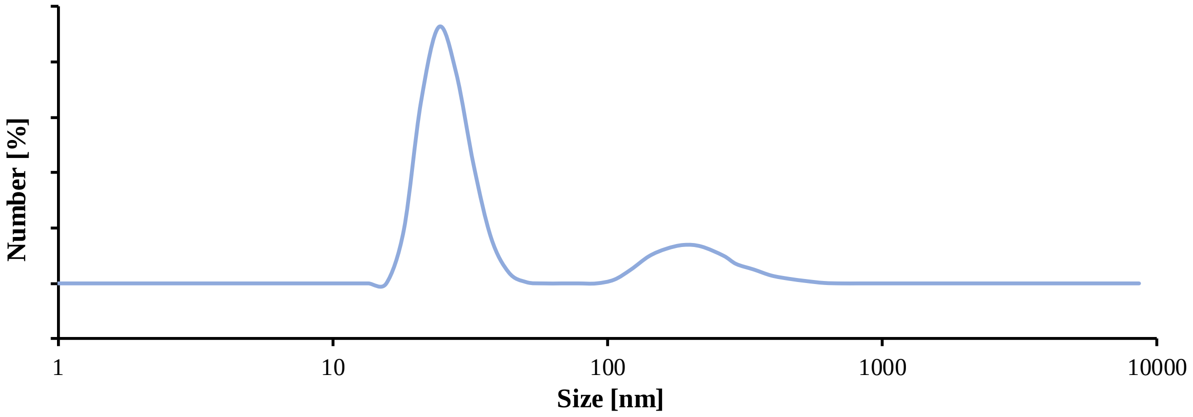

Figure S82: DLS measurement of 2 in 60 mM chloroform at 50 °C. Signal number peak left: 79.2% peak right 20.8%.

7.4 DLS Measurement of the 2 in toluene

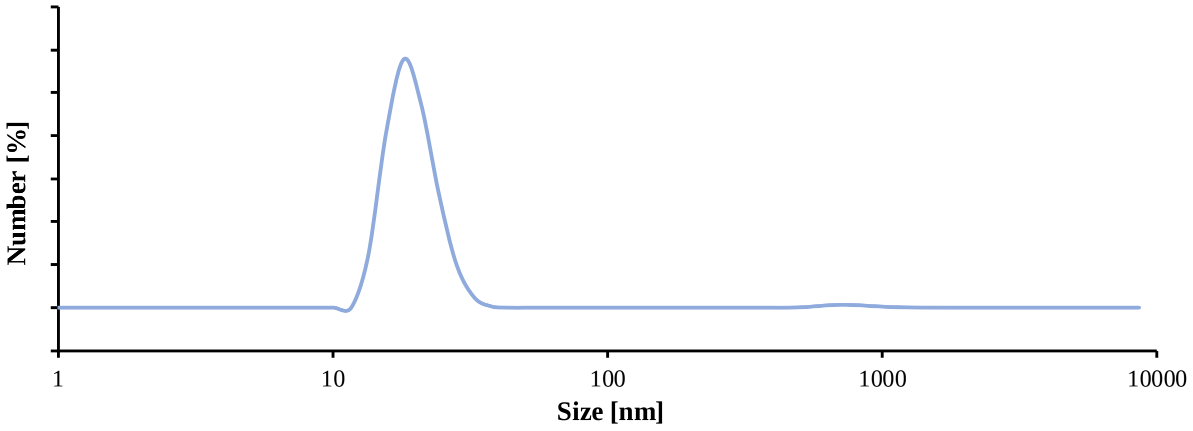

Figure S83: DLS measurement of 2 in 60 mM toluene at 25 °C. Signal number peak left: 98.9% peak right 1.1%.

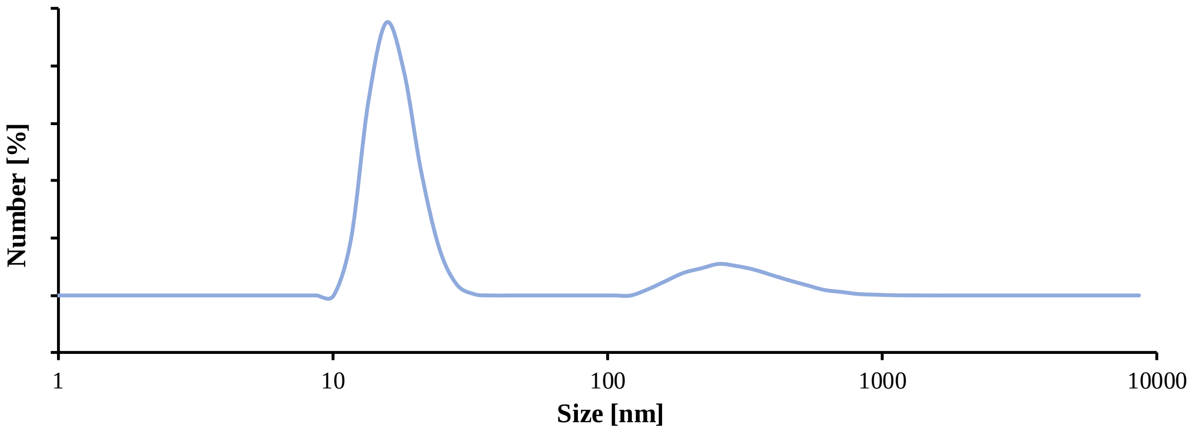

Figure S84: DLS measurement of 2 in 60 mM toluene at 60 °C. Signal number peak left: 81.3% peak right 18.7%.

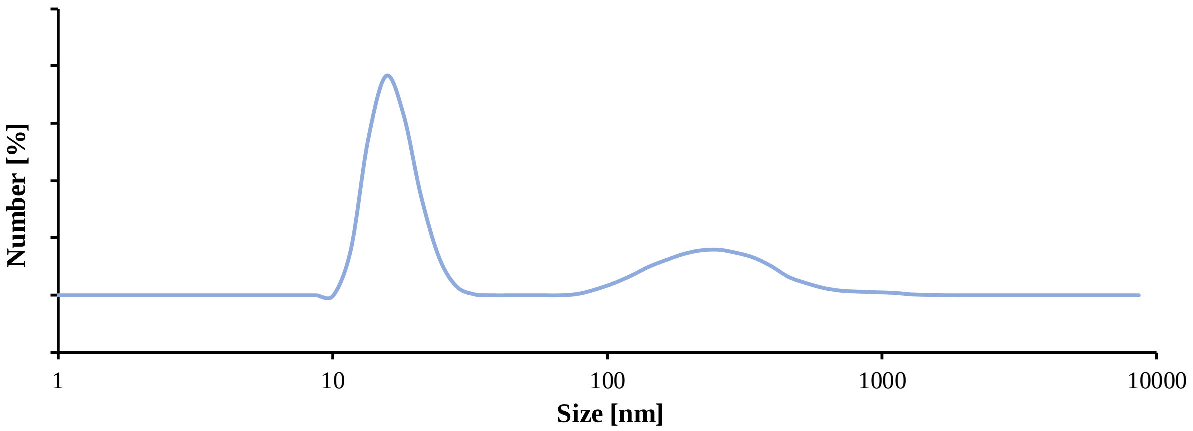

Figure S85: DLS measurement of 2 in 60 mM toluene at 100 °C. Signal number peak left: 65.4% peak right 34.6%.

8. Force field calculations

For molecular modelling studies, the Schrödinger Maestro Suite (11.8) was used. The structures were calculated using MacroModel and OPLS 2005 force field choosing chloroform as the solvent. Energy minimazation was performing using the PCRG-method (maximum 50000 steps, convergence threshold 0.01).

8.1 Force field calculation of 1

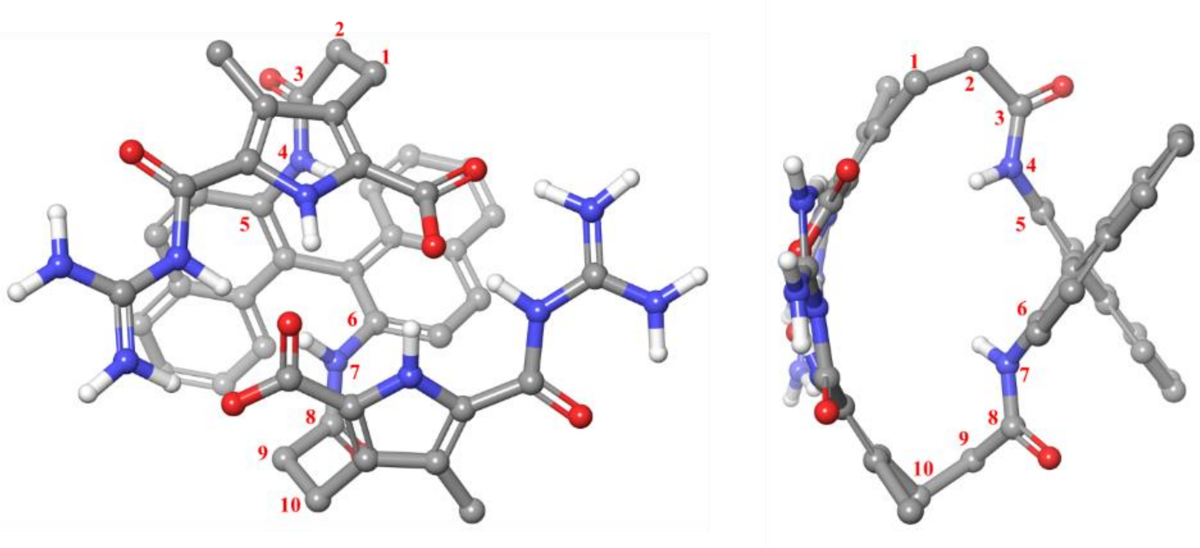

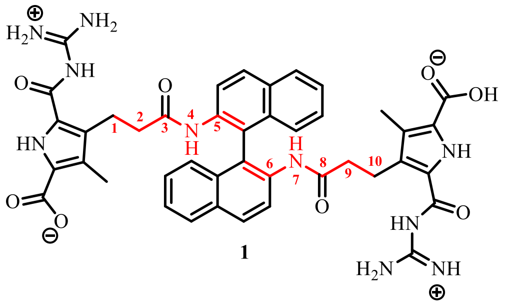

1

Figure S86: Force field calculation of the BINAM GCP system 1 to form cyclic structures (OPLS force field). Left: Front view. Right: Side view.

8.2 Force field calculation of 2

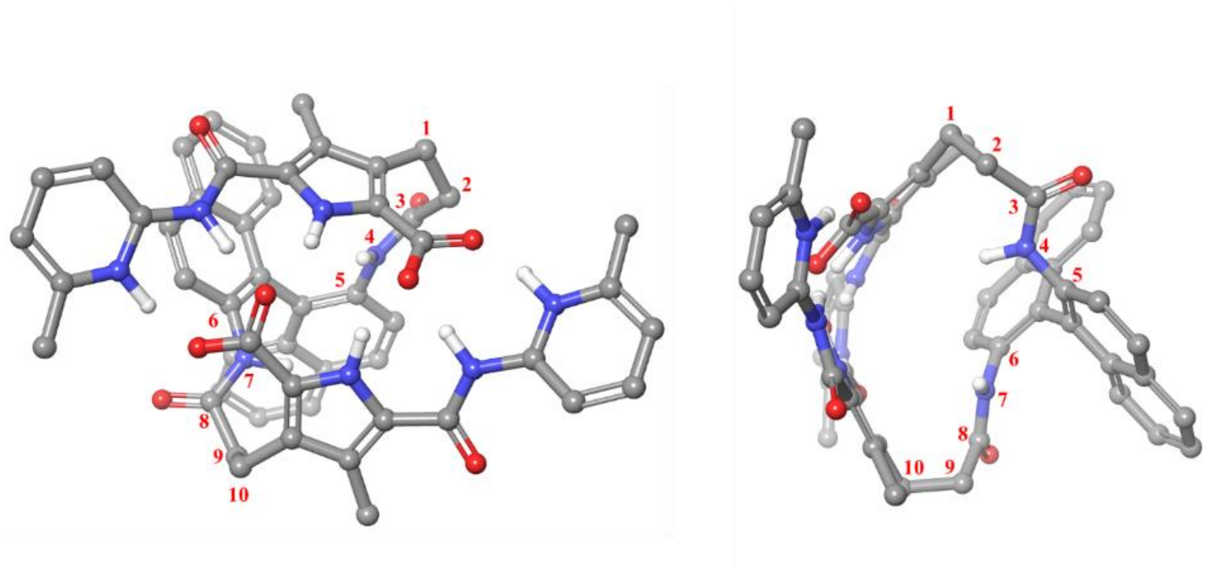

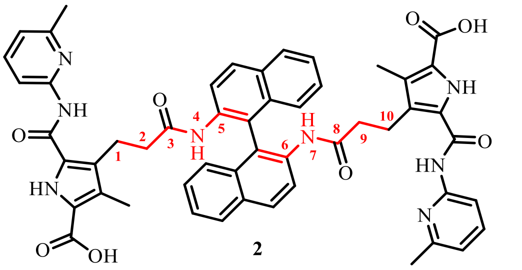

2

Figure S87: Force field calculation of the BINAM ACP system 2 to form cyclic structures (OPLS force field). Left: Front view. Right: Side view.

9. NMR-Spectra

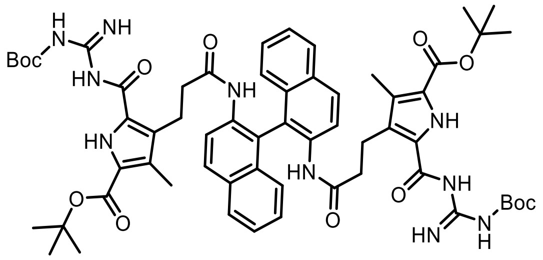

9

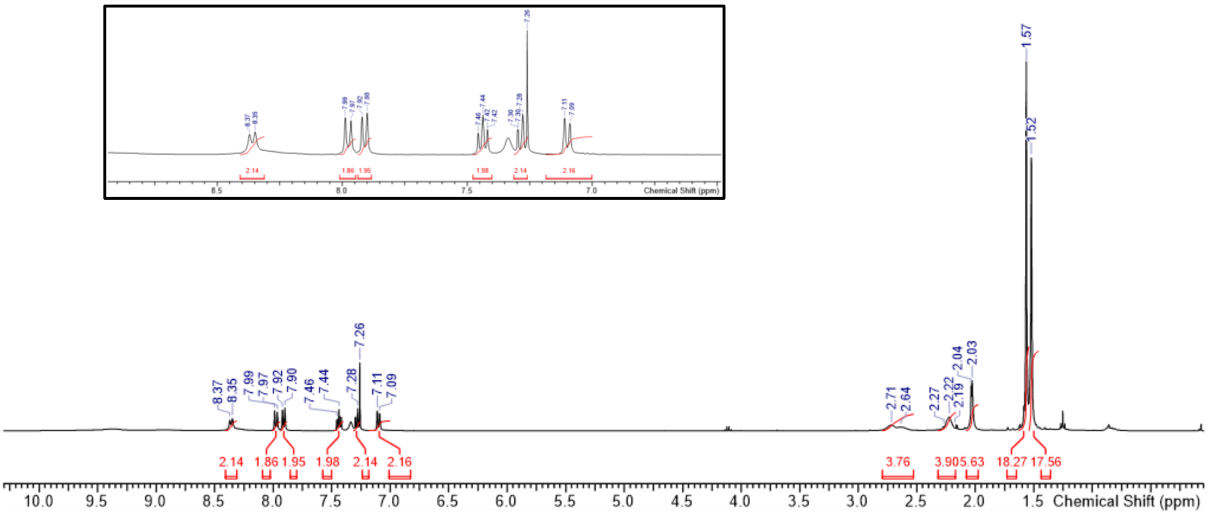

Figure S88: ^{1}H-NMR of 9. 400 MHz, Solvent: $CDCl_3$, 27 °C.

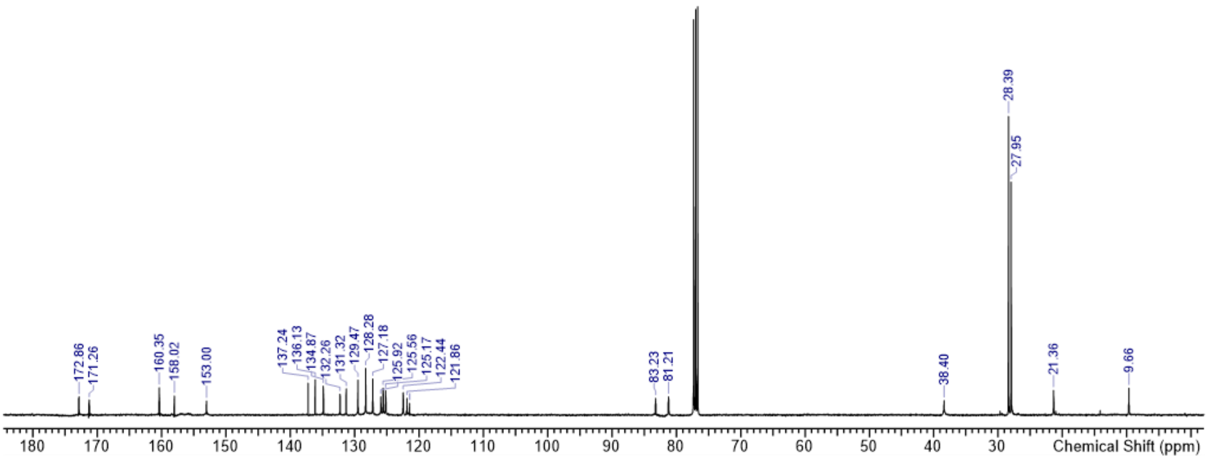

Figure S89: ^{13}C-NMR of 9. 151 MHz, Solvent: $CDCl_3$, 27 °C.

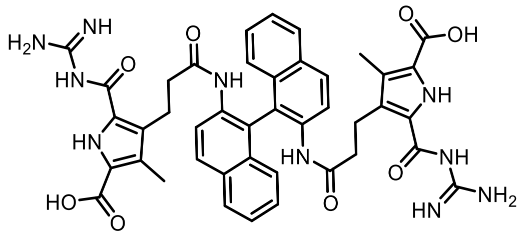

1

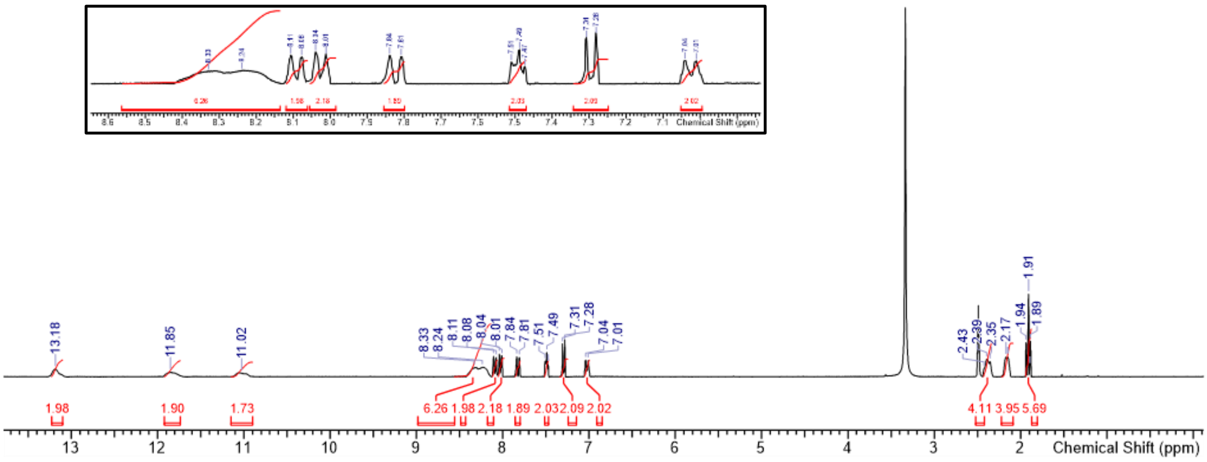

Figure S90: ^{1}H-NMR of 1. 300 MHz, Solvent: DMSO-d6, 27 °C.

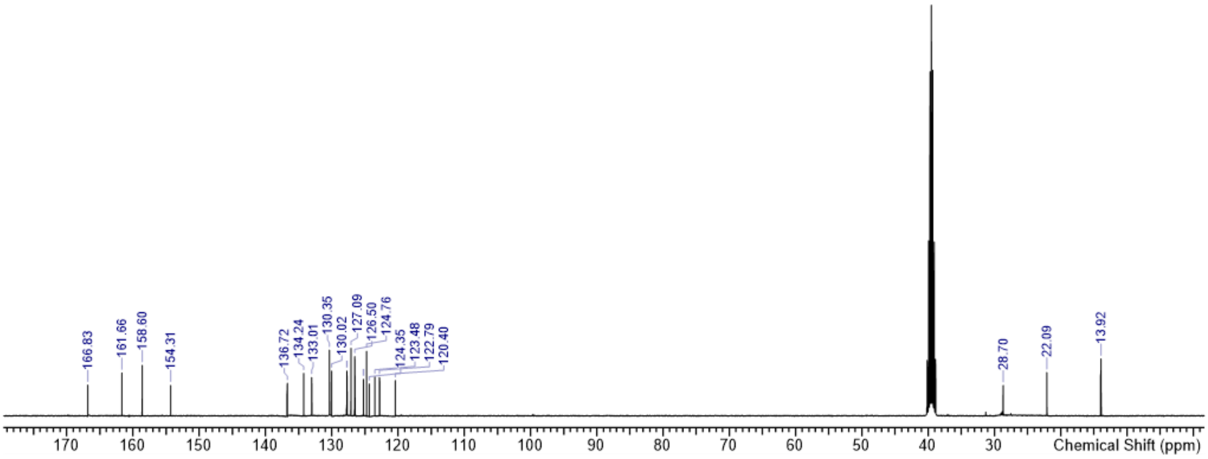

Figure S91: ^{13}C-NMR of 1. 151 MHz, Solvent: DMSO-d6, 27 °C.

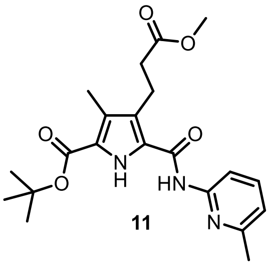

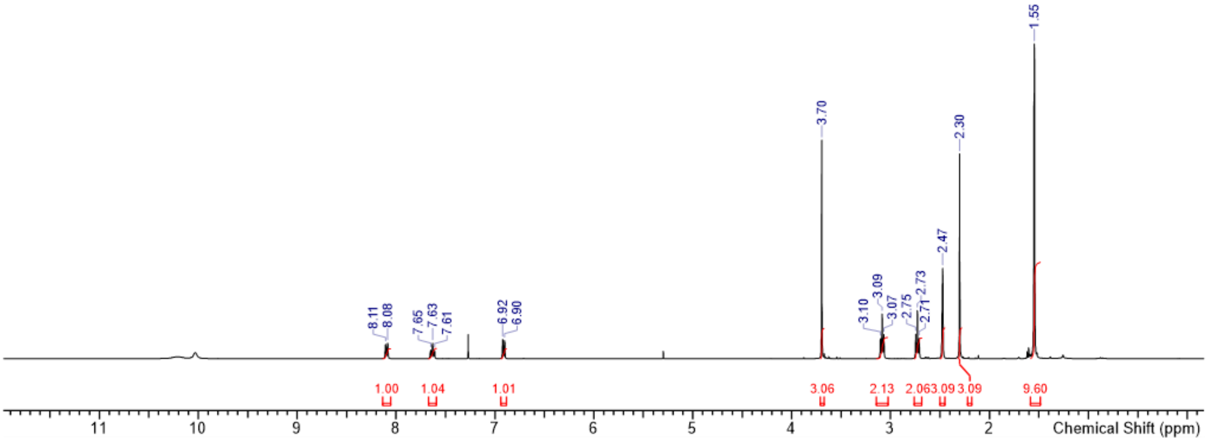

Figure S92: ^{1}H-NMR of 11. 400 MHz, Solvent: $CDCl_3$, 27 °C.

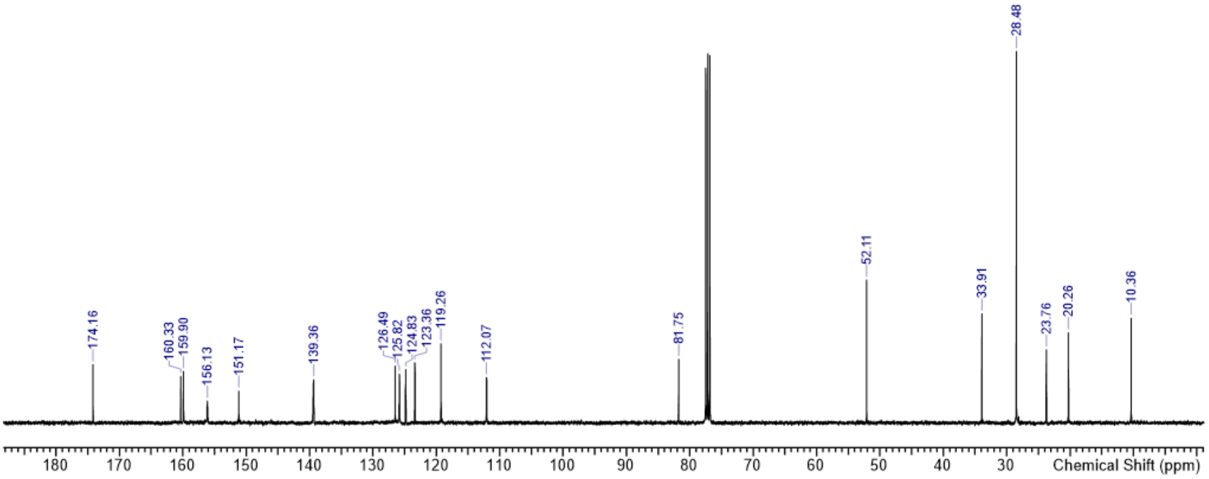

Figure S93: ^{13}C-NMR of 11. 151 MHz, Solvent: $CDCl_3$, 27 °C.

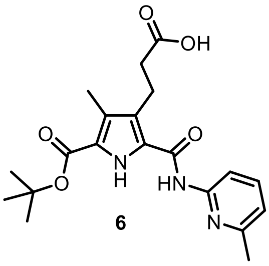

6

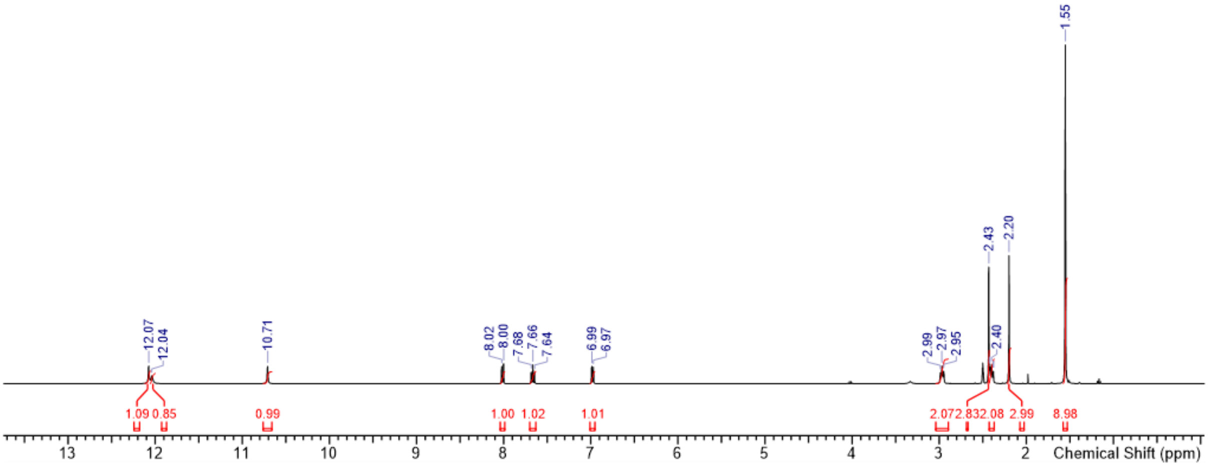

Figure S94: ^{1}H-NMR of 6. 400 MHz, Solvent: DMSO-d$_6$, 27 °C.

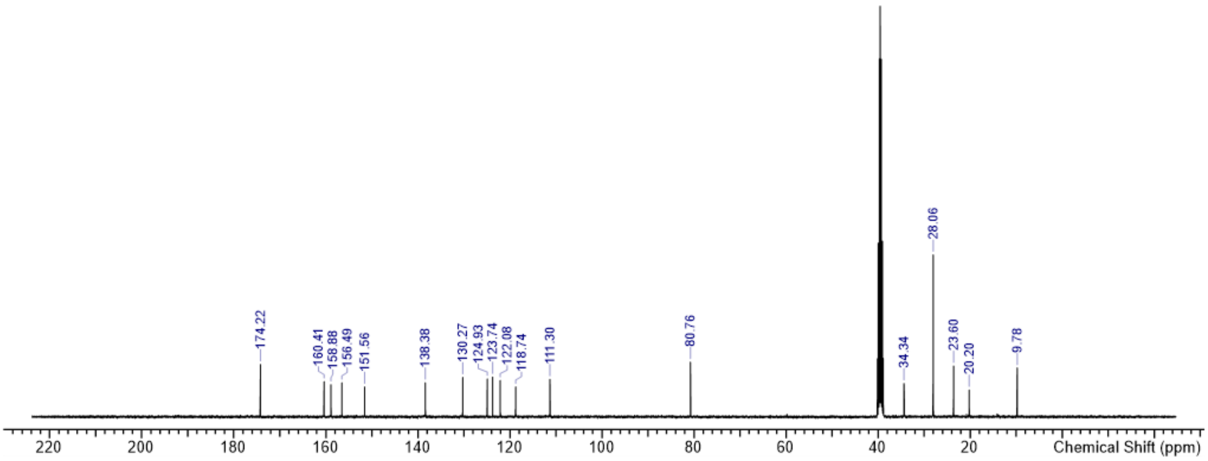

Figure S95: ^{13}C-NMR of 6. 151 MHz, Solvent: DMSO-d$_6$, 27 °C.

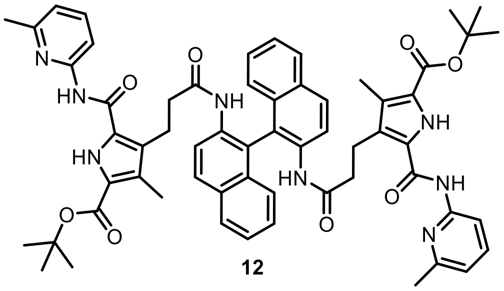

12

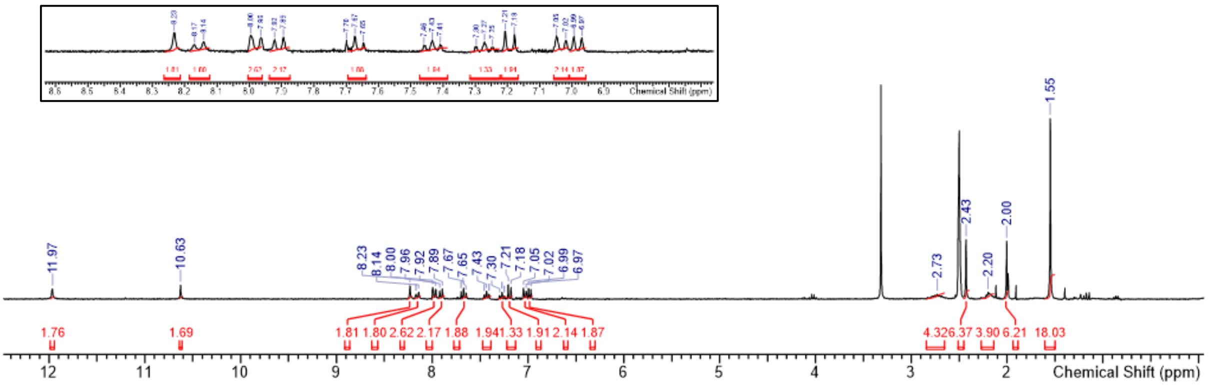

Figure S96: ^{1}H-NMR of 12. 300 MHz, Solvent: DMSO-d6, 27 °C.

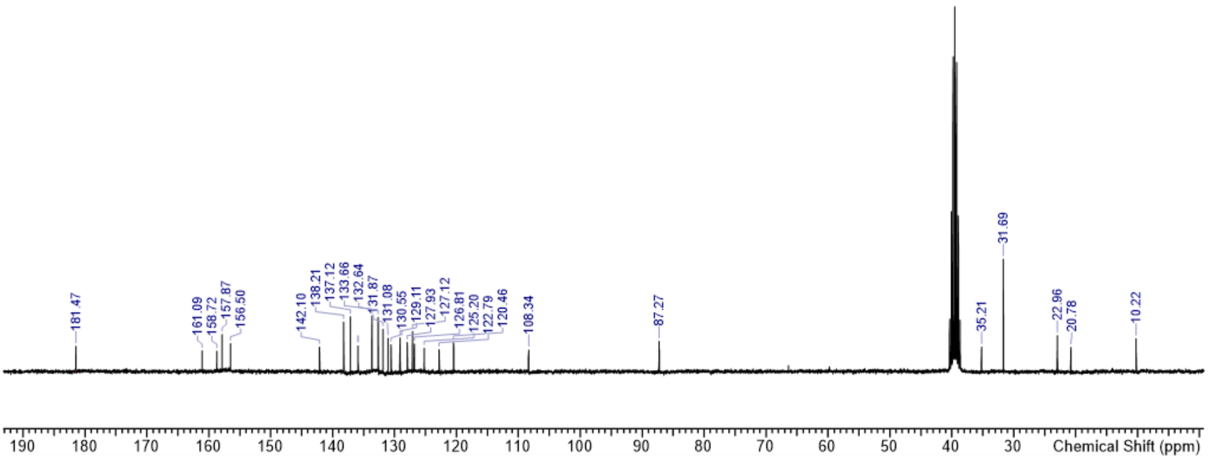

Figure S97: ^{13}C-NMR of 12. 75 MHz, Solvent: DMSO-d6, 27 °C.

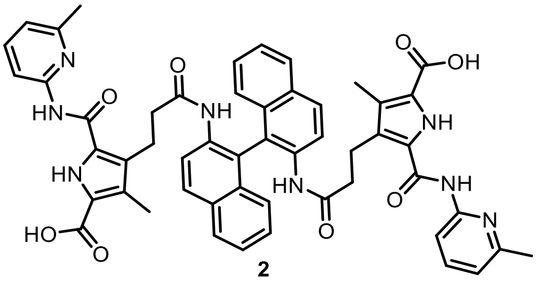

2

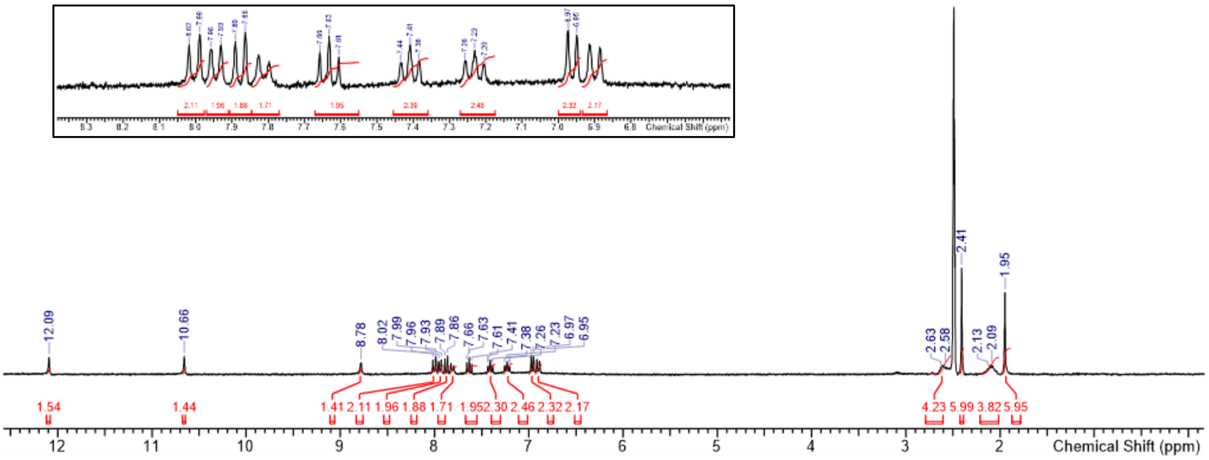

Figure S98: ^{1}H-NMR of 2. 300 MHz, Solvent: DMSO-d₆, 27 °C.

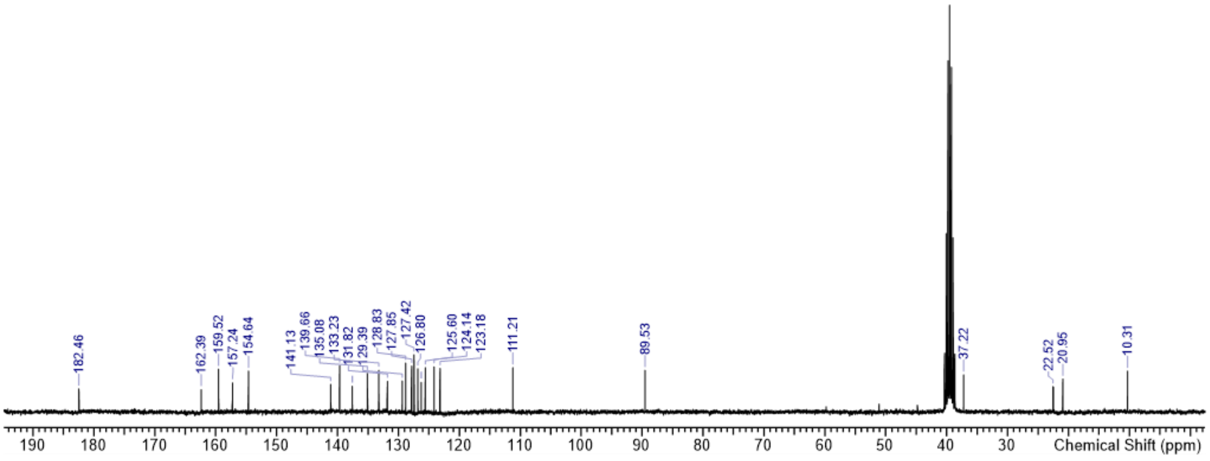

Figure S99: ^{13}C-NMR of 2. 75 MHz, Solvent: DMSO-d₆, 27 °C.

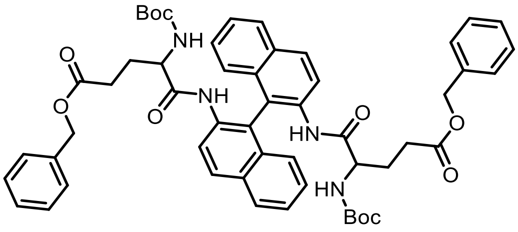

13

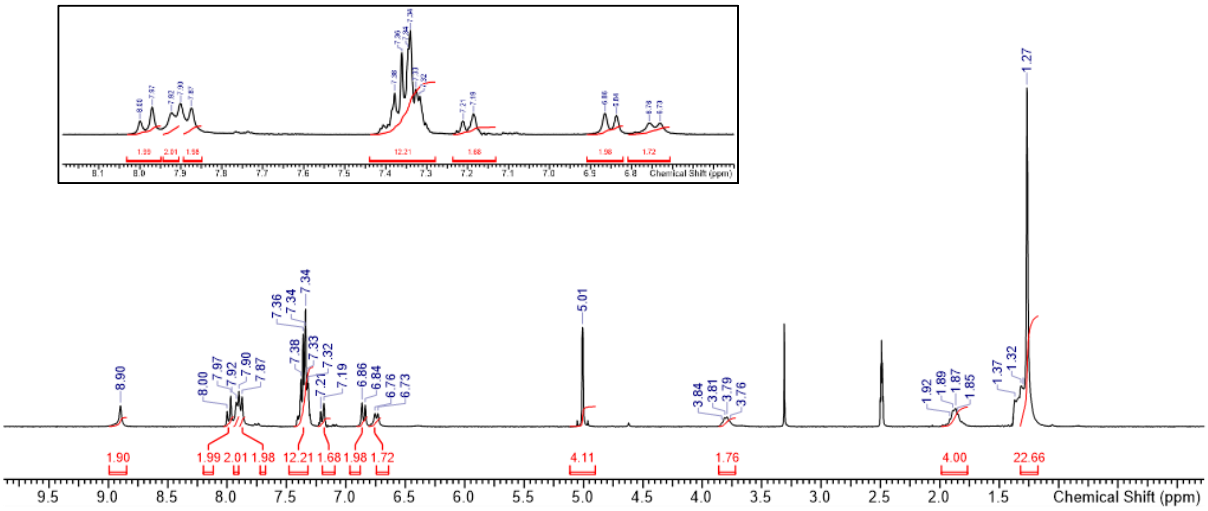

Figure S100: ^{1}H-NMR of 13. 300 MHz, Solvent: DMSO-d$_6$, 27 °C.

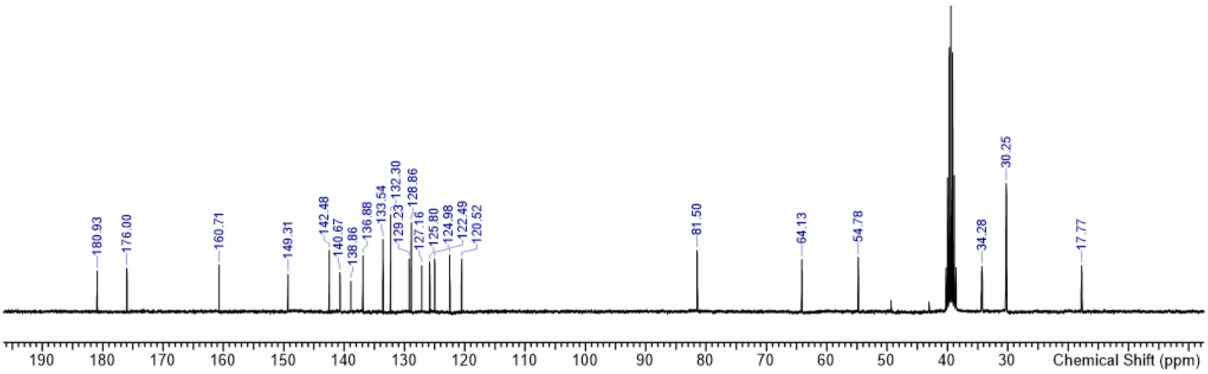

Figure S101: ^{13}C-NMR of 13. 75 MHz, Solvent: DMSO-d$_6$, 27 °C.

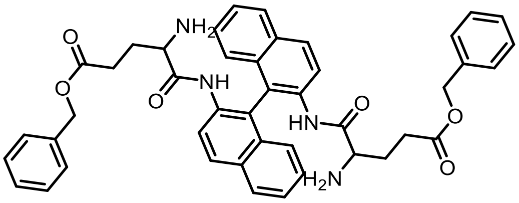

7

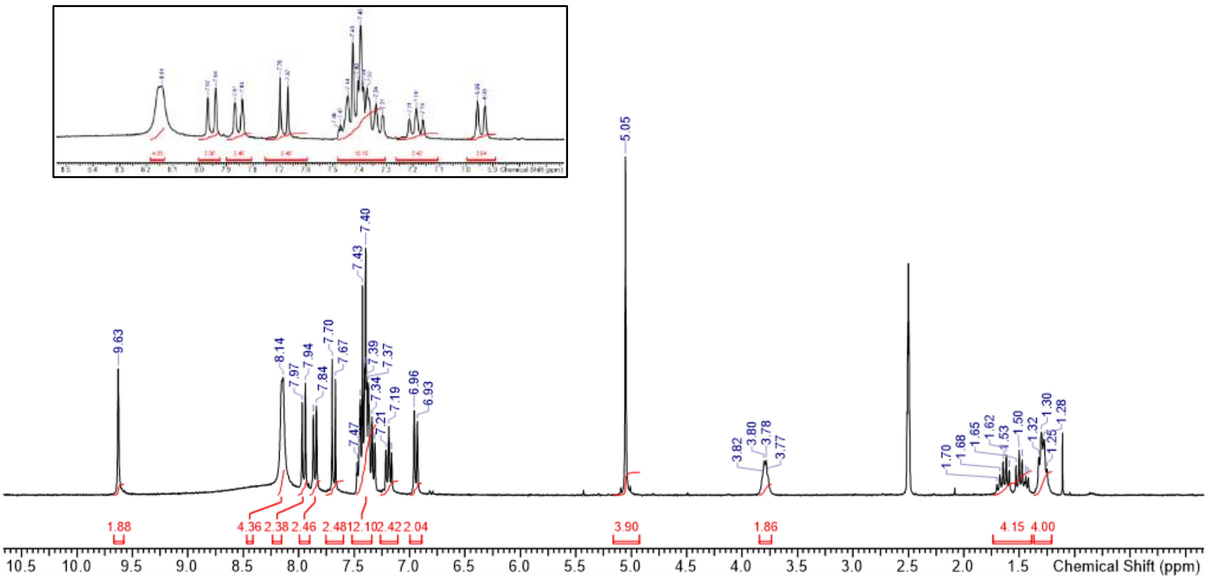

Figure S102: ^{1}H-NMR of 7. 300 MHz, Solvent: DMSO-d6, 27 °C.

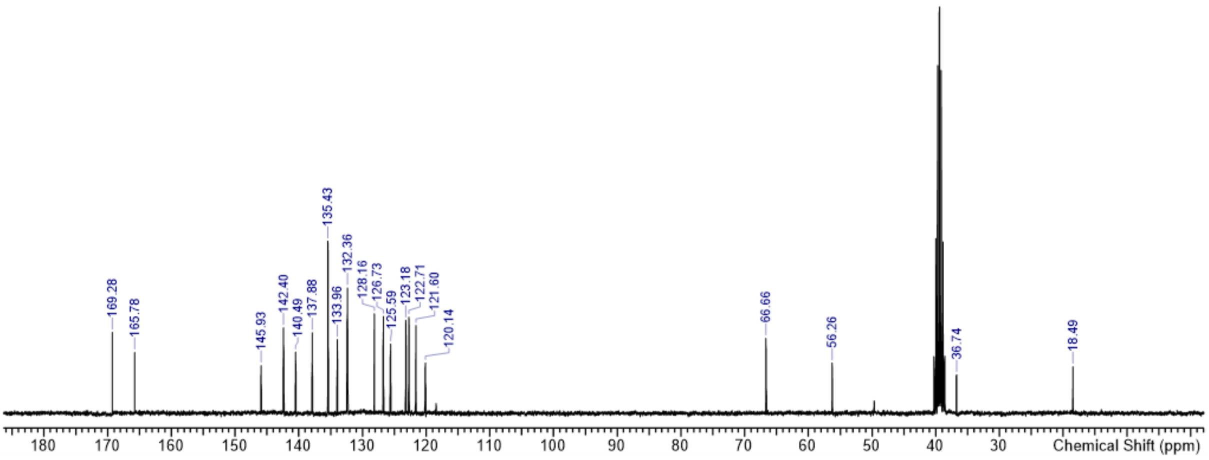

Figure S103: ^{13}C-NMR of 7. 75 MHz, Solvent: DMSO-d6, 27 °C.

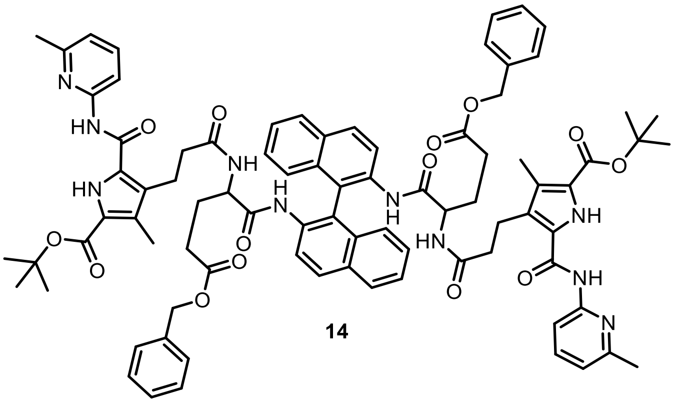

14

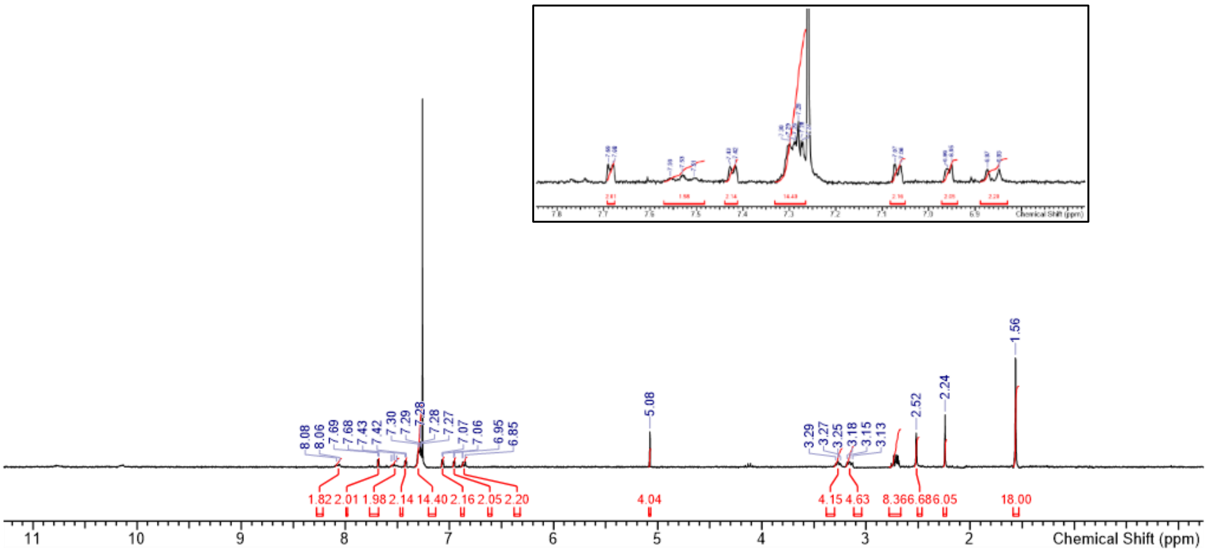

Figure S104: ^{1}H-NMR of 14. 300 MHz, Solvent: CDCl3, 27 °C.

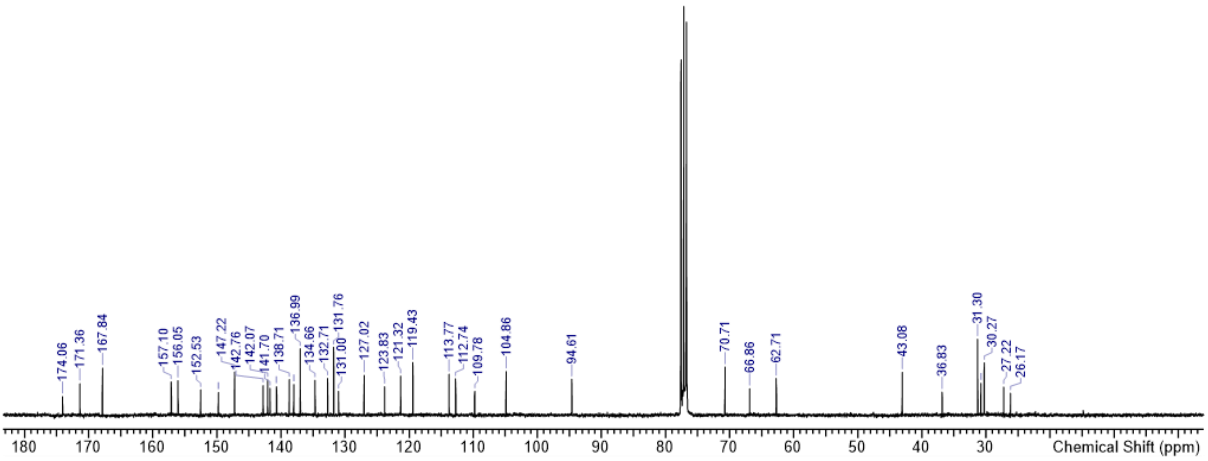

Figure S105: ^{13}C-NMR of 14. 75 MHz, Solvent: CDCl3, 27 °C.

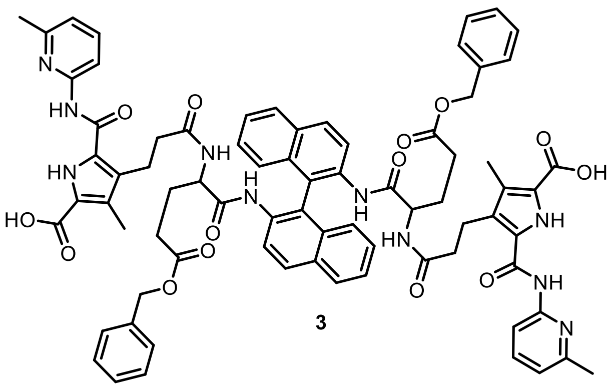

3

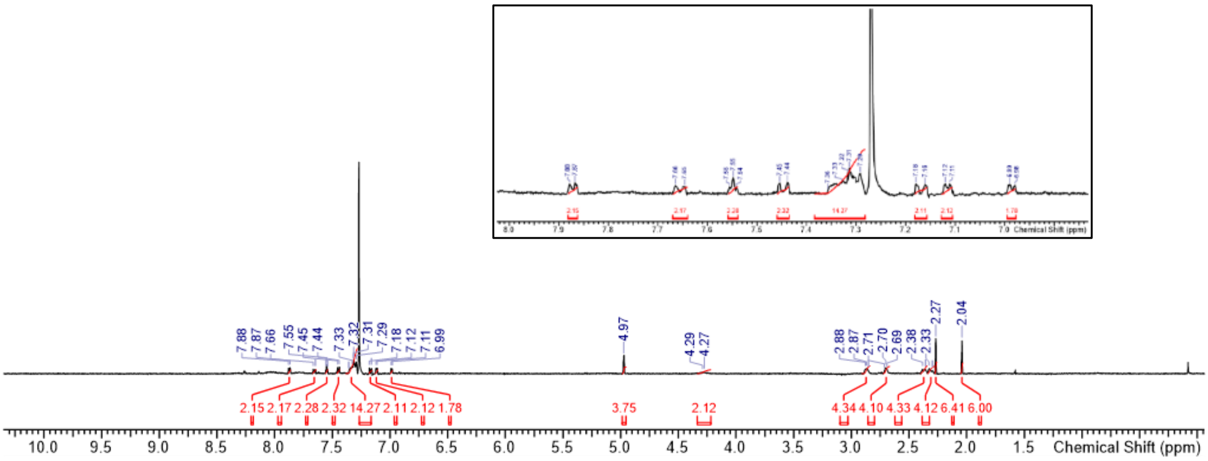

Figure S106: ^{1}H-NMR of 3. 300 MHz, Solvent: $CDCl_3$, 27 °C.

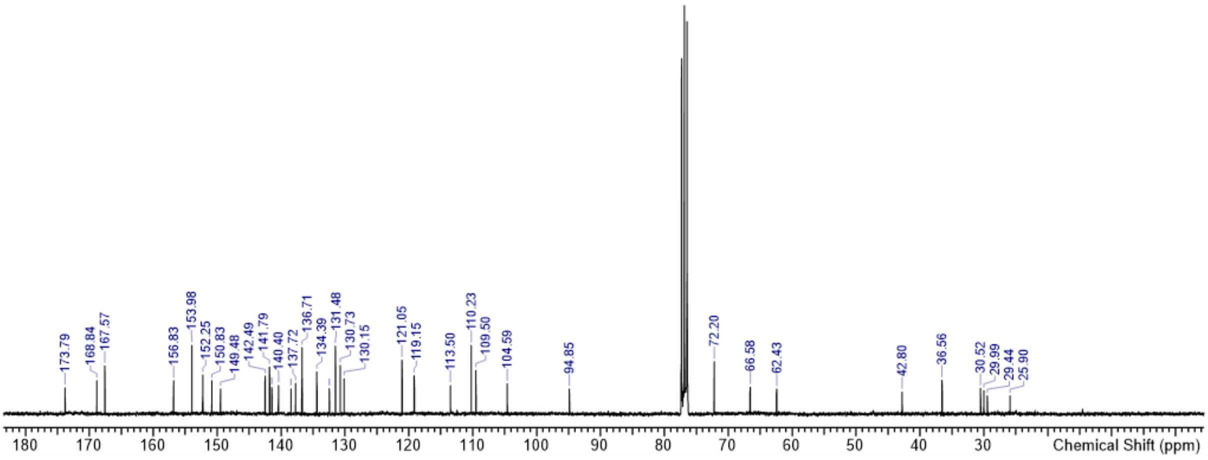

Figure S107: ^{13}C-NMR of 3. 75 MHz, Solvent: $CDCl_3$, 27 °C.

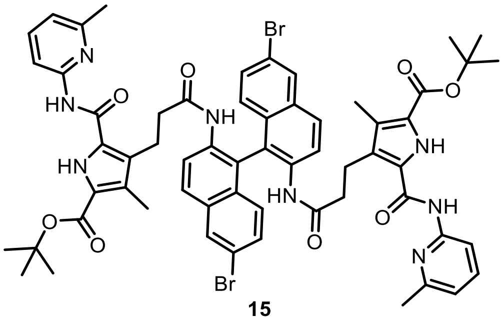

15

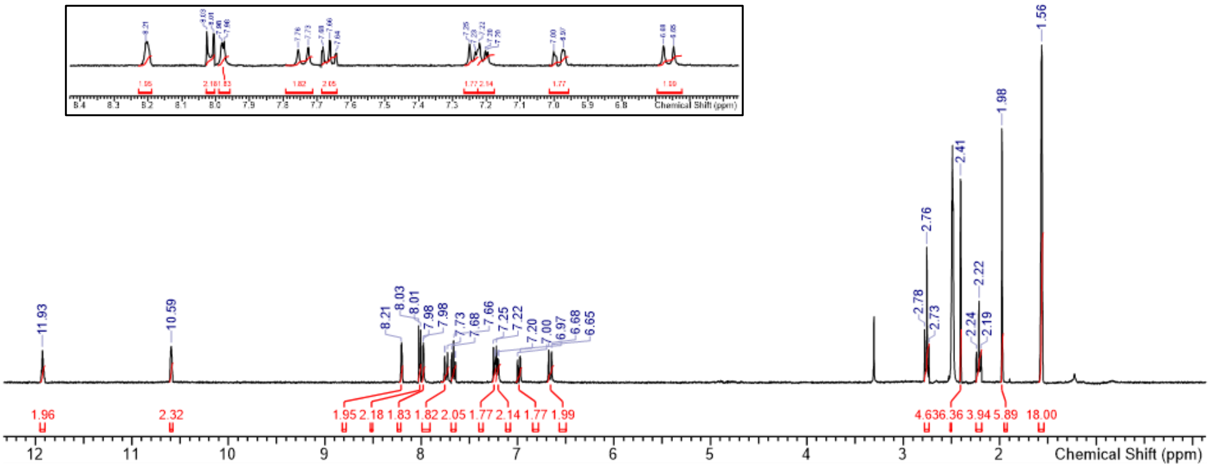

Figure S108: ¹H-NMR of 15. 400 MHz, Solvent: DMSO-d₆, 27 °C.

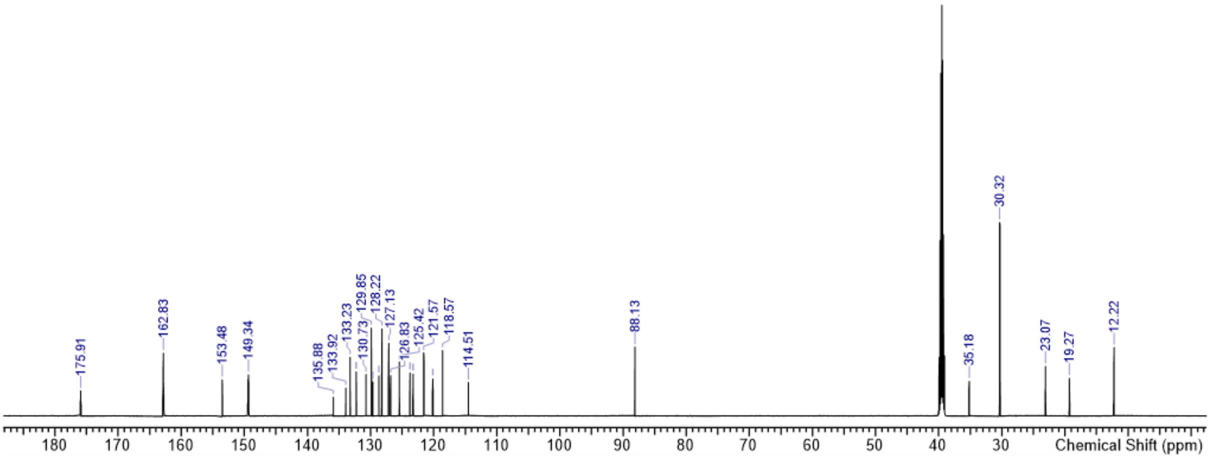

Figure S109: ¹³C-NMR of 15. 151 MHz, Solvent: DMSO-d₆, 27 °C.

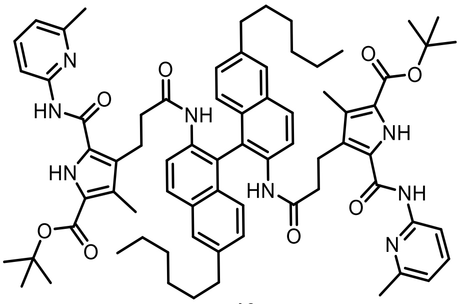

16

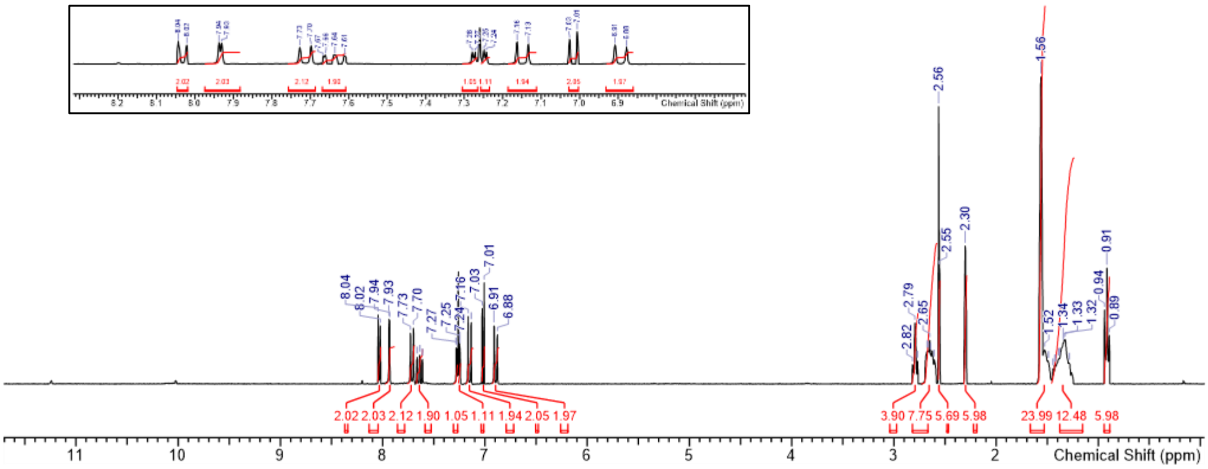

Figure S110: ^{1}H-NMR of 16. 400 MHz, Solvent: $CDCl_3$, 27 °C.

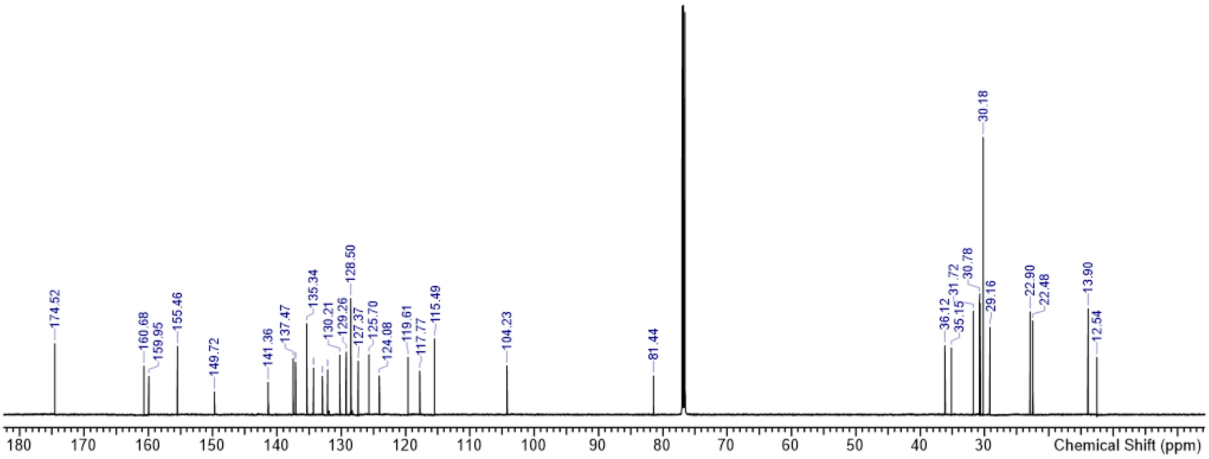

Figure S111: ^{13}C-NMR of 16. 151 MHz, Solvent: $CDCl_3$, 27 °C.

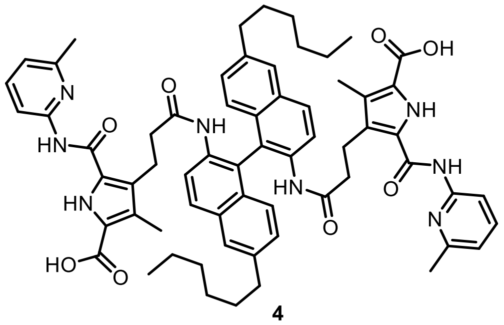

4

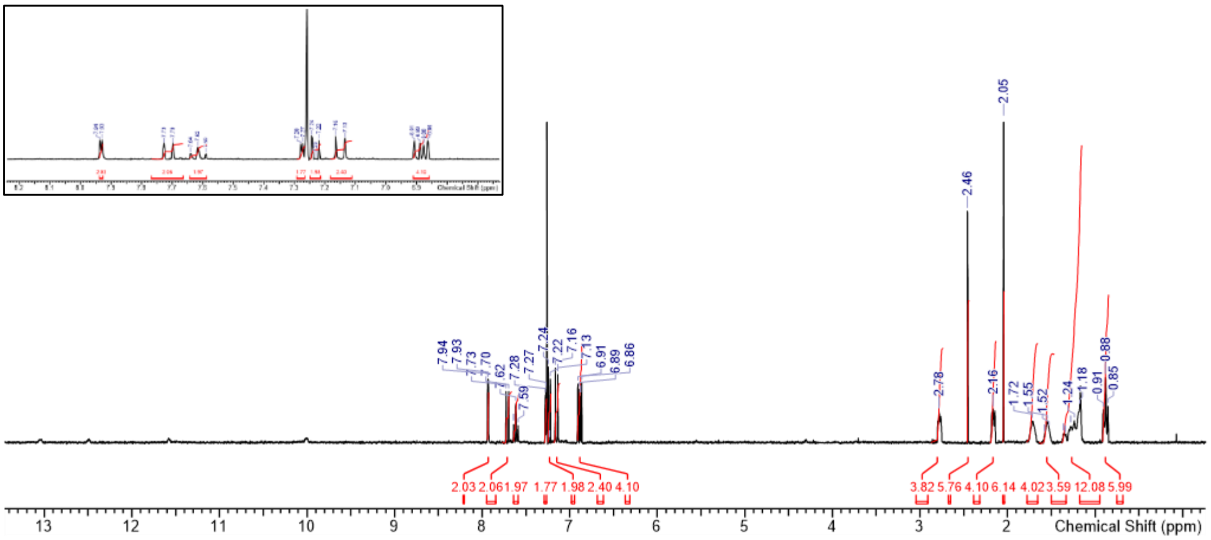

Figure S112: ^{1}H-NMR of 4. 400 MHz, Solvent: $CDCl_3$, 27 °C.

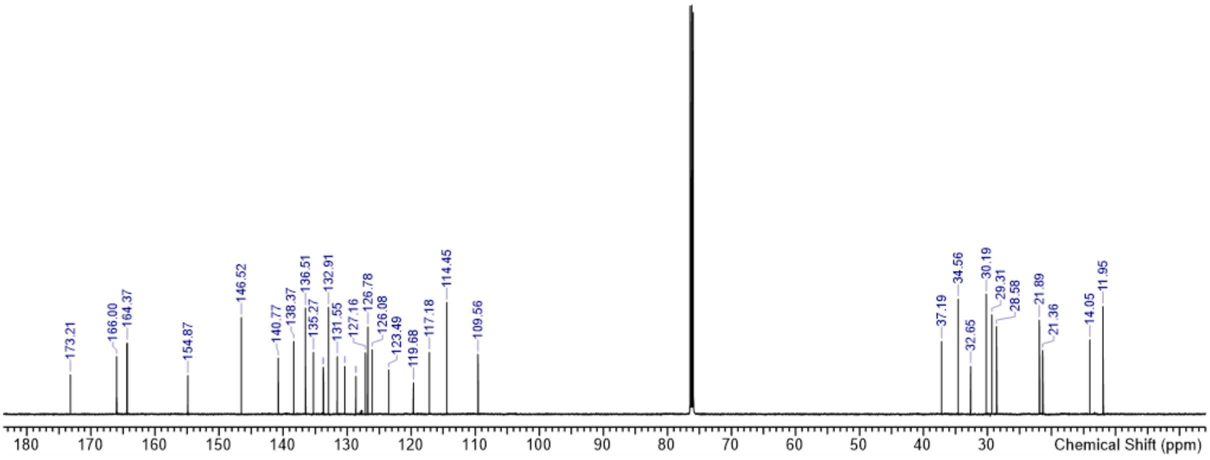

Figure S113: ^{13}C-NMR of 4. 151 MHz, Solvent: $CDCl_3$, 27 °C.
